# Poly-phenylene jacketed tailor-made dendritic phenylazomethine ligand for nanoparticle synthesis†

**DOI:** 10.1039/d1sc05661a

**Published:** 2022-03-18

**Authors:** Ken Albrecht, Maki Taguchi, Takamasa Tsukamoto, Tatsuya Moriai, Nozomi Yoshida, Kimihisa Yamamoto

**Affiliations:** Laboratory for Chemistry and Life Science (CLS), Institute of Innovative Research (IIR), Tokyo Institute of Technology Yokohama 226-8503 Japan yamamoto@res.titech.ac.jp; JST-ERATO, Yamamoto Atom Hybrid Project, Institute of Innovative Research (IIR), Tokyo Institute of Technology 4259 Nagatsuta, Midori-ku Yokohama 226-8503 Japan; Institute for Materials Chemistry, Engineering Kyushu University 6-1 Kasuga-Koen Kasuga-shi 816-8580 Fukuoka Japan albrecht@cm.kyushu-u.ac.jp

## Abstract

Synthesizing metal clusters with a specific number of atoms on a preparative scale for studying advanced properties is still a challenge. The dendrimer templated method is powerful for synthesizing size or atomicity controlled nanoparticles. However, not all atomicity is accessible with conventional dendrimers. A new tailor-made phenylazomethine dendrimer (DPA) with a limited number of coordination sites (*n* = 16) and a non-coordinating large poly-phenylene shell was designed to tackle this problem. The asymmetric dendron and adamantane core four substituted dendrimer (PPDPA16) were successfully synthesized. The coordination behavior confirmed the accumulation of 16 metal Lewis acids (RhCl_3_, RuCl_3_, and SnBr_2_) to PPDPA16. After the reduction of the complex, low valent metal nanoparticles with controlled size were obtained. The tailor-made dendrimer is a promising approach to synthesize a variety of metal clusters with desired atomicity.

## Abstract 2

A new design concept of a dendritic ligand with regulated number coordination sites is proposed, and capability as a template for nanoparticle synthesis is demonstrated.

## Graphical abstract

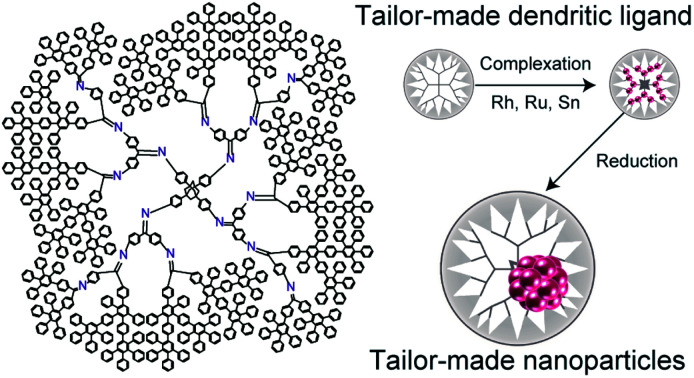

## Introduction

Metal nanoparticles are widely used in several applications and their property changes as a function of their size.^1,2^ However, in the sub-nanometer region, controlling the structure and atomicity is essential to control the property because the surface atoms will be dominant, and the particle will have a molecular electronic structure instead of a band-like electronic structure.^3,4^ Atomicity-controlled synthesis of nanoparticles (clusters) can be achieved with the gas-phase synthesis in combination with mass sorting techniques.^5–8^ This allowed the spectroscopic study of several metals to metal oxides nanoparticles and opened the door of cluster science. However, the limitation is the low yield and slow deposition rate of the clusters, leading to difficulties in measuring advanced properties such as catalytic properties and handling them practically. A liquid-phase synthesis is a promising approach for the preparative scale production of atomicity-controlled clusters suitable for observing advanced properties. Some so-called “magic-number” clusters can be obtained as a thermodynamically stable product on a relatively large scale.^9,10^ However, these clusters are usually covered with ligands, and not all atomicity is accessible. The ligand usually suppresses the catalytic reactivity, and the ligand structure dominates the electronic property. Therefore, a new method is eagerly anticipated to synthesize unprotected or weakly protected and atomicity-controlled clusters on a preparative scale for studying the bare property of the clusters.

Dendrimers^11–16^ that consist of multiple ligands can provide storage of a limited number of metal salts that lead to nanoparticle formation with a small size distribution.^17–21^ Phenylazomethine dendrimer (DPA)^22–28^ is a unique scaffold to assemble a specific number of Lewis acids and form weakly stabilized and kinetically trap nanoparticles to the internal space.^29^ DPA has a π-conjugated rigid backbone and a dense shell (sparse core).^30^ This character is suitable for trapping nanoparticles inside the dendrimer but keeping accessible space around the nanoparticle. As a result, the reactant can access the nanoparticle surface, and reactivity of DPA encapsulated nanoparticles will be maintained.^31,32^ However, due to the branched molecular structure and the stepwise radial complexation behavior, the number of Lewis acids that can be finely assembled into DPAs are usually limited to *n*, 3*n*, 7*n*, and 15*n* (*n* = core substitution number, see Fig.S1† for detail). Adopting an asymmetric core^33^ or inversion of the coordination sequence^34^ can allow other Lewis acid assembly numbers. However, this approach is far from covering all numbers in the range of 1 to 30 atoms, limiting the access to series of nanoparticles with each atomicity.

A straightforward solution to create precursors for nanoparticles with specific atomicity is to prepare a polynuclear complex^35,36^ consisting of a ligand with a specific number of coordination sites. DPA with a tailor-made number of azomethine units matches this criterion; however, low generation asymmetric DPAs are required to achieve low atomicity. This will cause a challenge in synthesis and the lack of “shell effect” necessary to preserve the formed nanoparticle inside the dendrimer. Here we will propose the concept of a new tailor-made DPA with regulated coordination sites and a large non-coordinating shell (polyphenylene). The synthesis of asymmetric dendron and dendrimer with exactly 16 coordination sites and the capability as nanoparticle template will be demonstrated. This will be a versatile approach to have dendritic ligands with regulated coordination sites and sufficient shell effect for nanoparticle synthesis.

## Results and discussion

**Chart 1:**
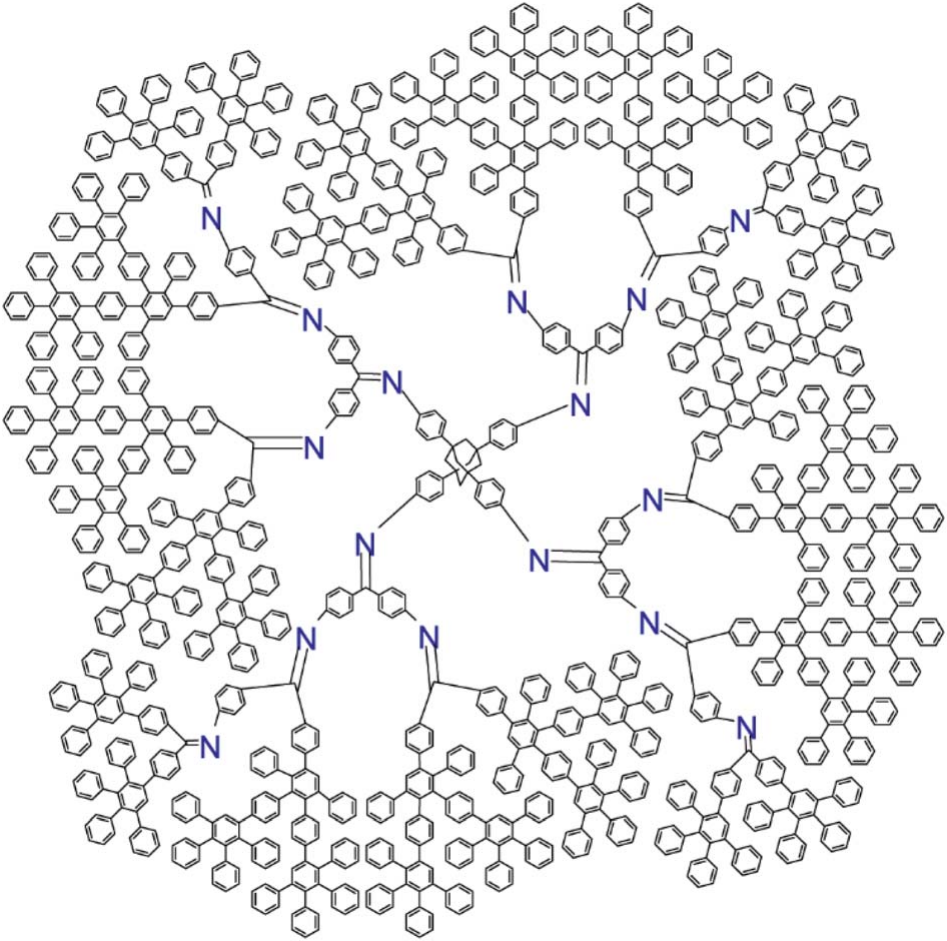
Structure of phenylazomethine dendrimer with exactly 16 coordination sites and polyphenylene jacket (PPDPA16).

The new DPA was designed to fulfill the following requirements; (1) having a regulated number of azomethine units, and (2) having a sufficient shell to kinetically trap the formed particles. Asymmetric phenylazomethine dendrimers with various phenylazomethine units can form a polynuclear complex with a specific number of Lewis acids. However, with this simple structure, the nanoparticle created in the dendrimers by reduction or oxidation of assembled Lewis acids will rapidly leach out the dendrimer scaffold. To provide a shell around the phenylazomethine units, a non-coordinating dendritic structure that is stable under the convergent synthesis of DPA, *i.e.*, the dehydration reaction with TiCl_4_ and oxidation with KMnO_4_, is required.^37^ Polyphenylene (PP) dendrimer is a rigid π-conjugated dendrimer consisting of benzene rings^38^ and has sufficient stability under the above reaction conditions. Phenylenevinylene dendrimers^39,40^ have a similar structure and were also considered as a candidate to cover DPA, but it was not stable under the KMnO_4_ oxidation process. Therefore, PP dendrimer was chosen as a shell to cover DPA, and the dendrimer was designed to have in total 4 times (DPA + PP) branching to form a jacket (Chart 1). The new dendrimer design concept using dendrons with asymmetric dendrons can generally be applied to synthesize dendrimers with various phenylazomethine units through controlling the combination and substitution number of dendrons.^36^

The new dendrimer with exactly 16 azomethine coordination sites with a PP shell was synthesized with the combination of divergent and convergent methods. First, the 1st and 2nd generation PP dendron was attached to a benzophenone unit with the divergent method.^41^ Next, the azomethine units were synthesized with the convergent method that the dehydration reaction between benzophenone and aniline is the key. Finally, the asymmetric dendron was reacted with an adamantane core to form the dendrimer with 16 coordination sites(Scheme S1–5†). Note that the tetraphenylmethane^30^ was too small to bind four dendrons due to the large steric hindrance of the dendrons and the dehydration reaction stopped after the 3-amine groups reacted. All compounds were characterized with ^1^H and ^13^C NMR, MALDI-TOF-MS, and elemental analysis. The solubility of the dendrimer in THF was low compared to conventional DPA but soluble in chloroform reflecting the character of the PP shell. The asymmetric dendron with 4 azomethine units requires longest synthetic route compared to other symmetric dendrons (3 and 7) that are required to synthesize dendrimers with various phenylazomethine units.^36^ With the success in the synthesis of PPDPA16, we have demonstrated that the design concept of coordination site regulated DPA can be realized.

**Fig. 1:**
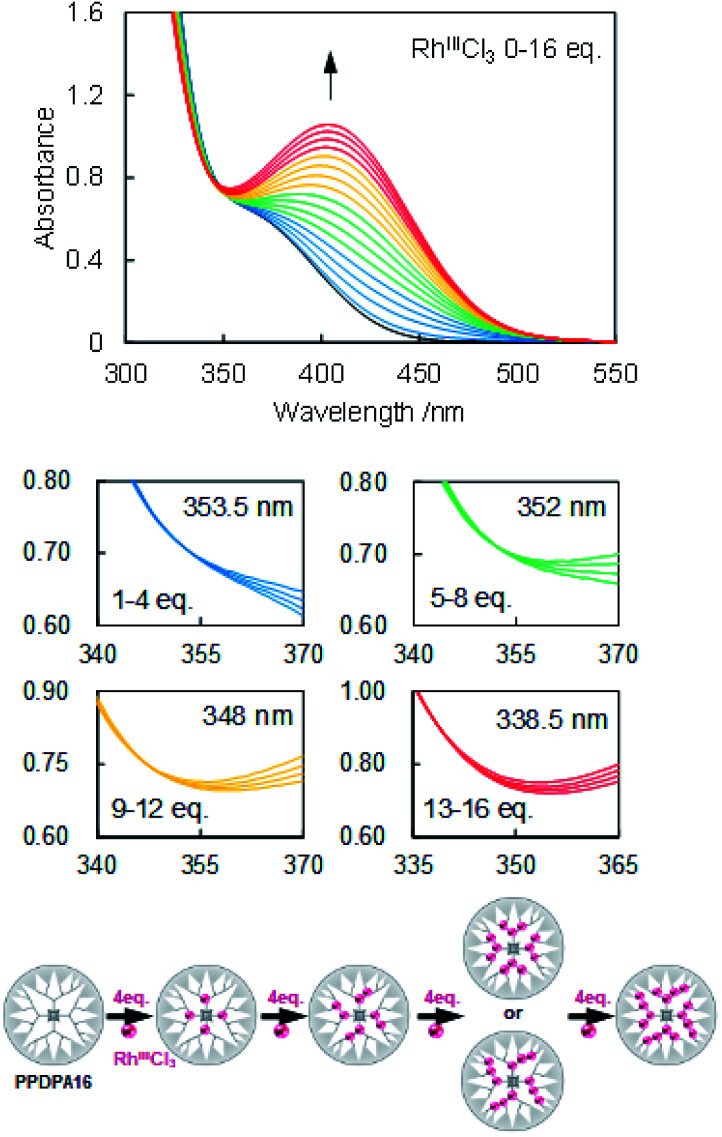
Accumulation of Rh^III^Cl_3_ to PPDPA16. (top) Change in UV-vis absorption spectra of PPDPA16 in DCM upon adding Rh^III^Cl_3_ in MeCN/MeOH = 250/1 up to 16 eq. ([PPDPA16] = *ca.* 5 μM, 20 °C). (down) Enlarged views during complexation with Rh^III^Cl_3_ in respective positions 0–4, 5–8, 9–12, and 13–16 eq. and the schematic illustration of the possible coordination sequence.

The coordination behavior of the dendrimer with RhCl_3,_ RuCl_3,_ and SnBr_2_ was determined by UV-vis titration (Fig. 1 and S3–5†). Upon adding RhCl_3_ to the dendrimer solution (5 μM, dichloromethane), typical change of the UV-vis spectra according to the complexation of Lewis acids to phenylazomethine, *i.e.*, the increase of the absorption at 400 nm and decrease at shorter wavelength was observed (Fig. S5 and 6†).^24^ The careful evaluation revealed the existence of 4 isosbestic points during the addition of 16 eq. of RhCl_3_. The spectra change has continued after the addition of more than 16 eq. without an isosbestic point. This is attributed to other reaction that is not coordination to phenylazomethine unit. The 4 isosbestic points until addition of 16 eq. indicate that RhCl_3_ coordinates to PPDPA16 in a stepwise manner. The isosbestic point changes every 4 eq. of RuCl_3_ and by comparison with previous reports, it is attributed to the stepwise complexation from the inner-layer to the outer-layer units.^24^ The sequence cannot be exactly determined, but possibly can be the sequence illustrated in Fig. 1 down. Considering that azomethine unit where another azomethine unit is bound to the outside has stronger basicity due to the electron donation.^36^ In the case of RuCl_3_ and SnBr_2,_ the UV-vis spectra of dendrimer solution also showed a similar trend (Fig. S3 and 4†). Most importantly, these data mean that PPDPA16 has the capability to exactly collect 16 metal atoms into the dendrimer scaffold.

**Fig. 2:**
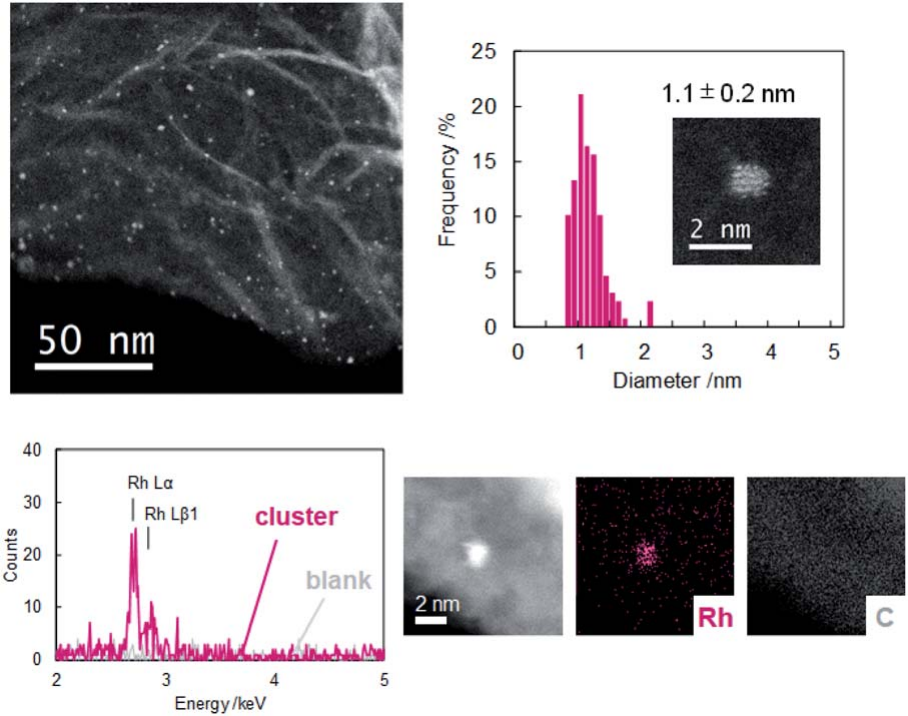
STEM image, histogram, and EDX analysis of the synthesized Rh nanoparticle.

Dendrimer templated Rh nanoparticles^42,43^ were synthesized by reducing PPDPA16-(RhCl_3_)_16_ complex. To the solution of the complex, NaBH_4_ methanol solution was added under vigorous stirring. The color change corresponding to decomplexation was observed, and immediately GNP (graphene nanopowder) dispersed in dichloromethane (1 mg/2 mL) was added to support dendrimer encapsulated nanoparticles. The dispersion was cast on a TEM grid, and HAADF-STEM images were obtained (Fig. 2). The image of Rh clusters with size of 1.1 ± 0.2 nm were obtained. The size corresponds well with a model of Rh16 cluster within the error range, and the EDX analysis has confirmed that the particle consists of Rh atoms. During obtaining the high-magnification image, the disassembly of the particle was observed due to the high energy of the electron beam. The image after disassembly of the one particle clearly showed that this particle consists of 16 bright spots (Fig. S11†). These results do not secure that all particles formed in PPDPA16 are exactly Rh_16_ particles, but most likely, the majority is Rh_16_ cluster weakly stabilized with the DPA and PP scaffold^31^ as expected from the dendrimer design.

The entire coordination and reduction process of RhCl_3_ in PPDPA16 was also investigated with XPS (Fig. S9†). RhCl_3_ showed the peak at a binding energy of 311.5 eV that is assigned to the 3d_5/2_ orbital. Coordination of 16RhCl_3_ to imine sites has shifted the binding energy and broadened the signals to the lower region (310.2 eV) due to the electron donation from the nitrogen lone pair. The following reduction shifted the binding energy to 307.5 eV, corresponding to low valent Rh metal (it is assumed that it mainly consists of 0 valent with some oxidized state).^26^ Further investigation and fitting of the XPS spectra of RhCl_3_ coordinating to the dendrimer revealed that the peak consists of three components with a 1 : 2 : 1 strength ratio (Fig. S9†). Considering the sequential coordination behavior and the difference in the basicity of each imine site, it is assumed that this reflects the number of RhCl_3_ that is coordinating to different imine sites. Namely, the peak at 308.6 eV with the lowest binding energy is the Rh atom with the most strong electron donated RhCl_3_ that corresponds to four RhCl_3_ coordinating to the innermost imine sites of PPDPA16. The peak at the highest binding energy (311.4 eV) is assigned to four RhCl_3_ coordinating to the weakest (low basicity) imine site. The intermediate peak at 310.2 eV is attributed to eight (four + four) RhCl_3_ coordinates second and third to PPDPA16. Overall, the XPS analysis confirmed the sequential coordination of RhCl_3_ to PPDPA16 and the reduction to metallic Rh particles.

The formation of Ru nanoparticle^44,45^ through the reduction of PPDA16-(RuCl_3_)_16_ complex was also performed similarly to Rh. The coordination of RuCl_3_ to PPDPA16 and reduction to low state valent was confirmed by XPS measurements(Fig. S10†). The binding energy of RuCl_3_ (Ru 3p_3/2_) was 463.9 eV and shifted to lower binding energy (462.1 eV) due to the electron donation from the imine nitrogen. The reduction of the complex further shifted the binding energy to 461.2 eV, indicating the reduction to a low valent state that mainly consists of 0 valent.^26^ The obtained particle size was determined from STEM images to be 1.0 ± 0.1 nm (Fig. S12†). This matches the size of Ru_16_ cluster within experimental error. These observations indicate that PPDPA16 has the capability to collect 16 Lewis acids through coordination to imine nitrogen, and reduction of the complex will give metal particles within a specific atomicity range.

To confirm the effectiveness of the PP jacket to inhibit aggregation of the nanoparticles, a reference experiment using DPA with 12 coordination sites without a PP jacket (DPA12, Fig. S2†) was performed. The UV-vis titration of DPA12 with RhCl_3_ and RuCl_3_ indicates that DPA12 has the capability to coordinate exactly 12 Lewis acids (Fig. S7 and 8†). These complexes were reduced, supported on GNP, and STEM images were obtained. The observed size of the particles was 1.4 ± 0.6 nm and 1.0 ± 0.2 nm for Rh and Ru particles, respectively (Fig. S13 and 14†). These values are indicating that the particles are undergoing aggregation. Furthermore, the same experiments were performed without any dendrimer. This experiment resulted in a large aggregate formation (Fig. S15†). This indicates that in the absence of the PP jacket, reduced metal salts will undergo aggregation and the existence of the large PP jacket is the key to form clusters with a specific atomicity range.

## Conclusions

In conclusion, phenylazomethine dendrimer with exactly 16 coordination sites covered with a polyphenylene dendrimer jacket was synthesized. This dendrimer (PPDPA16) can collect 16 Lewis acids through coordination to imine sites. Reduction of RhCl_3_ and RuCl_3_ complex with NaBH_4_ resulted in nanoparticles consisting of low valent metals with a narrow distribution. This is only one example of a tailor-made dendrimer with a specific number of coordination sites, but in principle, this strategy will lead to synthesizing a variety of dendrimers with regulated coordination sites and serve as a template for metal clusters with desired atomicity. The new concept of coordination site regulated dendrimer will lead to synthesizing the series of atomicity controlled nanoparticles. Further characterization and study on the electronic structure with EXAFS and catalytic property of series of nanoparticles will be conducted in the future.

## Data availability

The datasets used and/or analyzed during the current study are available from the corresponding author on reasonable request.

## Author contributions

MT., TT, TM, and NY collected experimental data. KA and KY conceptualized the project and supervised the investigation. KA designed the methodology, and wrote the manuscript with the help of MT and TT.

## Conflicts of interest

The authors declare no competing financial interest.

## Supplementary Material

SC-013-D1SC05661A-s001
