# Supplementary material for: Poly-phenylene jacketed tailor-made dendritic phenylazomethine ligand for nanoparticle synthesis
Source: Chem Sci. 2022 Mar 18;13(20):5813–7. doi: 10.1039/d1sc05661a (PMC9132029; doi:10.1039/d1sc05661a)
Supplement: SC-013-D1SC05661A-s001 [file SC-013-D1SC05661A-s001.pdf]

Electronic Supplementary Material (ESI) for Chemical Science.
This journal is © The Royal Society of Chemistry 2022

Poly-Phenylene Jacketed Tailor-Made Dendritic Phenylazome-thine Ligand for Nanoparticle Synthesis

Ken Albrecht, ‡*[abcd] Maki Taguchi, ‡[b] Takamasa Tsukamoto,[abd] Tatsuya Moriai,[a] Nozomi Yoshida,[a] and Kimihisa Yamamoto*[ab]

a. Laboratory for Chemistry and Life Science (CLS), Institute of Innovative Research (IIR), Tokyo Institute of Technology, Yokohama 226-8503 (Japan).
Email: yamamoto@res.titech.ac.jp

b. JST-ERATO, Yamamoto Atom Hybrid Project, Institute of Innovative Research (IIR), Tokyo Institute of Technology, 4259Nagatsuta, Midori-ku,Yokohama226-8503(Japan)

c. Institute for Materials Chemistry and Engineering Kyushu University, 6-1 Kasuga-Koen Kasuga-shi, 816-8580, Fukuoka, Japan
Email: albrecht@cm.kyushu-u.ac.jp

d. Present address: JST-PRESTO, Kawaguchi 332-0012(Japan)

‡ These authors contributed equally.

Electronic Supplementary Information

Table of Contents

1. Experimental section S2
2. Accumulation of Lewis acids to DPA S3
3. Synthesis S4
4. UV-vis titration S20
5. XPS Spectra S24
6. STEM images S26
7. Characterization data S29

1. Experimental section

Chemicals. Rhodium trichloride trihydrate was purchased from AlfaAesar. Ruthenium trichloride was purchased from Tokyo Chemical Industry Co., Ltd. Tin dibromide was purchased from Aldrich. High surface area graphene nanopowder (grade AO-1) was purchased from ALLIANCE Biosystems, Inc. All other chemicals were purchased from Kanto Kagaku Co., Ltd. or Aldrich, and used without further purification (all solvents for the UV-vis titration and cluster formation were of the dehydrated grade).

General. The NMR spectra were obtained using a Bruker AVANCE Ⅲ 400 (400MHz). 1H NMR and 13C NMR were measured with TMS as the internal standard. The MALDI TOF-MS and APCI TOF-MS data were obtained using Shimadzu/ Kratos AXIMA CFR plus in the linear positive ion mode or Bruker MicrOTOF II, respectively. Dithranol was used as the matrix for MALDI-TOF-MS. The elemental analysis was performed at the Center for Advanced Materials Analysis, Technical Department, TIT. A preparative scale gel permeation chromatography, LC-908 (Japan Analytical Industry Co., Ltd.), was used to isolate each compound with THF as the eluent. The UV-vis spectra were recorded using a Shimadzu UV-3100, UV-3150, or UV-3600 spectrometer with a quartz cell having a 1cm optical length at 20ºC in a glove box under Ar atmosphere. The STEM images were recorded using JEOL JEM-ARM200F equipped with an energy dispersive X-ray spectrometry (EDS) analyzer (acceleration voltage: 80 kV). XPS was recorded by a Shimadzu ESCA-3400 with Mg Kα (10 kV, 20 mA) using as an X-ray source. The binding energy was standardized using the Au $4f_{7/2}$ peak at 84.0 eV.

Titration of complexation on dendrimers on UV-vis absorption spectra. A 5.0×10^{-6} M dendrimer solution in dichloromethane for $RhCl_3$ and $RuCl_3$ or 1.0×10^{-5} M solution in dichloromethane/acetonitrile=2:1 for $SnBr_2$ (3 mL, 20 °C) was prepared in a 1 cm optical quartz cell. A 3.0×10^{-3} M solution (acetonitrile for $RuCl_3$ and $SnBr_2$, acetonitrile/methanol=250:1 for $RhCl_3$) was added to a 3 mL solution of dendrimer in a stepwise fashion from 0 to 20 equiv. of a dendrimer molecule, respectively. The absorption of metal salt was subtracted by adding the same amount to the reference cell.

Template synthesis of clusters. The preparation method of the complexes of dendrimers and metal salts ($RhCl_3$ and $RuCl_3$) was the same as those for the UV-vis absorption measurement mentioned above. 60 equiv of $NaBH_4$ (300 mM, methanol) on the accumulated metal salts was added to the complex solution with stirring. For the STEM observation and EDS analysis, a suspension of graphene nanopowder (1 mg in 2 mL of dichloromethane) was quickly added to the reaction mixture and stirred for 1 min. The clusters supported on graphene in solution were dropped onto a copper grid with a carbon film and dried overnight under a vacuum. For the XPS measurement, a 5 μL portion of the fresh cluster solution was then dropped 10 times (total 50 μL) onto a glassy carbon substrate (5 mm × 5 mm). After drying, the XPS analysis was conducted as soon as possible. All operations were carried out under an inert atmosphere (N_2).

2. Accumulation of Lewis acids to DPA

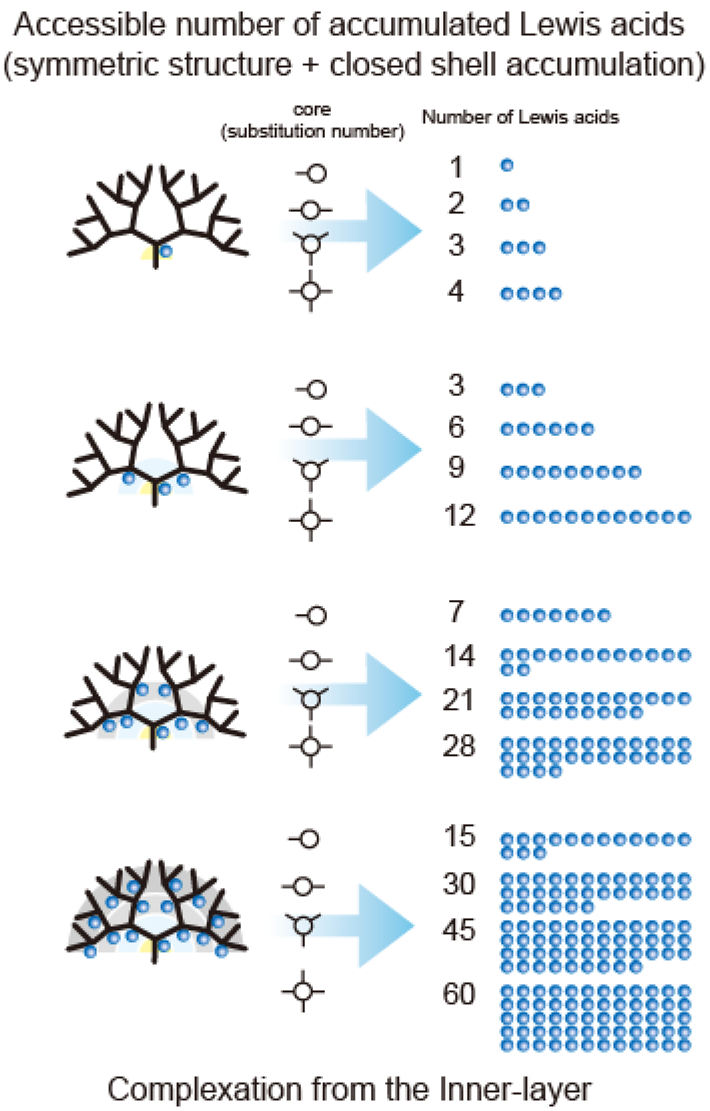

Figure S1. Accumulation of Lewis acids from the inner-layer to symmetric DPA with 1 to 4 substituted structures. Accessible numbers are 1, 2, 3, 4, 6, 7, 9, 12, 14, 15, 21, 28, 30, 45, and 60. Other numbers can be also accumulated, but the "closed shell" accumulation that is shown above will have a small statistical distribution compared to other multi-ligand systems that have equal binding sites. Note that in practical use the one and two substituted dendrimers have not enough shell effect to kinetically trap the formed nanoparticles.

3. Synthesis

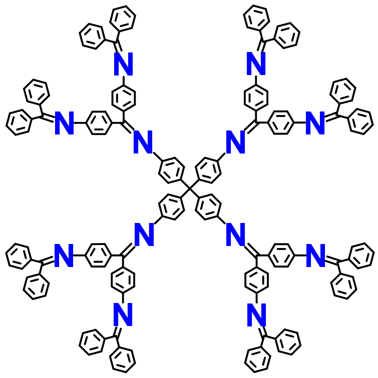

Figure S2. Structure of DPA12.

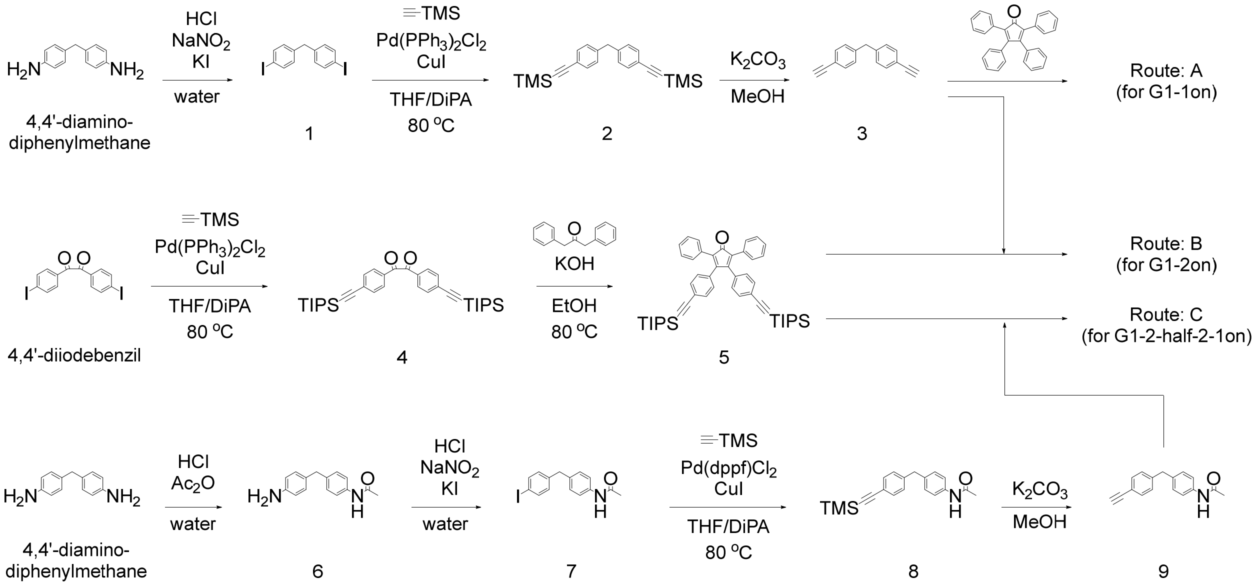

Scheme S1. Synthesis of starting materials.

Route: A

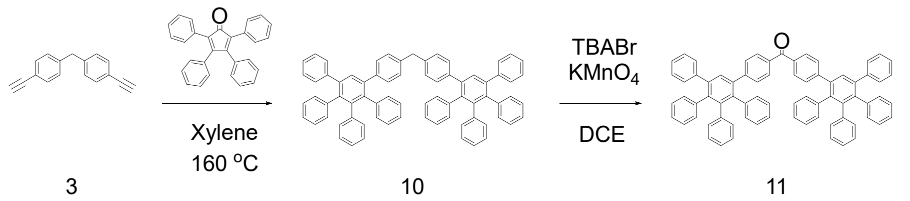

Route: B

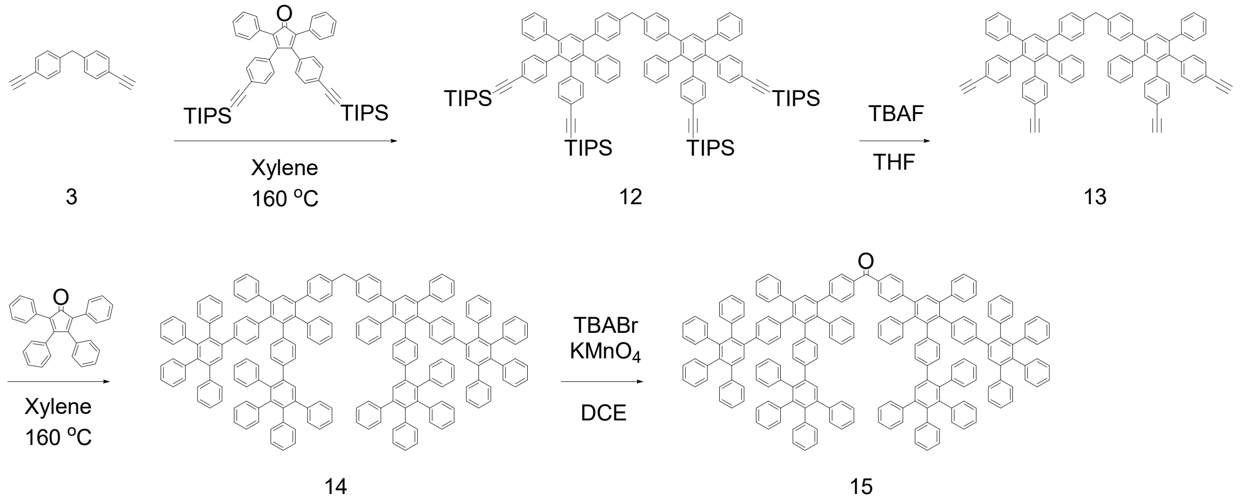

Scheme S2. Synthesis of polyphenylene jacketed dendrons G1-1on (Route: A) and G1-2on (Route: B).

Route: C

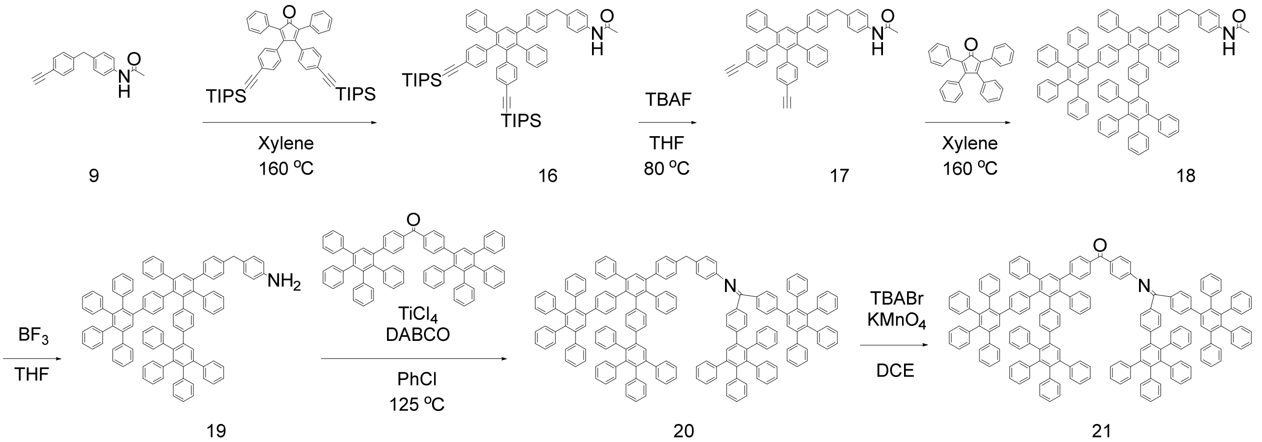

Scheme S3. Synthesis of polyphenylene jacketed dendron G1-1-half-2-1on (Route: C).

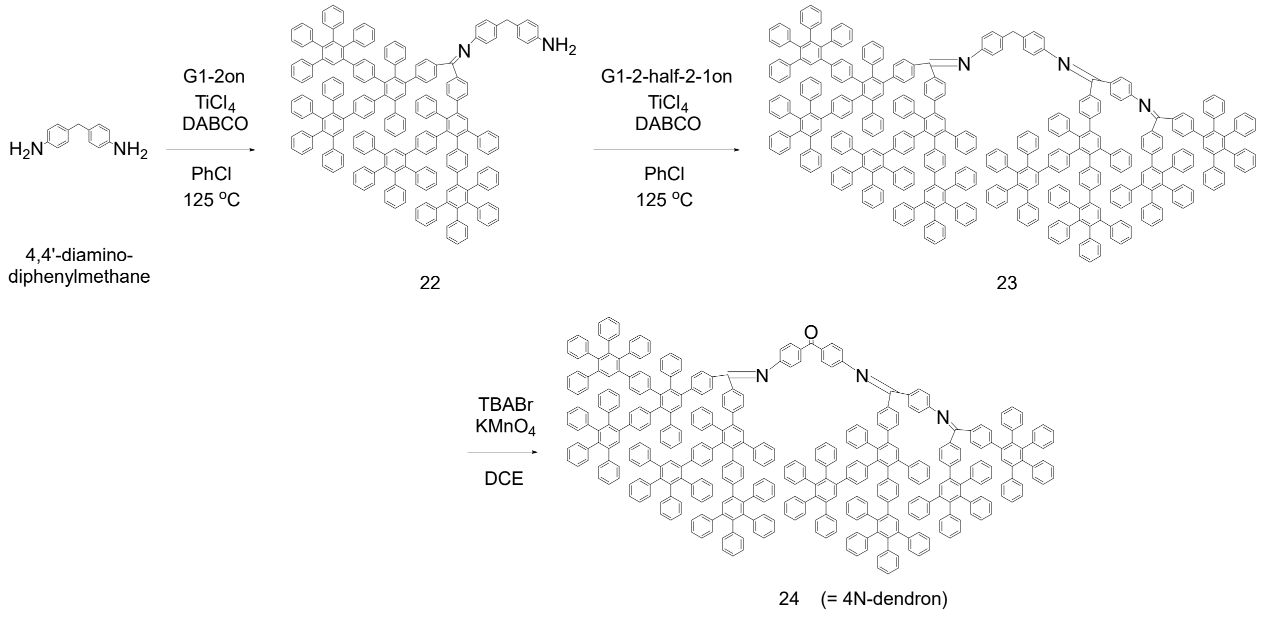

Scheme S4. Synthesis of polyphenylene jacketed dendron G2-2-quater-3-1on.

4,4'-Diiodo-diphenylmethane (1).

To 500 ml of distilled water in a 3000 ml beaker containing a stick-shaped magnetic stirrer tip which was completely cooled at 0 ºC on the ice bath, 20 ml of Hydrochloride was added. 4,4'-diamino-diphenylmethane (10.0 g, 50.4 mmol) which was milled was added to the stirred mixture. The mixture was stirred at 0 ºC for 30 min. Then, sodium nitrite (10.0 g, 145 mmol) was added slowly and the mixture was stirred for 60 min. And then, potassium iodide (50.0 g, 301 mmol) which was milled was added slowly to the mixture at 0 ºC. After 15-20 min, agglomerated material on the side of a beaker was dissolved in dichloromethane. The mixture was stirred at r.t. overnight. The resulting solution was neutralized with aqueous potassium carbonate. The organic phase was extracted with dichloromethane, washed with aqueous sodium thiosulfate several times and with brine, respectively, and then dried with magnesium sulfate. The solution was filtrated and evaporated. The crude was adsorbed into Celite powder and then isolated through a silica-gel short column chromatography eluting with n-hexane to afford 12.5 g (yield 58.7%) of white powder.

^{1}H NMR (400 MHz, $CDCl_3$, 29.4 ºC, TMS):

δ [ppm] = 7.6 (d, J = 8.3 Hz, 4H), 6.90 (d, J = 8.3 Hz, 4H), 3.85 (s, 2H).

MALDI TOF-MS (Matrix: Dithranol): Calcd: 420.89 ($[M+H]^+$), Found: 420.70.

4,4'-Bis(trimethylsilylethynyl)-diphenylmethane (2).

A 300 ml flask (three-necked round-bottom flask) containing 4,4'-diiodo-diphenylmethane (**1**) (5.000 g, 11.90 mmol), copper (I) iodide (0.0906 g, 0.476 mmol) and bis(triphenylphosphine) palladium (II) dichloride (0.835 g, 1.190 mmol) was degassed and flushed with argon gas. 220 ml of tetrahydrofuran (anhydrate grade) and 40 ml of diisopropylamine were added by using a syringe. To the stirred mixture, 3.6 ml of trimethylsilyl acetylene (2.57 g, 26.2 mmol) was added slowly by using a syringe. Then, the mixture was refluxed at 80 ºC for

two nights. The resulting solution was evaporated. The crude was adsorbed into Celite powder and put on a silica gel column (round 10 cm, height 12 cm) packed with n-hexane. A purification with column chromatography eluting with n-hexane (which should not be pressured well) afforded 3.152 g (yield 73.4%) of ivory powder.

^{1}H NMR (400 MHz, $CDCl_3$, 27.4 ºC, TMS):

δ [ppm] = 7.37 (d, J = 8.1 Hz, 4H), 7.07 (d, J = 8.1 Hz, 4H), 3.94 (s, 2H), 0.24 (s, 18H).

^{13}C NMR (100 MHz, $CDCl_3$, 25.9 ºC, TMS):

δ [ppm] = 140.92, 131.97, 128.66, 120.90, 104.87, 93.71, 41.49, -0.18.

MALDI TOF-MS (Matrix: Dithranol): Calcd: 360.18 ($[M+H]^+$), Found: 360.63.

Anal. Calcd. for $C_{23}H_{28}Si_2$: C, 76.60; H, 7.83. Found: C, 76.41; H, 7.92.

4,4'-Diethynyl-diphenylmethane (3).

A 300 ml flask (three-necked round-bottom flask) containing 4,4'-bis(trimethylsilylethynyl) -diphenylmethane (**2**) (0.420 g, 1.165 mmol) and potassium carbonate (0.966 g, 6.987 mmol) was degassed and flushed with argon gas. 150 ml of methanol was added. The mixture was stirred at r.t. for more than 5 hours and then evaporated. The crude was neutralized with dil. HCl, extracted with dichloromethane, washed with brine and then dried with magnesium sulfate. The solution was filtrated and evaporated to afford 0.362 g of yellow material (rough yield). The crude material was instantly used for the next step (Diels-Alder cycloaddition). The product can be purified by a silica gel column chromatography eluting with n-hexane to be white powder.

^{1}H NMR (400 MHz, CD_2Cl_2, 25.3 ºC, TMS):

δ [ppm] = 7.41 (d, J= 7.7 Hz, 2H), 7.11 (d, J= 7.7 Hz, 2H), 3.97 (s, 2H), 3.09 (s, 2H).

^{13}C NMR (100 MHz, CD_2Cl_2, 26.6 ºC, TMS):

δ [ppm] = 142.06, 132.65, 129.35, 120.40, 83.78, 77.21, 41.96.

APCI-MS (positive): Calcd: 217.10 ($[M+H]^+$), Found: 217.20.

4,4'-Bis(triisopropylsilylethynyl)benzyl (4).

A 1000 ml flask (three-necked round-bottom flask) containing 4,4'-dibromobenzil (9.752 g, 26.50 mmol), copper (I) iodide (0.202 g, 1.06 mmol), and bis(triphenylphosphine) palladium (II) dichloride (1.862 g, 2.653 mmol) was degassed and flushed with argon gas. 400 ml of tetrahydrofuran (anhydrate grade) and 80 ml of diisopropylamine were added by using a syringe. To the stirred mixture, 13.0 ml of triisopropylsilyl acetylene (10.6 g, 58.3 mmol) was added slowly by using a syringe. Then, the mixture was refluxed at 75 ºC for two nights. The resulting solution was evaporated. The crude was adsorbed into Celite particle for column and isolated by using a silica gel flash column chromatography [eluting with n-hexane and ethyl acetate mixture (98:2)]. It afforded 14.534 g (yield 96.0%) of yellow oil-like material.

^{1}H NMR (400 MHz, $CDCl_3$, 28.9 ºC, TMS):

δ [ppm] = 7.92 (d, J = 8.5 Hz, 4H), 7.60 (d, J = 8.6 Hz, 4H), 1.16 (s, 42H).

APCI-MS (positive): Calcd: 589.36 ($[M+H+H_2O]^+$), Found: 589.27.

3,4-Bis(4-triisopropylsilylethynyl)phenyl-2,5-diphenyl-cyclopenta-2,4-dien-1-one (5).

A 1000 ml flask (three-necked round-bottom flask) containing 4,4'-bis(triisopropylsilylethynyl) benzyl (**4**) (14.32 g, 25.08 mmol), 1,3-Diphenyl-2-propanone (4.800 g, 22.83 mmol), and ethyl alcohol (50 ml) was degassed and flushed with argon gas and reactant was completely dissolved in ethanol at 80 ºC. To the mixture which had been stirred at room temperature, 140 ml of ethanol solution containing potassium hydroxide (0.769 g, 13.7 mmol) was added from a 200 ml dropping funnel. The mixture was stirred at 80 ºC for 12 min and then cooled down in the refrigerator overnight. The resulting cold solution was filtrated to afford chocolate-like materials, which was washed well with 5% ethanol in water to afford cocoa-like powder 14.314 g (yield 84.0%). The filtrate solution was extracted with diethyl ether, washed with brine, and dried with magnesium sulfate. Then the solution was filtrated and evaporated. The crude was adsorbed into Celite particle for column and isolated by using a silica gel flush column chromatography [eluting with n-hexane and chloroform mixture (70:30)]. It afforded 1.748 g (yield 10.4%) of wine-red amorphous. cocoa-like powder and wine-red amorphous were identical materials by evaluation of NMR spectra. The total amount was 16.062 g (yield 94.4%).

^{1}H NMR (400 MHz, CD_2Cl_2, 25.2 ºC, TMS):

δ [ppm] = 7.34-7.26 (m, 10H), 7.21-7.19 (m, 4H), 6.93-6.87 (m, 4H), 1.12 (s, 42H).

^{13}C NMR (100 MHz, CD_2Cl_2, 26.2 ºC, TMS):

δ [ppm] = 200.11, 153.94, 133.49, 133.40, 132.22, 132.02, 131.09, 130.98, 130.55, 130.25, 129.78, 129.74, 128.51, 128.10, 126.54, 126.47, 126.39, 124.13, 122.70, 107.00, 92.82, 78.73, 18.82, 11.74.

APCI-MS (positive): Calcd: 745.43 ($[M+H]^+$), Found: 745.45.

4-Acetamide-4'-amino-diphenylmethane (6).

To a 1000 ml beaker containing a stick-shaped magnetic stirrer tip were added 200 ml of distilled water and 7.0 ml of hydrochloride. To the stirred mixture was added 4,4'-diamino-diphenylmethane (9.83 g, 49.58 mmol) which was milled, before it was rinsed with 50 ml of ethanol (99.5% grade). The mixture was heated at 80 ºC. Acetic anhydride (4.2 g, 41.1 mmol) was extremely slowly dropwise added to the stirred mixture at 80 ºC. When the pearl-rose material occurred in the reaction solution, 80 ºC water was added to precipitate bi-acetylated derivative. The pink precipitate was removed by hot filtration and washed with ethanol 8 ml/ water 50 ml solution. The yellow filtrate was heated at 70 ºC. Potassium acetate (10.1 g, 0.103 mol) was dissolved in 20 ml of distilled water, and then dropwise added to the filtrated solution at 70 ºC. The resulting solution was cooled to 0 ºC and stirred for 2 hours. The precipitated white solid was collected by a cold filtration and washed with ethanol 7 ml / water 43 ml cold solution twice, and then dried at room temperature under diminished pressure. It afforded 5.83 g (yield 48.9%) of beige solid.

^{1}H NMR (400 MHz, $CDCl_3$, 31.4 ºC, TMS):

δ [ppm] = 7.37 (d, J = 8.24 Hz, 2H), 7.18 (s, 1H), 7.11 (d, J = 8.2 Hz, 2H), 6.94 (d, J = 8.2 Hz, 2H), 6.61 (d, J = 8.24 Hz, 2H), 3.82 (s, 2H), 3.56 (br, 2H), 2.14 (s, 3H).

MALDI TOF-MS (Matrix: Dithranol): Calcd: 241.13 ($[M+H]^+$), Found: 241.10.

4-Acetamide-4'-iodo-diphenylmethane (7).

10 ml of Hydrochloride was added to 500 ml of distilled water in a 3000 ml beaker containing a stick-shaped magnetic stirrer tip. 4-acetamide-4'-amino-diphenylmethane (**6**) (10.27 g, 42.73 mmol) was added to the mixture. The mixture was stirred at 0 ºC for 30 min. Then, sodium nitrite (4.000 g, 57.96 mmol) was slowly added and then stirred for 60 min at 0 ºC. To the 0 ºC mixture was extremely slowly added potassium iodide (20.00 g, 120.5 mmol) which was milled. The reaction solution was gradually warmed to room temperature and stirred overnight. Chloroform was added to dissolve agglomerated material in the reaction solution. The resulting solution was neutralized with aqueous potassium carbonate. The organic phase was extracted with chloroform and washed with aqueous sodium thiosulfate several times, and then washed with brine. The solution was dried with magnesium sulfate before it was filtrated and evaporated. The product was purified by using a silica gel flush column chromatography [eluting with n-hexane and ethyl acetate mixture (gradient from 100:0 to 50:50)] and the precipitation with methanol to afford 10.78 g (yield 71.8%) of pale orange solid.

^{1}H NMR (400 MHz, $CDCl_3$, 27.9 ºC, TMS):

δ [ppm] = 7.59 (d, *J* = 8.2 Hz, 2H), 7.40 (d, *J* = 8.3 Hz, 2H), 7.16 (s, 1H), 7.10 (d, *J* = 8.3 Hz, 2H), 6.91 (d, *J* = 8.2 Hz, 2H), 3.87 (s, 2H), 2.16 (s, 3H).

^{13}C NMR (100 MHz, $CDCl_3$, 26.6 ºC, TMS):

δ [ppm] = 137.52, 130.93, 129.41, 120.15, 40.79.

APCI-MS (negative): Calcd: 350.00 ($[M-H]^-$), Found: 349.36.

Anal. Calcd. for $C_{15}H_{14}INO$: C, 51.30; H, 4.02; N, 3.99. Found: C, 51.60; H, 4.10; N, 3.99.

4-Acetamide-4'-(trimethylsilylethynyl)-diphenylmethane (8).

A 1000 ml flask (three-necked round-bottom flask) containing 4-acetamide-4'-iodo-diphenylmethane (**7**) (7.600 g, 21.64 mmol), copper (I) iodide (0.370 g, 1.95 mmol) was degassed and flushed with argon gas. Under argon gas flowing, [1,1-bis(diphenylphosphino) ferrocene] palladium (II) dichloride: dichloromethane complex (; Pd(dppf)Cl_2:CH_2Cl_2) (3.53 g, 4.34 mmol) was carefully added. To the mixture were added 200 ml of tetrahydrofuran (anhydrate grade) and 6.0 ml of trimethylsilyl acetylene (4.26 g, 43.4 mmol) by using a syringe. To the stirred mixture was added 40 ml of diisopropylamine by using a syringe. Then, the mixture was refluxed at 75 ºC for two nights. The resulting solution was filtered and evaporated. The crude was dissolved in tetrahydrofuran and adsorbed into Celite particle for column and isolated by using a silica gel flush column chromatography [eluting with n-hexane and ethyl acetate mixture (gradient from 100:0 to 50:50)] and the precipitation with n-hexane to afford 6.30 g (yield 90.5%) of cream-color powder.

^{1}H NMR (400 MHz, $CDCl_3$, 25.3 ºC, TMS):

δ [ppm] = 7.38 (t, *J* = 8.9 Hz, 4H), 7.17 (s, 1H), 7.09 (d, *J* = 7.9 Hz, 4H), 3.92 (s, 2H), 2.15 (s, 3H), 0.23 (s, 9H).

^{13}C NMR (100 MHz, $CDCl_3$, 26.0 ºC, TMS):

δ [ppm] = 168.15, 141.64, 136.59, 136.09, 132.10, 129.44, 128.79, 120.92, 120.14, 105.13, 93.75, 41.20, 24.54, 0.00.

APCI-MS (positive): Calcd: 322.16 ($[M+H]^+$), Found: 322.16.

Anal. Calcd. for $C_{20}H_{23}NOSi$: C, 74.72; H, 7.21; N, 4.36. Found: C, 74.45; H, 6.95; N, 4.32.

4-Acetamide-4'-ethynyl-diphenylmethane (9).

A 25 ml schlenk flask containing 4-acetamide-4'-(trimethylsilylethynyl)-diphenylmethane (**8**) (0.159 g, 0.495 mmol) and potassium carbonate (0.205 g, 1.48 mmol) was degassed and flushed with argon gas. 10 ml of methanol was added. The mixture was stirred at r.t. overnight and then evaporated. The crude was dissolved in chloroform and neutralized with dil. HCl. The organic phase was extracted with chloroform and ethyl acetate. The organic phase was washed with brine, combined, and then dried with magnesium sulfate. The solution was filtered and evaporated. The product was isolated by twice silica gel flash column chromatography [eluting with n-hexane and toluene mixture (80:20)] and eluting with tetrahydrofuran. And then, the precipitation with n-hexane afforded 81.2 mg (yield 65%) of white powder.

^{1}H NMR (400 MHz, CD_2Cl_2, 25.2 ºC, TMS):

δ [ppm] = 7.41-7.39 (m, 4H), 7.27-7.11 (m, 5H), 3.94 (s, 2H), 3.09 (s, 1H), 2.10 (s, 3H).

^{13}C NMR (100 MHz, CD_2Cl_2, 26.4 ºC, TMS):

δ [ppm] = 168.43, 142.85, 136.93, 136.83, 132.59, 129.72, 129.28, 120.38, 120.18, 83.88, 77.06, 41.52, 24.68.

APCI-MS (negative): Calcd: 248.11 ($[M-H]^-$), Found: 248.19.

Anal. Calcd. for $C_{17}H_{15}NO$: C, 81.90; H, 6.06; N, 5.62. Found: C, 81.55; H, 6.20; N, 5.58.

4,4'-Bis(2,3,4,5-tetraphenyl-phenyl)-diphenylmethane (; called as "pre-G1-1") (10).

A 200 ml flask (three-necked round-bottom flask) containing 4,4'-diethynyl-diphenylmethane (**3**) (1.325 g, 6.128 mmol) and 2,3,4,5-tetraphenyl-cyclopenta-2,4-dien-1-one (4.97 g, 12.9 mmol) was degassed and flushed with argon gas. 60 ml of xylene (anhydrate grade, *o*-, *m*-, *p*-substituted mixture) was added by using a syringe. The mixture was refluxed at 160 ºC for three nights then evaporated. The crude was dissolved in chloroform and spread into acetone. The white precipitate was filtered and washed with acetone to afford 5.09 g (yield 89%) of pearl-white powder.

^{1}H NMR (400 MHz, CD_2Cl_2, 26.9 ºC, TMS):

δ [ppm] = 7.45 (s, 2H), 7.15 (s, 10H), 7.07 (d, J = 7.8 Hz, 4H), 6.94 (br, 16H), 6.87-6.86 (m, 14H), 6.82 (m, 4H), 3.82 (s, 2H).

^{13}C NMR (100 MHz, CD_2Cl_2, 28.2 ºC, TMS):

δ [ppm] = 142.27, 142.23, 141.18, 140.97, 140.67, 140.63, 140.02, 139.74, 139.62, 131.96, 131.88, 131.64, 130.39, 130.34, 128.48, 127.94, 127.23, 126.93, 126.63, 125.97, 125.69, 41.35.

MALDI TOF-MS (Matrix: Dithranol): Calcd: 929.41 ($[M+H]^+$), Found: 928.56.

Anal. Calcd. for $C_{73}H_{52}$: C, 94.36; H, 5.64. Found: C, 94.07; H, 5.53.

4,4'-Bis(2,3,4,5-tetraphenyl-phenyl)-diphenylmethanone (; called as "G1-1on") (11).

Pre-G1-1 (**10**) (10.00 g, 10.76 mmol) and tetrabutylammonium bromide (138.8 g, 0.4305 mol) were dissolved in 570 ml of 1,2-dichloroethane. To the mixture stirred at 0 ºC was added potassium permanganate (68.0 g, 0.430 mol). Then the mixture was stirred at r.t. for 8 days. The resulting solution was quenched with aqueous sodium hydrogen sulfite. The organic phase was extracted with chloroform and washed with brine and then dried with magnesium sulfate. The solution was filtrated and evaporated. The product was isolated by a

silica gel short column chromatography (eluting with chloroform) and purified by precipitation with methanol. It afforded 9.826 g (yield 96.8%) of white powder.

^{1}H NMR (400 MHz, CD_2Cl_2, 27.3 ºC, TMS, chloroform):

δ [ppm] = 7.55 (m, 2H), 7.51 (d, *J* = 8.0 Hz, 4H), 7.25 (d, *J* = 8.0 Hz, 4H), 7.17 (s, 10H), 6.94 (m, 12H), 6.88 (m, 14H), 6.84 (m, 4H).

^{13}C NMR (100 MHz, CD_2Cl_2, 28.2 ºC, TMS):

δ [ppm] = 196.04, 146.48, 142.40, 142,08, 141.40, 140.70, 140.43, 140.41, 140.21, 139.78, 135.89, 131.92, 131.88, 131.84, 131.37, 130.31, 130.20, 129.63, 128.00, 127.42, 127.27, 127.00, 126.77, 126.27, 126.10, 125.83.

MALDI TOF-MS (Matrix: Dithranol): Calcd: 943.39, Found: 943.63.

Anal. Calcd. for $C_{73}H_{50}O$: C, 92.96; H, 5.34. Found: C, 92.86; H, 5.55.

4,4'-Bis(3,4-bis(4-triisopropylsilylethynylphenyl)-2,5-diphenyl-phenyl)-diphenylmethane (12).

A 100 ml flask (three-necked round-bottom flask) containing 4,4'-diethynyl-diphenylmethane (**3**) (1.13 g, 5.22 mmol, crude material) and 3,4-bis(4-triisopropylsilylethynyl)phenyl-2,5-diphenyl-cyclopenta-2,4-dien -1-one (**5**) (10.00 g, 13.42 mmol) was degassed and flushed with argon gas. 100 ml of xylene (anhydrate grade, *o*-, *m*-, *p*-substituted mixture) was added by using a syringe. The mixture was refluxed at 175 ºC for two nights then evaporated. The crude was dissolved in diethyl ether and adsorbed into Celite particle for column and isolated by using a silica gel flush column chromatography [eluting with n-hexane and toluene mixture (80:20)]. It afforded 5.002 g (yield 58%) of beige amorphous.

^{1}H NMR (400 MHz, $CDCl_3$, 26.1 ºC, TMS):

δ [ppm] = 7.52 (s, 2H), 7.20-7.07 (m, 14H), 7.01 (t, *J* = 7.6 Hz, 8H), 6.96-6.91 (m, 6H), 6.88 (d, *J* = 8.1 Hz, 4H), 6.81-6.76 (m, 8H), 6.71 (d, *J* = 8.1 Hz, 4H), 3.81 (s, 2H), 1.08 (d, *J* = 3.6 Hz, 84H).

^{13}C NMR (100 MHz, $CDCl_3$, 26.9 ºC, TMS):

δ [ppm] = 141.42, 141.03, 140.82, 140.49, 140.09, 139.65, 139.31, 139.22, 139.00, 138.29, 131.90, 131.77, 131.46, 131.34, 131.26, 130.96, 130.68, 129.88, 129.34, 128.22, 127.79, 127.16, 126.44, 125.80, 120.78, 120.54, 107.41, 107.36, 90.17, 89.97, 40.98.

MALDI TOF-MS (Matrix: Dithranol): Calcd: 1650.95 ($[M+H]^+$), Found: 1650.93.

Anal. Calcd. for $C_{117}H_{132}Si_4$: C, 85.13; H, 8.06. Found: C, 84.80; H, 8.16.

4,4'-Bis(3,4-bis(4-ethynylphenyl)-2,5-diphenyl-phenyl)-diphenylmethane (13).

A 200 ml flask (three-necked round-bottom flask) containing 4,4'-Bis(3,4-bis(4-ethynylphenyl) -2,5-diphenyl-phenyl)-diphenylmethane (**12**) (1.350 g, 0.8178 mmol) was degassed and flushed with argon gas. 50 ml of tetrahydrofuran was added. To the stirred mixture was added 4.90 ml of 1.0M tetrabutylammonium fluoride solution in tetrahydrofuran (4.90 mmol) by using a syringe. After stirring for 2 hrs, the resulting solution was quenched with K_2CO_3 aq. The organic phase was extracted with dichloromethane. The organic phase was washed with brine and dried with magnesium sulfate. The solution was filtrated and evaporated. The crude was dissolved in tetrahydrofuran and precipitated in methanol to afford 0.83 g of beige material. The crude material was instantly used for the next step (Diels-Alder cycloaddition). The product can be purified by gel permeation chromatography (chloroform) to be pale beige powder.

^{1}H NMR (400 MHz, DMSO-d_6, 25.2ºC, TMS):

δ [ppm] = 7.41 (s, 2H), 7.21-7.13 (m, 10H), 7.06 (t, J = 6.7 Hz, 8H), 7.00-6.94 (m, 14H), 6.89-6.82 (m, 12H), 4.07 (s, 2H), 4.04 (s, 2H), 3.77 (s, 2H).

^{13}C NMR (100 MHz, DMSO-d_6, 26.2 ºC, TMS):

δ [ppm] = 140.71, 140.62, 140.61, 140.35, 140.20, 140.15, 139.11, 138.85, 138.47, 137.86, 131.34, 131.22, 130.97, 130.91, 130.37, 130.03, 129.53, 128.02, 127.75, 126.99, 126.57, 125.99, 119.13, 118.82, 83.40, 83.36, 80.79, 80.63.

MALDI TOF-MS (Matrix: Dithranol): Calcd: 1025.41 ($[M+H]^+$), Found: 1025.63.

APCI-MS (negative): Calcd: 1023.40 ($[M-H]^-$), Found: 1023.45.

Pre-G1-2 (14).

A 200 ml flask (three-necked round-bottom flask) containing 4,4'-Bis(3,4-bis(4-ethynylphenyl) -2,5-diphenyl-phenyl)-diphenylmethane (**13**) (0.82 g, 0.80 mmol) and 2,3,4,5-tetraphenyl cyclopenta-2,4 -dien-1-one (1.320 g, 3.435 mmol) was degassed and flushed with argon gas. 50 ml of xylene (anhydrate grade, *o*-, *m*-, *p*-substituted mixture) was added by using a syringe. The mixture was refluxed at 160 ºC for two nights then evaporated. The crude was dissolved in dichloromethane and adsorbed into Celite particle for column and isolated by using a silica gel flush column chromatography [eluting with n-hexane and toluene mixture (gradient from 50:50 to 20:80)]. It afforded 1.557 g (yield 77.6%) of white amorphous. When the product was purified by precipitation with acetone without column chromatography, the yield was enhanced to 90%.

^{1}H NMR (400 MHz, CD_2Cl_2, 25.5 ºC, TMS):

δ [ppm] = 7.45 (s, 2H), 7.41 (s, 2H), 7.37 (s, 2H), 7.14-7.13 (m, 25H), 7.07-7.06 (m, 4H), 7.00 (d, J = 8.0 Hz, 4H), 6.96-6.85 (m, 55H), 6.80-6.72 (m, 24H), 6.66 (d, J = 8.0 Hz, 4H), 6.55 (d, J = 8.0 Hz, 4H), 6.48 (d, J = 8.0 Hz, 4H), 3.79 (s, 2H).

^{13}C NMR (100 MHz, CD_2Cl_2, 26.6 ºC, TMS):

δ [ppm] = 142.30, 142.23, 142.12, 141.72, 141.11, 141.03, 140.99, 140.87, 140.80, 140.64, 140.58, 140.47, 140.44, 140.04, 139.70, 139.64, 139.60, 139.55, 139.48, 139.25, 139.09, 138.86, 138.70, 138.34, 131.92, 131.90, 131.84, 131.49, 131.46, 131.42, 131.34, 130.58, 130.36, 130.34, 130.31, 130.30, 129.00, 128.71, 128.43, 127.99, 127.89, 127.25, 127.21, 127.16, 126.86, 126.59, 125.96, 125.92, 125.64, 41.32.

MALDI TOF-MS (Matrix: Dithranol): Calcd: 2452.05 ($[M+H]^+$), Found: 2451.13.

Anal. Calcd. for $C_{193}H_{132}$: C, 94.57; H, 5.43. Found: C, 94.50; H, 5.40.

G1-2on (15).

Pre-G1-2 (**14**) (1.501 g, 0.612 mmol) and tetrabutylammonium bromide (7.898 g, 24.48 mmol) were dissolved in 150 ml of 1,2-dichloroethane. To the mixture stirred at 0 ºC was added potassium permanganate (3.868 g, 24.48 mmol). Then the mixture was stirred at r.t. for 7 days. The resulting solution was quenched with aqueous sodium hydrogen sulfite. The organic phase was extracted with dichloromethane and washed with brine and then dried with magnesium sulfate. The solution was filtrated and evaporated. The crude was adsorbed into Celite particle for column and isolated by using a silica gel flush column chromatography [eluting with n-hexane and chloroform mixture (gradient from 100:0 to 0:100)]. Then, the product was purified

by a precipitation with methanol to afford 1.394 g (yield 92%) of white powder.

^{1}H NMR (400 MHz, CD_2Cl_2, 25.9 ºC, TMS):

δ [ppm] = 7.52 (d, J = 7.7 Hz, 4H), 7.51 (s, 2H), 7.48 (s, 2H), 7.442 (s, 2H), 7.19-7.14 (m, 30H), 7.09-7.07 (m, 4H), 6.94-6.82 (m, 50H), 6.76-6.73 (m, 24H), 6.67 (d, J = 8.0 Hz, 4H), 6.55 (d, J = 8.0 Hz, 4H), 6.48 (d, J = 8.0 Hz, 4H).

^{13}C NMR (100 MHz, CD_2Cl_2, 27.0 ºC, TMS):

δ [ppm] = 196.17, 146.12, 141.79, 141.75, 141.59, 141.43, 141.43, 141.02, 140.75, 140.73, 140.40, 140.35, 140.02, 139.97, 139.74, 139.61, 139.44, 139.33, 139.27, 139.22, 139.10, 139.04, 138.80, 138.44, 135.38, 131.62, 131.52, 131.29, 131.21, 131.16, 131.04, 130.99, 129.43, 128.79, 128.53, 127.63, 127.55, 127.10, 126.87, 126.81, 126.57, 126.35, 126.22, 125.91, 125.55, 125.30.

MALDI TOF-MS (Matrix: Dithranol): Calcd: 2466.03 ($[M+H]^+$), Found: 2466.04.

Anal. Calcd. for $C_{193}H_{130}O$: C, 94.04; H, 5.32. Found: C, 93.86; H, 5.29.

4-Acetamide-4'-(3,4-bis(4-triisopropylsilylethynylphenyl)-2,5-diphenyl-phenyl)-diphenylmethane (16).

A 300 ml flask (three-necked round-bottom flask) containing 4-acetamide-4'-ethynyl-diphenylmethane (**9**) (4.88 g, 19.6 mmol) and 3,4-bis(4-triisopropylsilylethynyl)phenyl-2,5-diphenyl-cyclopenta-2,4-dien-1-one (**5**) (16.4 g, 22.0 mmol) was degassed and flushed with argon gas. 100 ml of xylene (anhydrate grade, *o*-, *m*-, *p*-substituted mixture) was added by using a syringe. The mixture was refluxed at 170 ºC for 26 hours and then stirred at room temperature overnight before it was evaporated to afford brown amorphous. The crude was dissolved in ethyl acetate and adsorbed into Celite particle for column. The product was isolated by using a silica gel flush column chromatography [eluting with n-hexane and ethyl acetate mixture (gradient from 100:0 to 50:50)] and a gel permeation chromatography (chloroform). It afforded 10.86 g (yield 60%) of beige amorphous.

^{1}H NMR (400 MHz, CD_2Cl_2, 25.0 ºC, TMS):

δ [ppm] = 7.49 (s, 1H), 7.45-7.42 (m, 1H), 7.38 (d, J = 8.0 Hz, 2H), 7.18-7.12 (m, 5H), 7.09-6.95 (m, 13H), 6.87-6.82 (m, 4H), 6.78 (d, J = 8.0 Hz, 2H), 3.83 (s, 2H), 2.07 (s, 3H), 1.08 (s, 42H).

^{13}C NMR (100 MHz, CD_2Cl_2, 26.3 ºC, TMS):

δ [ppm] = 168.51, 141.85, 141.35, 141.25, 141.18, 140.77, 140.22, 139.83, 139.71, 138.62, 137.38, 136.73, 132.02, 131.83, 131.75, 131.06, 130.79, 130.33, 130.22, 129.56, 128.46, 128.10, 127.41, 126.84, 126.23, 121.22, 120.94, 120.33, 107.35, 90.95, 90.79, 41.17, 24.60, 18.79, 11.72.

APCI-MS (positive): Calcd: 966.55 ($[M+H]^+$), Found: 966.54.

Anal. Calcd. for $C_{67}H_{75}NOSi_2$: C, 83.26; H, 7.82; N, 1.45. Found: C, 83.16; H, 7.60; N, 1.51.

4-Acetamide-4'-(3,4-bis(4-ethynylphenyl)-2,5-diphenyl-phenyl)-diphenylmethane (17).

A 300 ml flask (three-necked round-bottom flask) containing 4-acetamide-4'-(3,4-bis(4-triisopropylsilyl ethynylphenyl)-2,5-diphenyl-phenyl)-diphenylmethane (**16**) (10.86 g, 11.24 mmol) was degassed and flushed with argon gas. 100 ml of tetrahydrofuran was added. To the stirred mixture was added 36.0 ml of 1.0M tetrabutylammonium fluoride solution in tetrahydrofuran (36.0 mmol) by using a syringe. After stirring for 160 min, the resulting solution was quenched with K_2CO_3 aq. The organic phase was extracted with ethyl acetate. The organic phase was washed with brine and dried with magnesium sulfate. The solution was filtrated and

evaporated to quantitatively afford 7.34 g of beige amorphous. The product can be purified by gel permeation chromatography (chloroform) and reprecipitation with n-hexane to be pale beige powder.

^{1}H NMR (400 MHz, CD_2Cl_2, 25.1 ºC, TMS):

δ [ppm] = 7.51 (s, 1H), 7.43 (s, 1H), 7.40 (d, *J* = 8.0 Hz, 2H), 7.17-7.12 (m, 5H), 7.07-7.05 (m, 6H), 7.02-6.96 (m, 7H), 6.86-6.81 (m, 4H), 6.76 (d, *J* = 8.0 Hz, 2H), 3.85 (s, 2H), 3.02 (s, 1H), 3.00 (s, 1H), 2.09 (s, 3H).

^{13}C NMR (100 MHz, CD_2Cl_2, 26.0 ºC, TMS):

δ [ppm] = 168.52, 141.69, 141.65, 141.41, 141.26, 141.20, 141.16, 140.12, 139.94, 139.66, 138.55, 137.34, 136.82, 132.02, 131.93, 131.84, 131.19, 130.90, 130.36, 130.26, 129.58, 128.50, 128.12, 127.44, 126.90, 126.27, 120.31, 119.81, 119.50, 83.84, 77.42, 77.28, 41.19, 24.65.

MALDI TOF-MS (Matrix: Dithranol): Calcd: 654.28 ($[M+H]^+$), Found: 654.35.

APCI-MS (positive): Calcd: 654.28 ($[M+H]^+$), Found: 654.12.

Pre-G1-2-half-NHAc (18).

A 300 ml flask (three-necked round-bottom flask) containing 4-acetamide-4'-(3,4-bis(4-ethynyl phenyl)-2,5-diphenyl-phenyl)-diphenylmethane (**17**) (7.34 g, 11.23 mmol) and 2,3,4,5-tetraphenyl cyclopenta-2,4-dien-1-one (14.00 g, 36.41 mmol) was degassed and flushed with argon gas. 200 ml of xylene (anhydrate grade, *o*-, *m*-, *p*-substituted mixture) was added by using a syringe. The mixture was refluxed at 170 ºC for two nights then evaporated. The crude was dissolvable in acetone which could be used for the precipitation as “pre-G1-2”. The crude was dissolved in chloroform and adsorbed into Celite particle for column. The product was isolated by using a silica gel flash column chromatography [eluting with n-hexane and toluene mixture (gradient from 50:50 to 0:100) and then eluting with toluene and ethyl acetate mixture (gradient from 100:0 to 0:100)]. It afforded 14.942 g (yield 91%) of orange amorphous.

^{1}H NMR (400 MHz, $CDCl_3$, 25.8 ºC, TMS):

δ [ppm] = 7.48 (d, *J* = 3.4 Hz, 2H), 7.44 (s, 1H), 7.37 (d, *J* = 8.2 Hz, 2H), 7.14 (s, 13H), 7.07-7.05 (m, 5H), 7.00 (d, *J* = 8.0 Hz, 2H), 6.93-6.82 (m, 27H), 6.76-6.71 (m, 12H), 6.66 (d, *J* = 8.0 Hz, 2H), 6.53 (d, *J* = 8.0 Hz, 2H), 6.47 (d, *J* = 8.0 Hz, 2H), 3.85 (s, 2H), 2.15 (s, 3H).

^{13}C NMR (100 MHz, $CDCl_3$, 27.1 ºC, TMS):

δ [ppm] = 168.07, 141.81, 141.72, 141.14, 140.76, 140.73, 140.71, 140.60, 140.48, 140.44, 140.05, 140.01, 139.98, 139.62, 139.30, 139.21, 139.17, 139.10, 139.04, 138.83, 138.62, 138.25, 138.16, 137.78, 137.22, 135.86, 131.71, 131.63, 131.53, 131.51, 131.23, 131.14, 131.06, 130.05, 130.02, 129.95, 129.43, 128.73, 128.45, 128.14, 127.54, 126.86, 126.80, 126.56, 126.20, 125.57, 125.28, 120.03, 40.88, 24.56.

MALDI TOF-MS (Matrix: Dithranol): Calcd: 1367.60 ($[M+H]^+$), Found: 1368.02.

Anal. Calcd. for $C_{105}H_{75}NO$: C,92.27; H, 5.53; N, 1.02. Found: C, 91.96; H, 5.28; N, 1.07.

Pre-G1-2-half-NH (19).

A 200 ml flask (three-necked round-bottom flask) containing pre-G1-2-half-NHAc (**18**) (3.630 g, 2.654 mmol) was degassed and flushed with argon gas. 80 ml of tetrahydrofuran and 5 ml of methanol were added. To the stirred mixture was added 10.0 ml of boron trifluoride: diethyl ether complex (; BF_3:Et_2O) (11.3 g, 79.6

mmol) by using a hole pipette. The mixture was refluxed at 80 ºC for 10 days. The resulting solution was quenched with water and neutralized with aqueous potassium carbonate. The organic phase was extracted with ethyl acetate and washed with sodium thiosulfate several times. The organic phase was washed brine and dried with magnesium sulfate. The solution was filtered and evaporated. The crude was dissolved in ethyl acetate and adsorbed into silica gel. The product was isolated by using a silica gel flash column chromatography [eluting with n-hexane and ethyl acetate mixture (gradient from 100:0 to 50:50)]. It afforded 3.485 g (yield 99%) of beige solid.

^{1}H NMR (400 MHz, $CDCl_3$, 25.5 ºC, TMS):

δ [ppm] = 7.49 (d, J = 3.9 Hz, 2H), 7.44 (s, 1H), 7.14 (s, 13H), 7.07-7.05 (m, 2H), 6.99 (d, J = 8.0 Hz, 2H), 6.94-6.82 (m, 29H), 6.76-6.71 (m, 12H), 6.65 (d, J = 8.0 Hz, 2H), 6.60 (d, J = 8.0 Hz, 2H), 6.53 (d, J = 8.0 Hz, 2H), 6.47 (d, 2H), 3.780 (s, 2H), 3.54 (br, 2H).

^{13}C NMR (100 MHz, $CDCl_3$, 26.8 ºC, TMS):

δ [ppm] = 144.42, 141.84, 141.80, 141.72, 141.12, 140.73, 140.70, 140.48, 140.44, 140.11, 140.05, 140.01, 139.98, 139.69, 139.32, 139.28, 139.20, 139.16, 139.10, 139.04, 138.60, 138.56, 138.22, 138.20, 137.80, 131.73, 131.63, 131.53, 131.51, 131.23, 131.20, 131.15, 131.07, 130.06, 129.95, 129.92, 129.75, 128.72, 128.44, 128.05, 127.54, 126.86, 126.80, 126.56, 126.20, 125.53, 125.28, 115.25, 40.61.

MALDI TOF-MS (Matrix: Dithranol): Calcd: 1325.58 ($[M+H]^+$), Found: 1325.85.

Anal. Calcd. for $C_{103}H_{73}N$: C,93.39; H, 5.55; N, 1.06. Found: C, 93.31; H, 5.27; N, 1.12.

Pre-G1-2-half-2-1 (20).

A 100 ml flask (three-necked round-bottom flask) containing pre-G1-2-half-NH (**19**) (2.727 g, 2.058 mmol) and G1-1on (**11**) (2.030 g, 2.152 mmol) and 4-diazabicyclo[2.2.2]octane (DABCO) (1.645 g, 14.66 mmol) was degassed and flushed with argon gas. The mixture was dissolved in 60 ml of chlorobenzene at 100 ºC. Titanium chloride (0.400 ml, 3.65 mmol) was dissolved in 5 ml of chlorobenzene, and then dropwise added to the stirred mixture by using a dropping funnel. The reaction mixture was stirred at 125 ºC for 6 hours in an argon atmosphere and then stirred at room temperature in air overnight. The resulting solution was filtered through a pad of Celite and silica gel. The filtrate was concentrated. The product was isolated by a gel permeation chromatography (chloroform) and precipitation with methanol. It afforded 3.545 g (yield 76%) of yellow powder.

^{1}H NMR (400 MHz, CD_2Cl_2, 26.0 ºC, TMS):

δ [ppm] = 7.54 (s, 1H), 7.49 (s, 1H), 7.45 (s, 1H), 7.42 (d, J = 8.3 Hz, 2H), 7.41 (s, 1H), 7.37 (s, 1H), 7.19 (s, 1H), 7.16-7.11 (m, 24H), 7.06-6.97 (m, 10H), 6.94-6.84 (m, 53H), 6.82-6.77 (m, 11H), 6.74-6.72 (m, 5H), 6.65 (d, J = 8.0 Hz, 2H), 6.55 (d, J = 8.0 Hz, 4H), 6.48 (d, J = 8.0 Hz, 2H), 3.81 (s, 2H).

MALDI TOF-MS (Matrix: Dithranol): Calcd: 2249.96 ($[M+H]^+$), Found: 2250.29.

Anal. Calcd. for $C_{176}H_{121}N$: C, 93.96; H, 5.42; N, 0.62. Found: C, 93.93; H, 5.41; N, 0.68.

G1-2-half-2-1on (21).

Pre-G1-2-half-2-1 (**20**) (3.500 g, 1.556 mmol) and tetrabutylammonium bromide (20.04 g, 62.22 mmol) were dissolved in 200 ml of 1,2-dichloroethane. To the mixture stirred at 0 ºC was added potassium permanganate (9.83 g, 62.2 mmol). Then the mixture was stirred at r.t. for 5 days. The resulting solution was quenched with aqueous sodium hydrogen sulfite and filtered through a pad of Celite. The organic phase was extracted with 10% trimethylamine-attached chloroform and washed with brine and then dried with magnesium sulfate. The solution was filtered and evaporated. The product was purified by precipitation with methanol to afford 3.446 g (yield 97.8%) of yellow powder.

^{1}H NMR (400 MHz, CD_2Cl_2, 26.3 ºC, TMS):

δ [ppm] = 7.56-7.42 (m, 9H), 7.42 (s, 1H), 7.38 (s, 1H), 7.22-7.08 (m, 29H), 7.03-6.85 (m, 52H), 6.79-6.74 (m, 19H), 6.67 (t, J = 7.5 Hz, 4H), 6.57 (d, J = 7.3 Hz, 2H), 6.51 (d, J = 7.2 Hz, 2H).

^{13}C NMR (100 MHz, CD_2Cl_2, 27.7 ºC, TMS):

δ [ppm] = 195.41, 168.54, 155.94, 146.19, 145.16, 142.94, 142.40, 142.30, 142.14, 142.10, 141.90, 141.34, 141.12, 141.01, 140.83, 140.76, 140.72, 140.62, 140.45, 140.33, 140.16, 139.82, 139.77, 139.68, 139.64, 139.56, 139.49, 139.38, 139.02, 138.45, 138.18, 137.31, 136.32, 132.47, 131.99, 131.92, 131.90, 131.87, 131.81, 131.59, 131.44, 131.37, 131.31, 131.09, 130.29, 130.12, 129.91, 129.47, 129.08, 128.78, 128.05, 127.95, 127.90, 127.44, 127.24, 127.16, 126.96, 126.87, 126.73, 126.60, 126.28, 126.04, 125.93, 125.78, 125.65, 120.74.

MALDI TOF-MS (Matrix: Dithranol): Calcd: 2263.94 ($[M+H]^+$), Found: 2264.55.

Anal. Calcd. for $C_{176}H_{119}NO$: C, 93.38; H, 5.30; N, 0.63. Found: C, 92.98; H, 5.36; N, 0.68.

Pre-G2-2-half-NH (22).

A 200 ml flask (three-necked round-bottom flask) containing G1-2on (**15**) (2.500 g, 1.014 mmol) and 4,4'-diamino-diphenylmethane (4.020 g, 20.27 mmol) and 4-diazabicyclo[2.2.2]octane (DABCO) (1.640 g, 14.62 mmol) was degassed and flushed with argon gas. The mixture was dissolved in 100 ml of chlorobenzene at 80 ºC. Titanium chloride (0.400 ml, 3.64 mmol) was dissolved in 8 ml of chlorobenzene, and then dropwise added to the stirred mixture by using a dropping funnel. The reaction mixture was stirred at 125 ºC for 21 hours in an argon atmosphere and then stirred at room temperature for 3.5 hrs in air. The resulting solution was filtered through a pad of Celite and silica gel. The filtrate was concentrated. The product was isolated by a gel permeation chromatography (chloroform) and precipitation with methanol to afford 2.153 g (yield 80%) of beige solid.

^{1}H NMR (400 MHz, $CDCl_3$, 25.3 ºC, TMS):

δ [ppm] = 7.51-7.41 (m, 8H), 7.14-7.08 (m, 32H), 6.96-6.72 (m, 80H), 6.68-6.63 (m, 6H), 6.57-6.53 (m, 8H), 6.49-6.47 (m, 4H), 3.79 (s, 2H), 3.46 (br, 2H).

^{13}C NMR (100 MHz, $CDCl_3$, 26.4 ºC, TMS):

δ [ppm] = 167.48, 149.09, 144.38, 144.11, 142.01, 141.78, 141.73, 141.69, 141.38, 141.08, 140.91, 140.80, 140.74, 140.41, 140.29, 140.07, 140.01, 139.78, 139.72, 139.43, 139.24, 139.09, 139.02, 138.71, 138.35, 137.98, 137.94, 137.70, 137.64, 137.61, 136.46, 134.04, 131.62, 131.51, 131.16, 131.07, 131.01, 130.03, 129.94, 129.67, 129.46, 129.33, 128.96, 128.90, 128.75, 128.62, 128.49, 127.54, 127.06, 126.86, 126.80, 126.56, 126.27, 126.21, 125.82, 125.55, 125.29, 121.27, 115.23, 40.44.

MALDI TOF-MS (Matrix: Dithranol): Calcd: 2646.13 ($[M+H]^+$), Found: 2646.33.

Anal. Calcd. for $C_{206}H_{142}N_2$: C, 93.53; H, 5.41; N, 1.06. Found: C, 93.28; H, 5.42; N, 1.18.

Pre-G2-2-quarter-3-1 (23).

A 200 ml flask (three-necked round-bottom flask) containing G1-2-half-2-1on (**21**) (1.815 g, 0.8017 mmol) and pre-G2-2-half-NH (**22**) (2.100 g, 0.7938 mmol) and 4-diazabicyclo[2.2.2]octane (DABCO) (1.640 g, 14.62 mmol) was degassed and flushed with argon gas. The mixture was dissolved in 80 ml of chlorobenzene at 80 ºC. Titanium chloride (0.200 ml, 1.82 mmol) was dissolved in 10 ml of chlorobenzene and dropwise added to the stirred mixture by using a dropping funnel. The reaction mixture was refluxed at 135 ºC for 5 hours in an argon atmosphere. Additionally, titanium chloride (0.200 ml, 1.82 mmol) was dissolved in 5 ml of chlorobenzene and dropwise added to the reaction mixture by using a dropping funnel. The reaction mixture was refluxed at 135 ºC for 15 hrs in an argon atmosphere and then stirred at room temperature for 3 hrs in air. The resulting solution was filtered through a pad of Celite and silica gel. The filtrate was concentrated. The product was isolated by a gel permeation chromatography (chloroform) and precipitation with methanol to afford 3.276 g (yield 84%) of yellow powder.

^{1}H NMR (400 MHz, CD_2Cl_2, 25.5 ºC, TMS):

δ [ppm] = 7.55-7.35 (m, 17H), 7.21-7.02 (m, 62H), 6.98-6.65 (m, 160H), 6.61-6.44 (m, 18H), 3.83 (s, 1H), 3.66 (s, 1H).

^{13}C NMR (100 MHz, CD_2Cl_2, 26.8 ºC, TMS):

δ [ppm] = 168.31, 168.20, 149.61, 144.99, 144.91, 144.69, 142.80, 142.53, 142.30, 142.13, 141.90, 141.60, 141.28, 141.13, 141.02, 140.86, 140.79, 140.76, 140.72, 140.64, 140.47, 140.45, 140.36, 140.22, 139.84, 139.71, 139.67, 139.61, 139.56, 139.49, 139.35, 139.31, 138.97, 138.53, 138.25, 137.65, 136.48, 134.72, 134.48, 134.13, 133.73, 132.03, 131.93, 131.84, 131.71, 131.47, 131.39, 131.33, 130.31, 130.00, 129.82, 129.63, 129.32, 129.24, 129.04, 128.76, 128.04, 128.01, 127.97, 127.90, 127.42, 127.25, 127.22, 127.17, 126.98, 126.87, 126.70, 126.60, 126.24, 126.05, 125.97, 125.93, 125.78, 125.65, 121.66, 121.50, 120.82, 41.01

MALDI TOF-MS (Matrix: Dithranol): Calcd: 4892.06 ($[M+H]^+$), Found: 4892.39.

Anal. Calcd. for $C_{382}H_{259}N_3$: C, 93.80; H, 5.34; N, 0.86. Found: C, 93.56; H, 5.33; N, 0.92.

G2-2-quarter-3-1on (; also named as "4Non") (24).

Pre-G2-2-quarter-3-1 (**23**) (3.275 g, 0.6695 mmol) and tetrabutylammonium bromide (8.640 g, 26.78 mmol) were dissolved in 210 ml of 1,2-dichloroethane. To the mixture stirred at 0 ºC was added potassium permanganate (4.240 g, 26.78 mmol). Then the mixture was stirred at r.t. for 6 days. The resulting solution was quenched with aqueous sodium hydrogen sulfite. The organic phase was extracted with 10% trimethylamine-attached chloroform and washed with brine and then dried with magnesium sulfate. The solution was filtrated and evaporated. The product was isolated by a gel permeation chromatography (chloroform) and precipitation with methanol to afford 3.190 g (yield 97%) of yellow powder.

^{1}H NMR (400 MHz, CD_2Cl_2, 26.1 ºC, TMS, drop of triethylamine was added as a stabilizer):

δ [ppm] = 7.58-7.35 (m, 21H), 7.22-7.03 (m, 61H), 6.98-6.63 (m, 162H), 6.58-6.45 (m, 13H).

^{13}C NMR (100 MHz, CD_2Cl_2, 26.9 ºC, TMS, drop of triethylamine was added as a stabilizer:

δ [ppm] = 194.85, 168.45, 168.33, 168.27, 155.89, 155.62, 154.52, 145.18, 145.03, 142.94, 142.87, 142.39, 142.28, 142.12, 142.09, 141.93, 141.60, 141.33, 141.12, 141.00, 140.83, 140.77, 140.73, 140.62, 140.58, 140.45, 140.37, 140.18, 140.06, 139.84, 139.71, 139.65, 139.60, 139.54, 139.48, 139.31, 138.97, 138.46, 138.20, 137.50, 137.32, 134.05, 133.77, 132.96, 132.76, 131.91, 131.82, 131.45, 131.36, 131.30, 131.10, 130.49, 130.30, 130.12, 129.91, 129.03, 128.75, 127.98, 127.89, 127.39, 127.24, 127.20, 127.16, 126.97, 126.86, 126.69, 126.58, 126.21, 126.03, 125.92, 125.78, 125.64, 120.89, 120.83, 120.66

MALDI TOF-MS (Matrix: Dithranol): Calcd: 4906.04 ($[M+H]^+$), Found: 4906.49.

Anal. Calcd. for $C_{382}H_{257}N_3O$: C, 93.54; H, 5.28; N, 0.86. Found: C, 93.40; H, 5.34; N, 0.97.

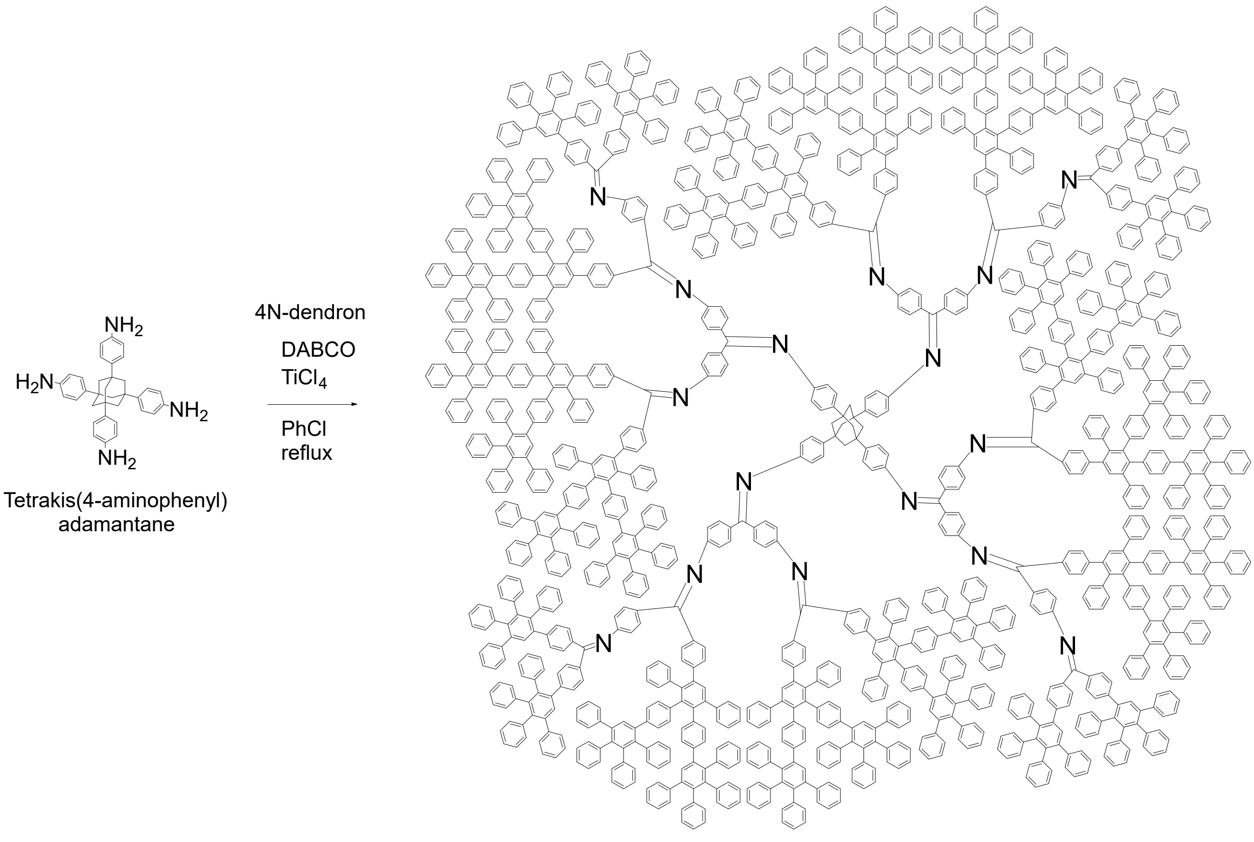

25 (= 16N-dendrimer)

Scheme S5. Synthesis of adamantane-core polyphenylene jacketed phenylazomethine dendrimer (PPDPA16).

PPDPA16 (16N dendrimer) (25).

A 100 ml flask (three-necked round-bottom flask) containing 1,3,5,7-Tetrakis(4-aminophenyl)adamantane (20.4 mg, 40.7 μmol) and 4Non (**24**) (1.002 g, 204.2 μmol) and 4-diazabicyclo[2.2.2]octane (DABCO) (1.23 g, 11.0 mmol) was degassed and flushed with argon gas. The mixture was dissolved in 35 ml of chlorobenzene at 100 ºC. Titanium chloride (0.100 ml, 0.912 mmol) was dissolved in 5 ml of chlorobenzene and dropwise added to the stirred mixture by using a dropping funnel. The reaction mixture was refluxed at 130 ºC for 27 hours in an argon atmosphere. Additionally, titanium chloride (0.100 ml, 0.912 mmol) was dissolved in 4 ml of chlorobenzene and dropwise added to the reaction mixture by using a dropping funnel. The reaction mixture was refluxed at 130 ºC for 18 hrs in an argon atmosphere and then stirred at room temperature for 5 hrs in air. The resulting solution was filtered through a pad of Celite and silica gel. The filtrate was concentrated. The product was isolated by a gel permeation chromatography (chloroform) and precipitation with methanol to afford 153.9 mg (yield 19%) of yellow powder as PPDPA16 (**25**) and also 183.3 mg (yield 30%) of yellow powder as 4Non tri-substituent derivative.

^{1}H NMR (400 MHz, CD_2Cl_2, 27.1 ºC, TMS):

δ [ppm] = 7.55-7.25 (m, 80H), 7.21-6.63 (m, 882H), 6.52-6.47 (m, 82H), 1.79 (br, 12H).

^{13}C NMR (100 MHz, CD_2Cl_2, 26.0 ºC, TMS):

δ [ppm] = 168.03, 166.78, 154.16, 153.73, 152.11, 151.89, 149.58, 144.81, 142.63, 142.24, 142.08, 142.03, 141.84, 141.56, 141.10, 140.98, 140.83, 140.75, 140.60, 140.42, 140.21, 140.15, 139.82, 139.59, 139.53, 139.46, 139.36, 139.24, 138.95, 138.46, 138.22, 137.52, 135.14, 134.29, 134.03, 133.81, 131.89, 131.46, 131.35, 131.10, 130.27, 129.02, 128.76, 127.88, 127.39, 127.15, 126.96, 126.85, 126.66, 126.57, 126.22, 125.91, 125.62, 121.29, 120.86, 47.62, 39.07.

MALDI TOF-MS (Matrix: Dithranol): Calcd: 20049.37 ($[M+H]^+$), Found: 20049.37.

Anal. Calcd. for $C_{1562}H_{1056}N_{16}$: C, 93.57; H, 5.31; N, 1.22. Found: C, 93.30; H, 5.25; N, 1.23.

4. UV-vis titration

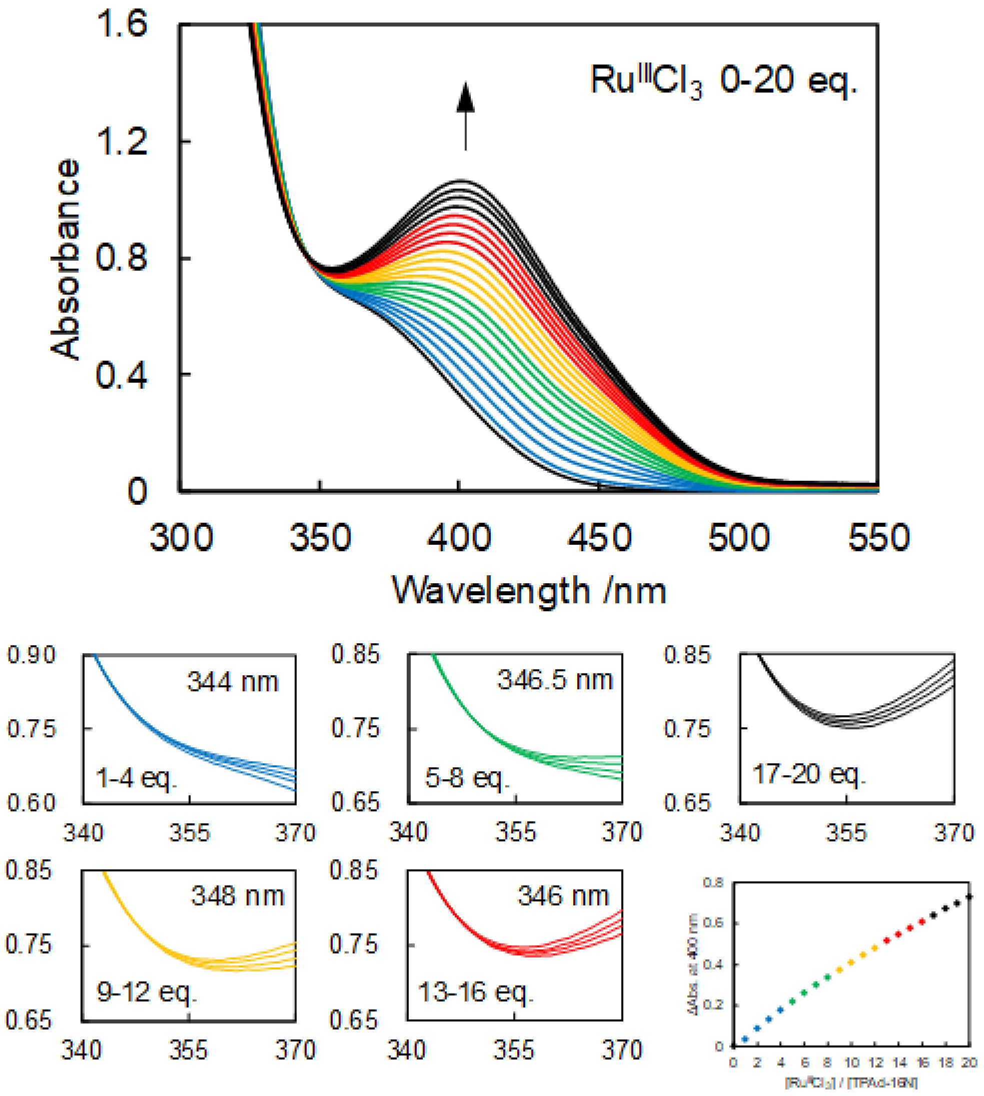

Figure S3. Accumulation of Ru to PPDPA16. (top) Change in UV-vis absorption spectra of PPDPA16 in DCM upon the addition of Ru^{III}Cl$_3$ in MeCN up to 20 equiv. ([PPDPA16] = ca. 5 µM, 20 °C). (down) Enlarged views during complexation with Ru^{III}Cl$_3$ in respective positions 0–4, 5–8, 9–12, 13–16, and 17-20 equiv and the ΔAds plot during the addition of Ru^{III}Cl$_3$.

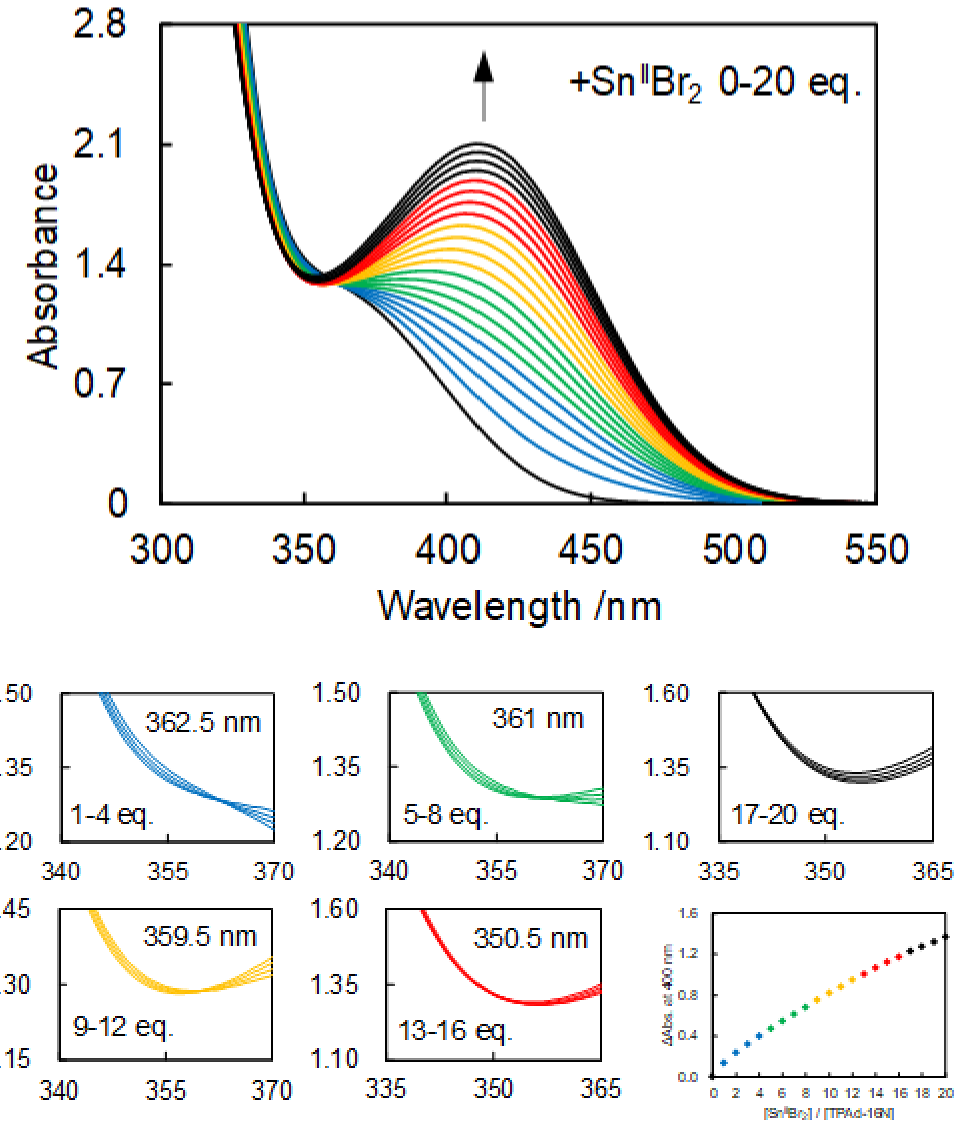

Figure S4 Accumulation of Sn to PPDPA16. (top) Change in UV-vis absorption spectra of PPDPA16 in DCM/MeCN = 2/1 upon the addition of $Sn^{II}Br_2$ in MeCN up to 20 equiv. ([PPDPA16] = ca. 10 μM, 20 °C). (down) Enlarged views during complexation with $Sn^{II}Br_2$ in respective positions 0–4, 5–8, 9–12, 13–16, and 17-20 equiv and the ΔAds plot during the addition of $Sn^{II}Br_2$.

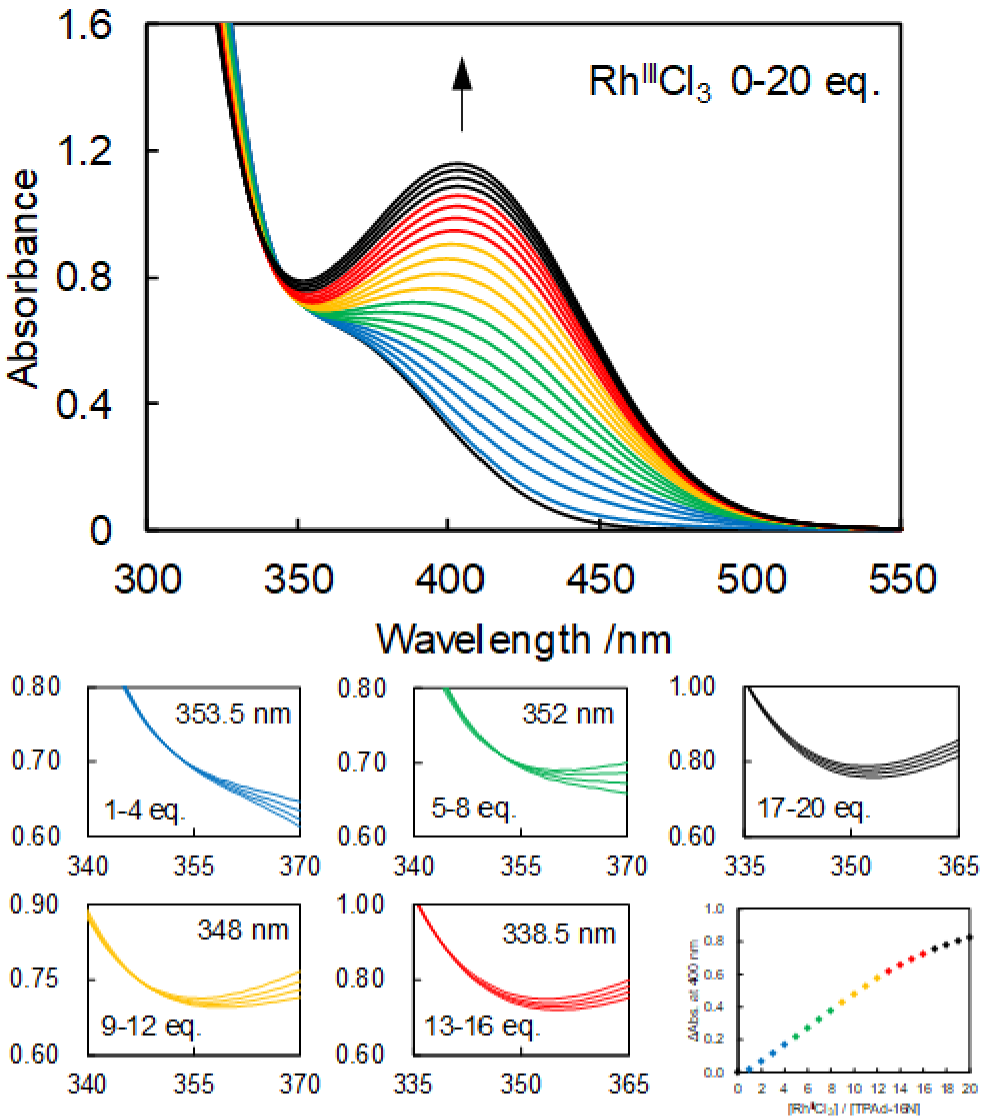

Figure S5 Accumulation of Rh to PPDPA16. (top) Change in UV-vis absorption spectra of PPDPA16 in DCM upon the addition of Rh^{III}Cl$_3$ in MeCN/MeOH =250/1 up to 20 equiv. ([PPDPA16] = ca. 5 μM, 20 °C). (down) Enlarged views during complexation with Rh^{III}Cl$_3$ in respective positions 0–4, 5–8, 9–12, 13–16, and 17-20 equiv and the ΔAds plot during the addition of Rh^{III}Cl$_3$.

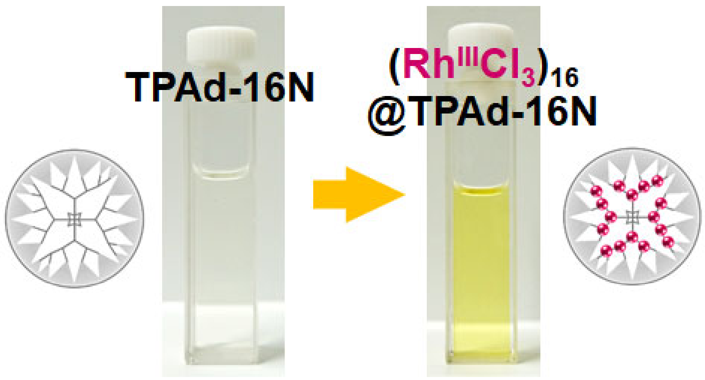

Figure S6. Photo of the PPDPA**16** solution before and after the addition of 16 equivalent of Rh^{III}Cl$_3$.

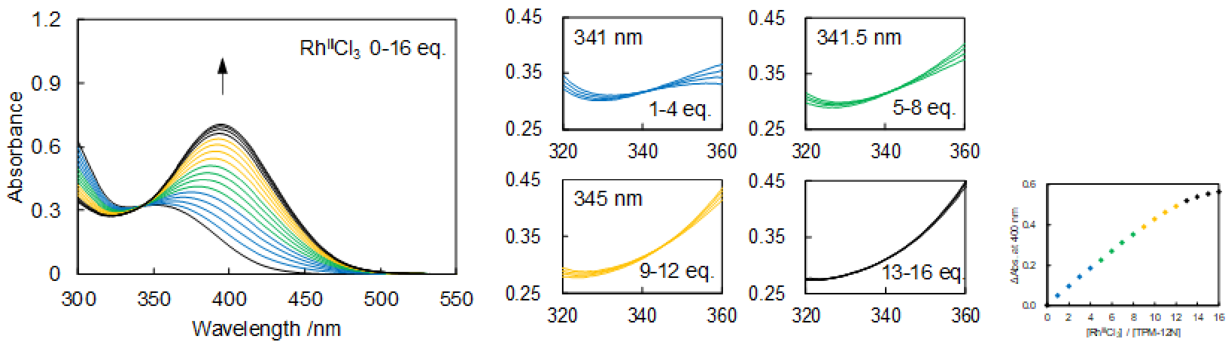

Figure S7 Accumulation of Rh to DPA12. (left) Change in UV-vis absorption spectra of DPA12 in DCM upon the addition of Rh^{III}Cl$_3$ in MeCN/MeOH =250/1 up to 16 equiv. ([DPA12] = ca. 5 μM, 20 °C). (right) Enlarged views during complexation with Rh^{III}Cl$_3$ in respective positions 0–4, 5–8, 9–12, and 13–16 equiv and the ΔAds plot during the addition of Rh^{III}Cl$_3$.

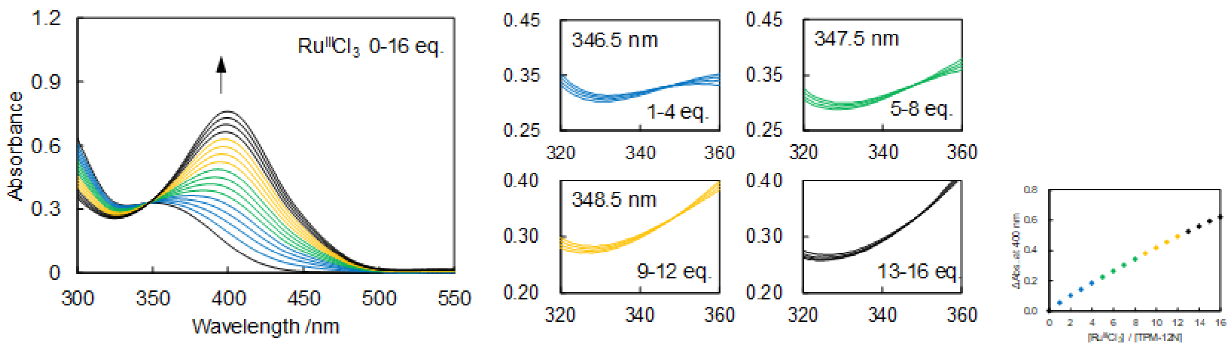

Figure S8 Accumulation of Ru to DPA12. (left) Change in UV-vis absorption spectra of DPA12 in DCM upon the addition of Ru^{III}Cl$_3$ in MeCN up to 16 equiv. ([DPA12] = ca. 5 μM, 20 °C). (right) Enlarged views during complexation with Ru^{III}Cl$_3$ in respective positions 0–4, 5–8, 9–12, and 13–16 equiv and the ΔAds plot during the addition of Ru^{III}Cl$_3$.

5. XPS Spectra

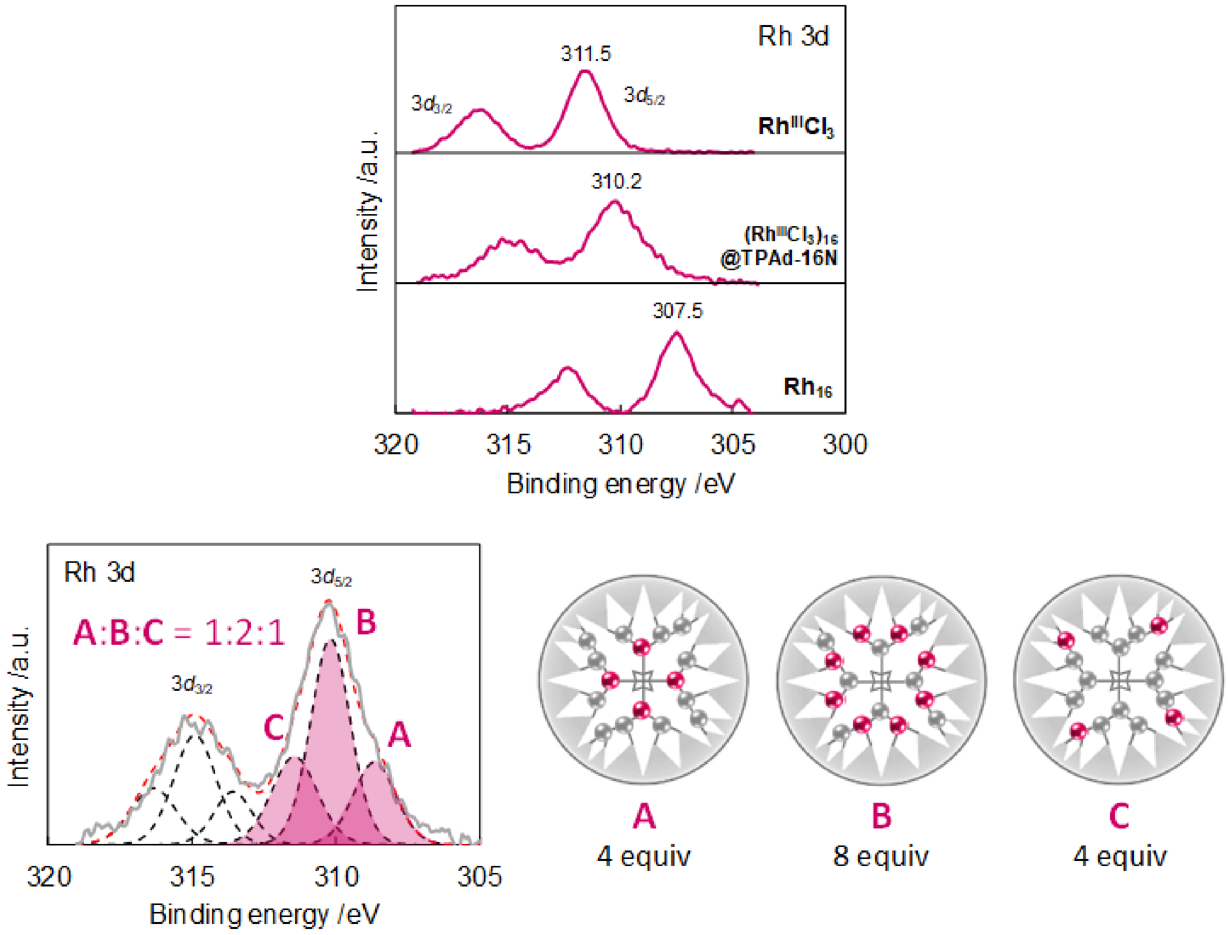

Figure S9 XPS spectra of $Rh^{III}Cl_3$, $(Rh^{III}Cl_3)_{16}$@PPDPA16, and synthesized Rh cluster.

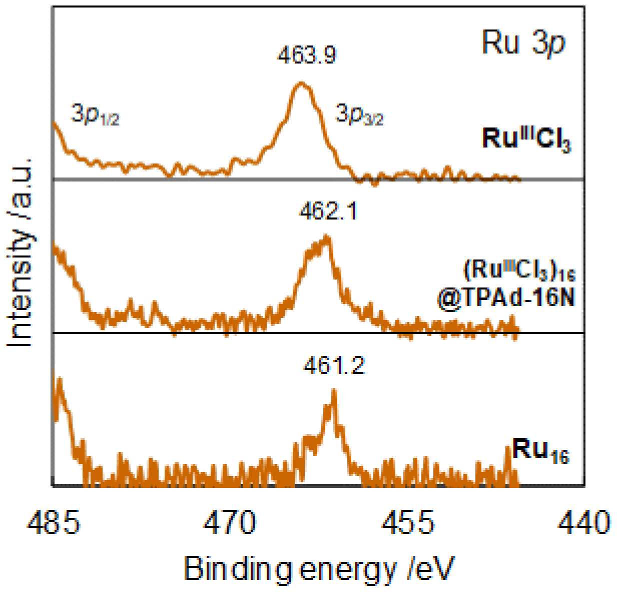

Figure S10 XPS spectra of $Ru^{III}Cl_3$, $(Ru^{III}Cl_3)_{16}$@PPDPA16, and synthesized Ru cluster.

6. STEM Images

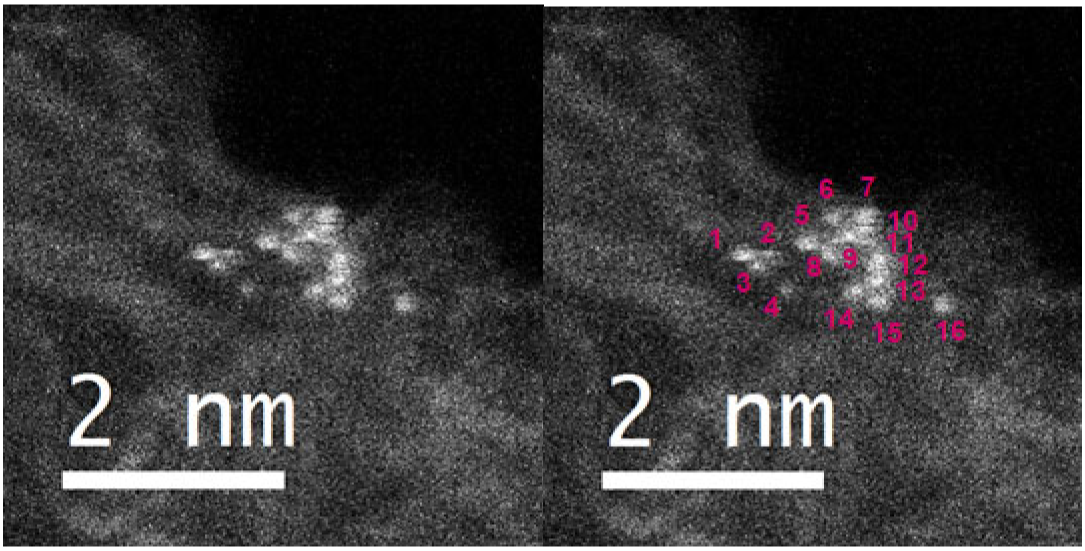

Figure S11 STEM image of Rh nanoparticle after disassembly. The numbers are the count of atoms

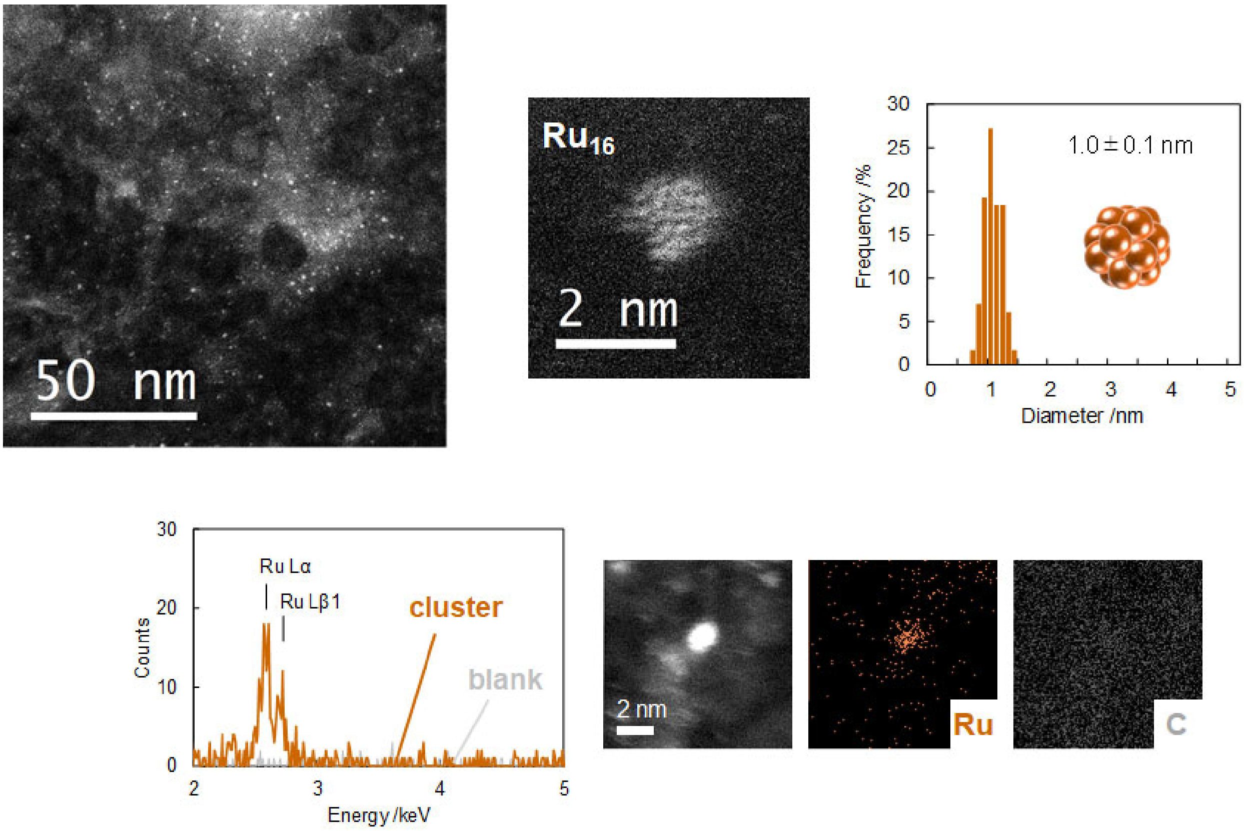

Figure S12. STEM image, histogram, and EDX analysis of the synthesized Ru nanoparticle.

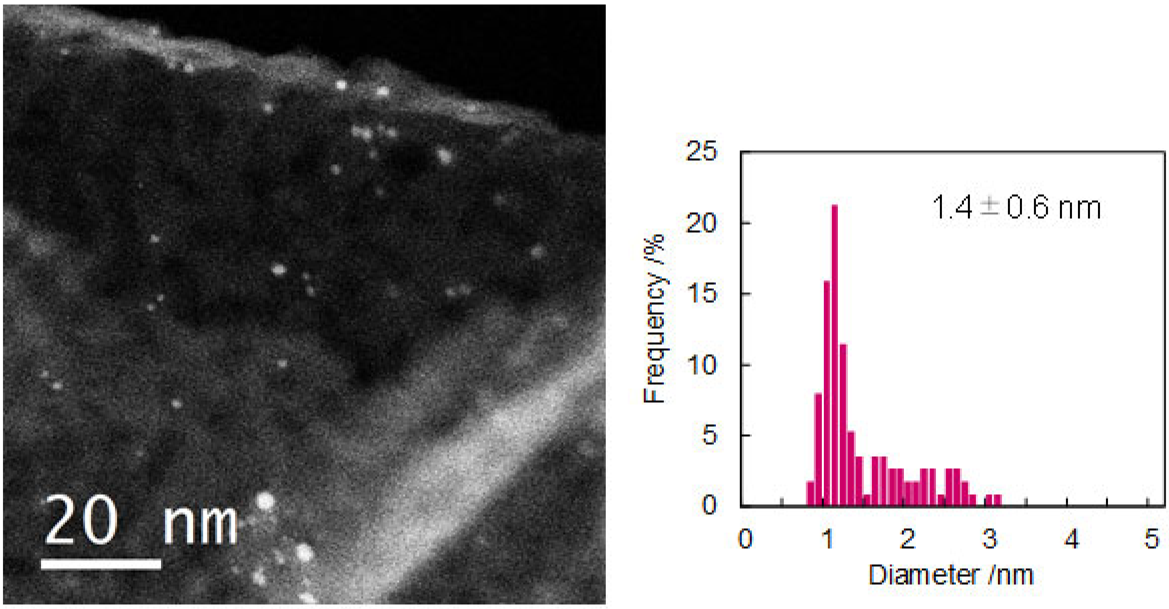

Figure S13 STEM image, and histogram of Rh nanoparticles synthesized with DPA12 with 12 equiv. $RhCl_3$.

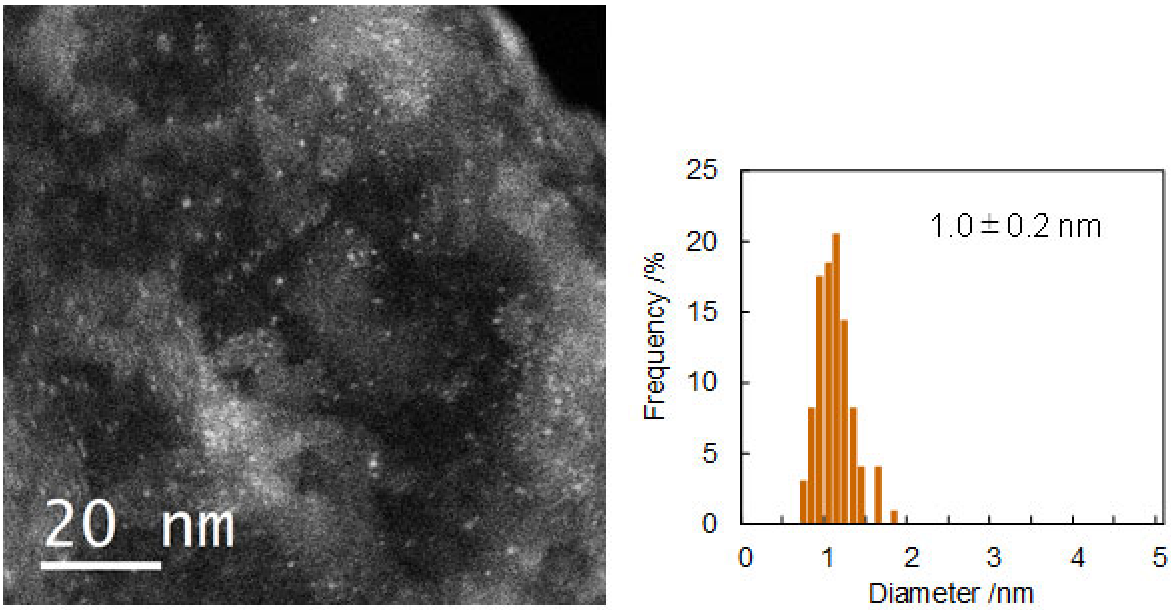

Figure S14 STEM image, and histogram of Ru nanoparticles synthesized with DPA12 with 12 equiv. $RuCl_3$.

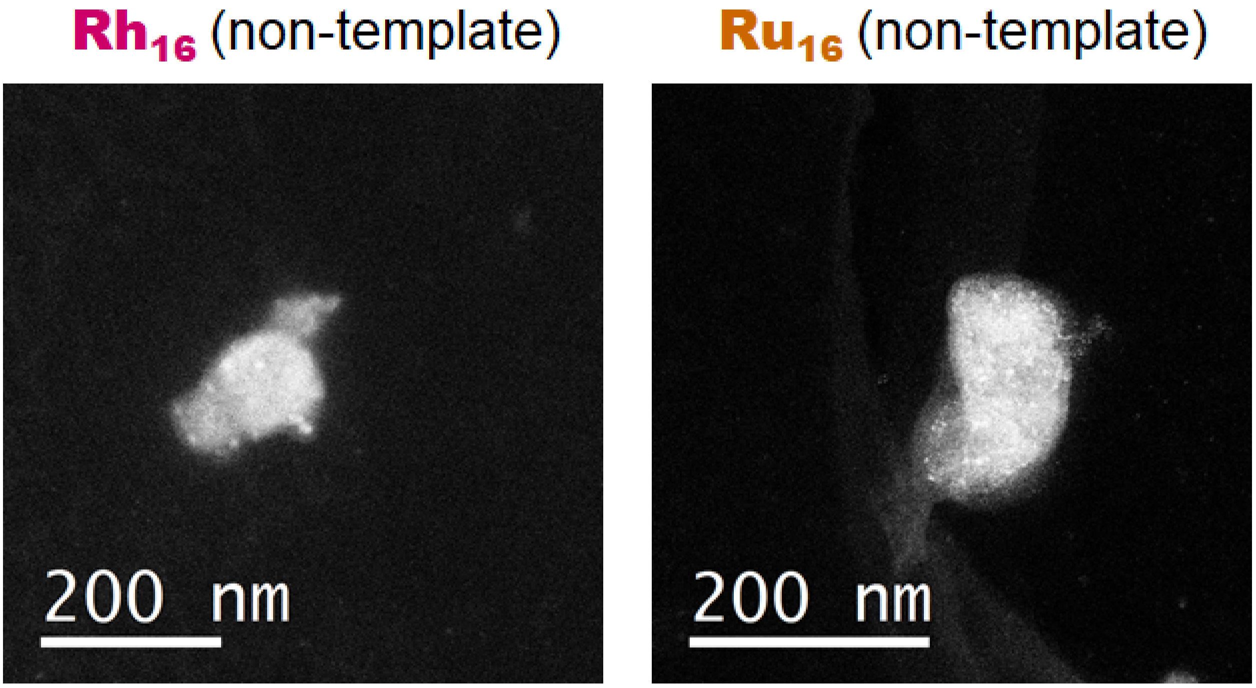

Figure S15 STEM image of Rh and Ru nanoparticles synthesized without any template dendrimer.

4,4'-Diiododiphenylmethane 1H (CDCl3, 400MHz, 302.5K)

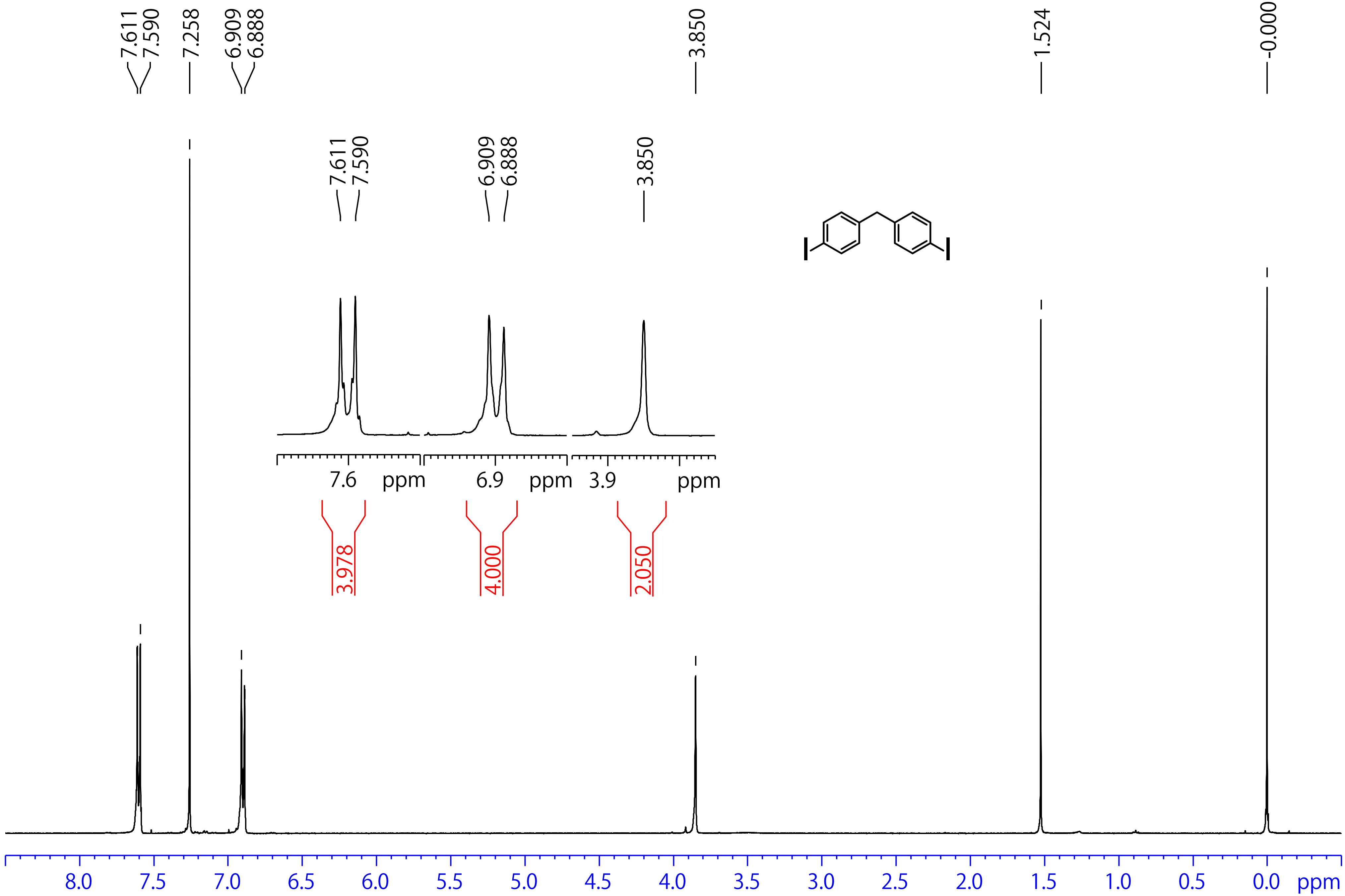

Fig C1. 1H-NMR spectra of compound 1.

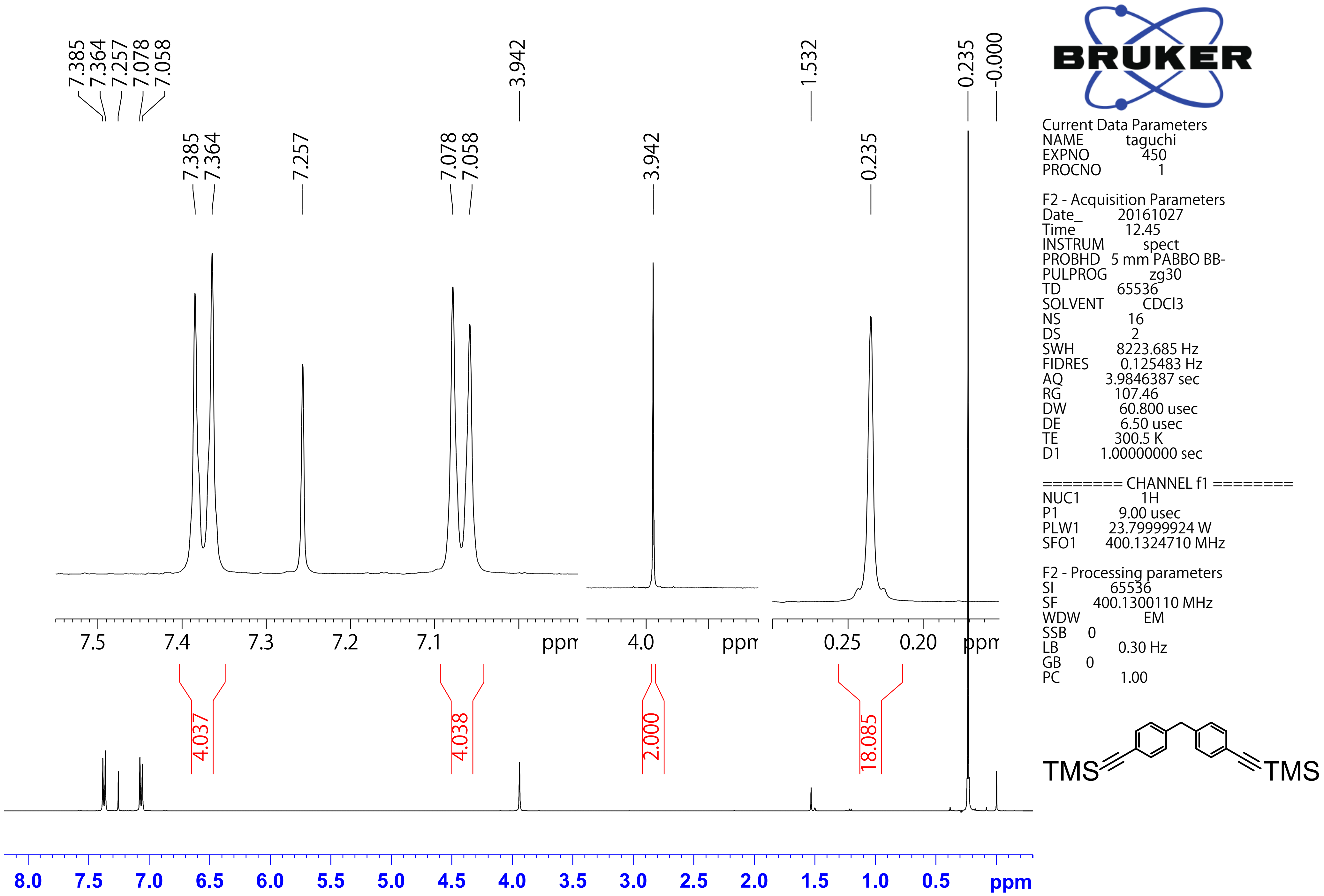

Fig C2. 1H-NMR spectra of compound 2.

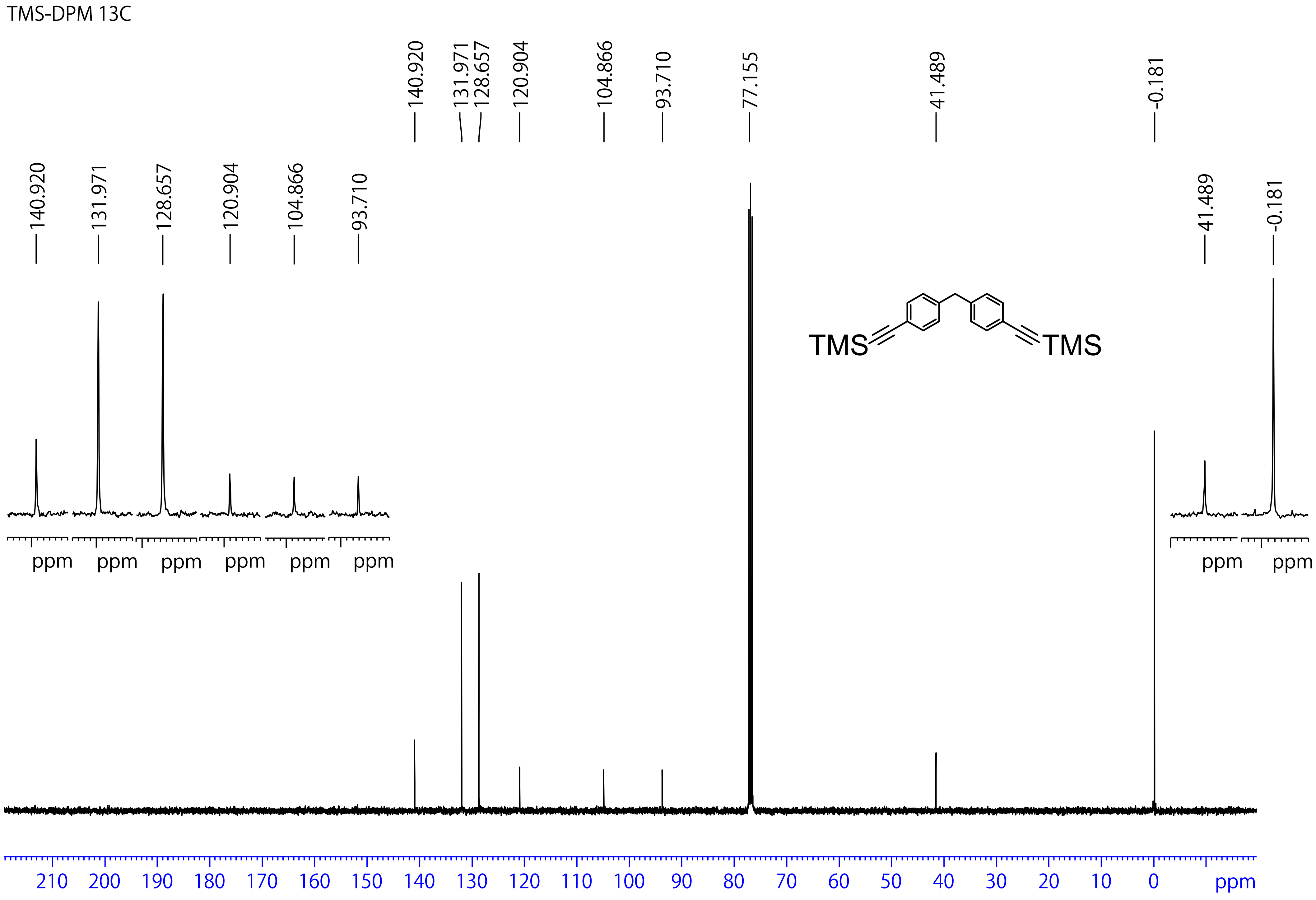

Fig C3. 13C-NMR spectra of compound 2.

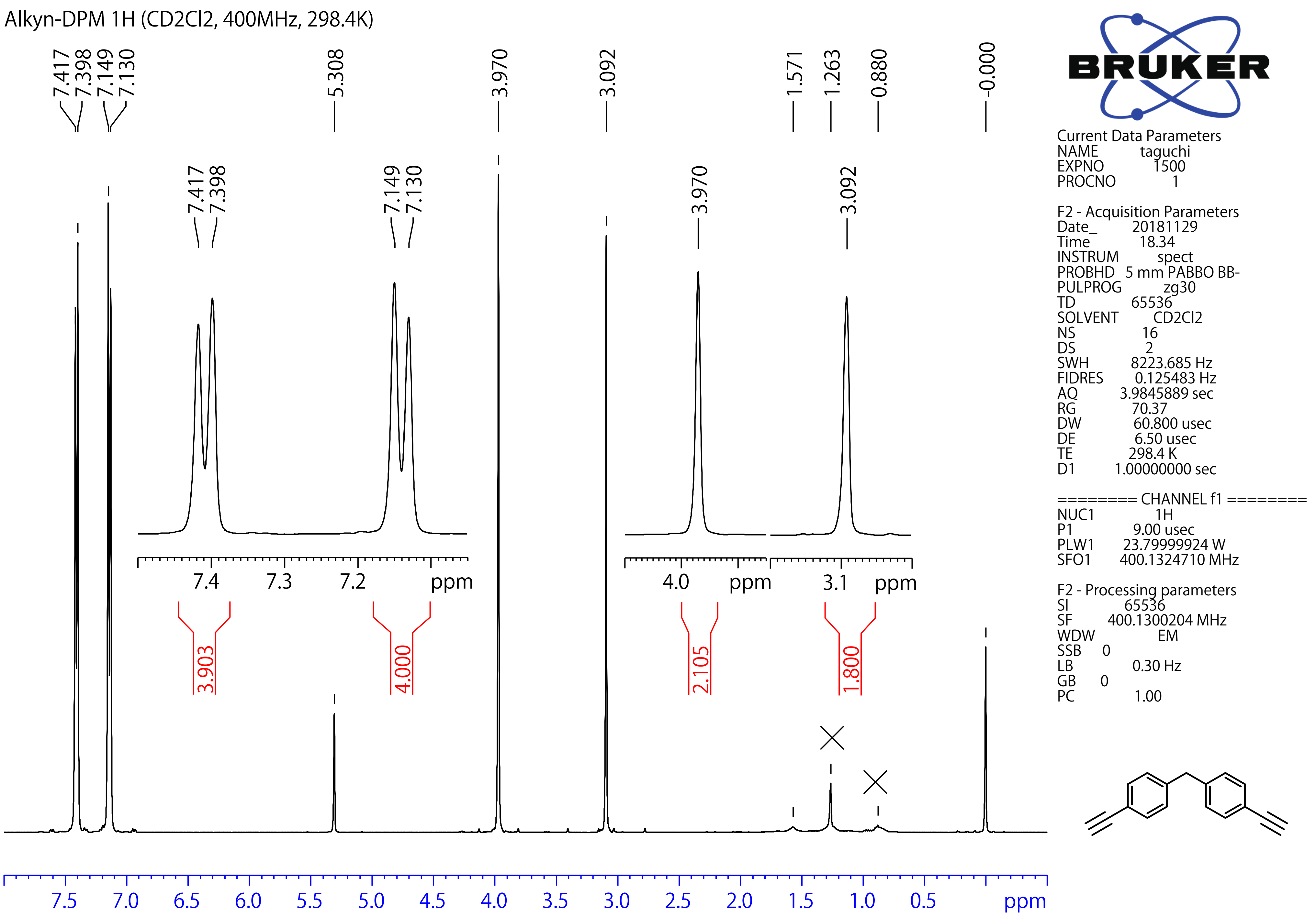

Fig C4. 1H-NMR spectra of compound 3.

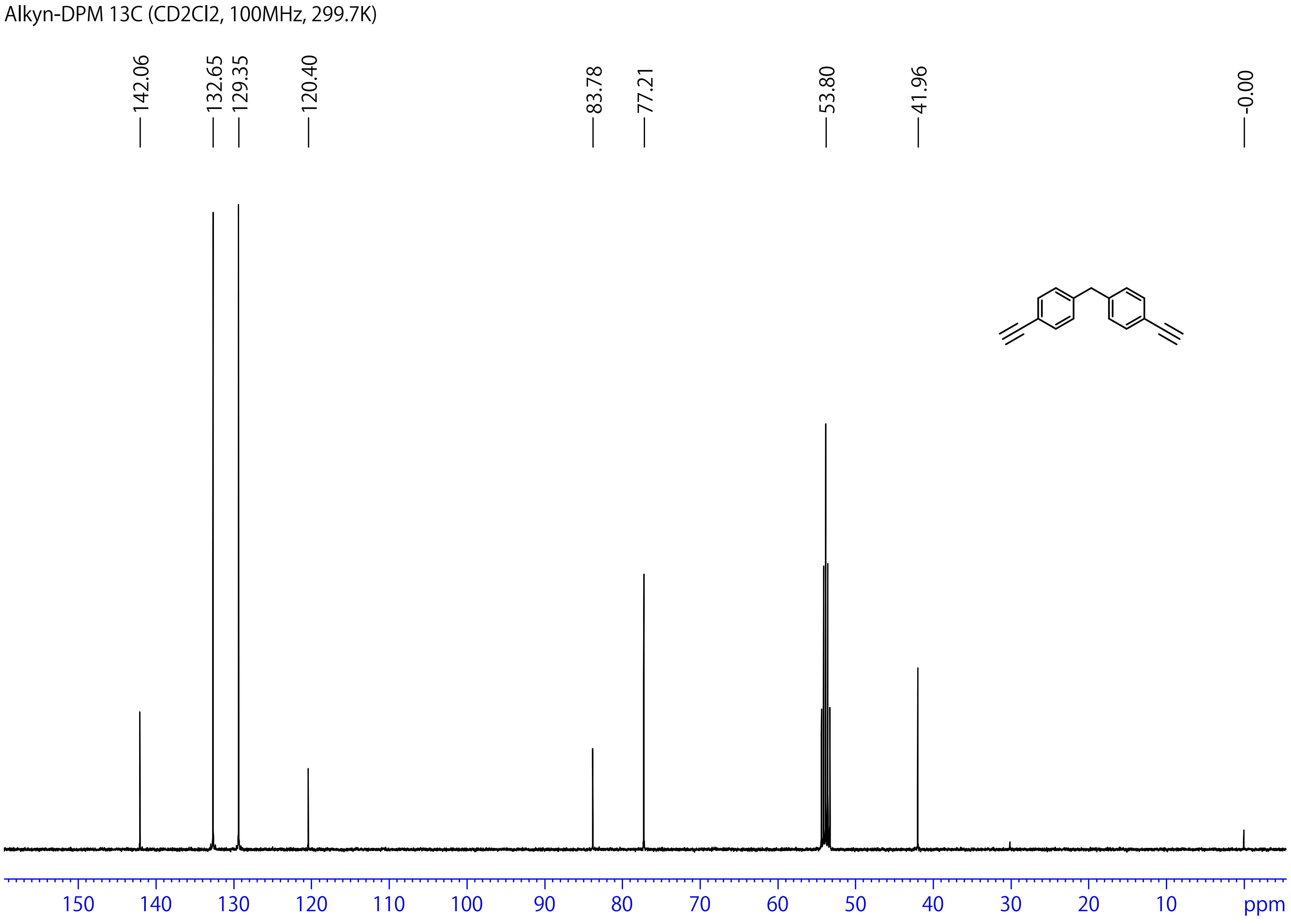

Fig C5. 13C-NMR spectra of compound 3.

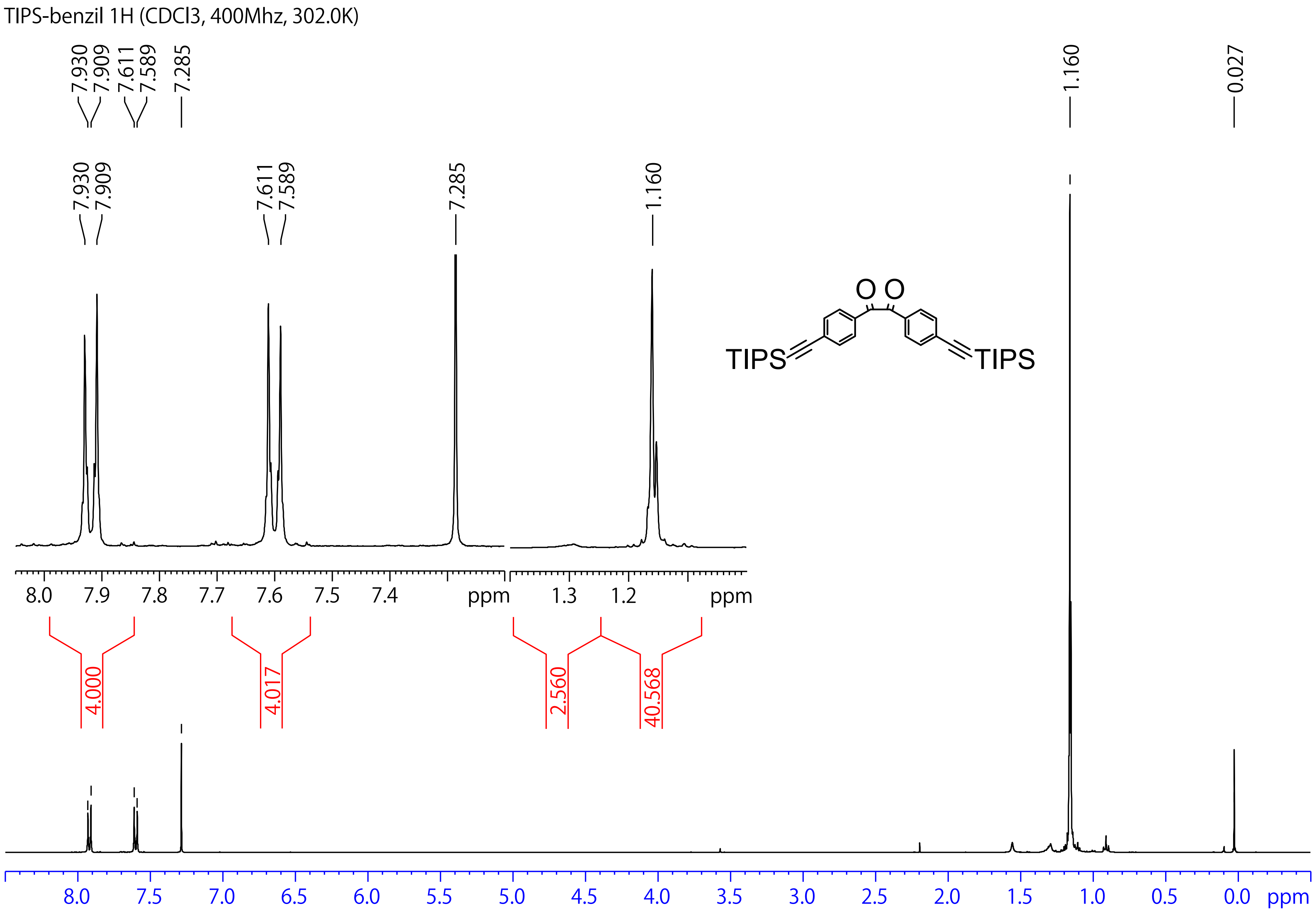

Fig C6. 1H-NMR spectra of compound 4.

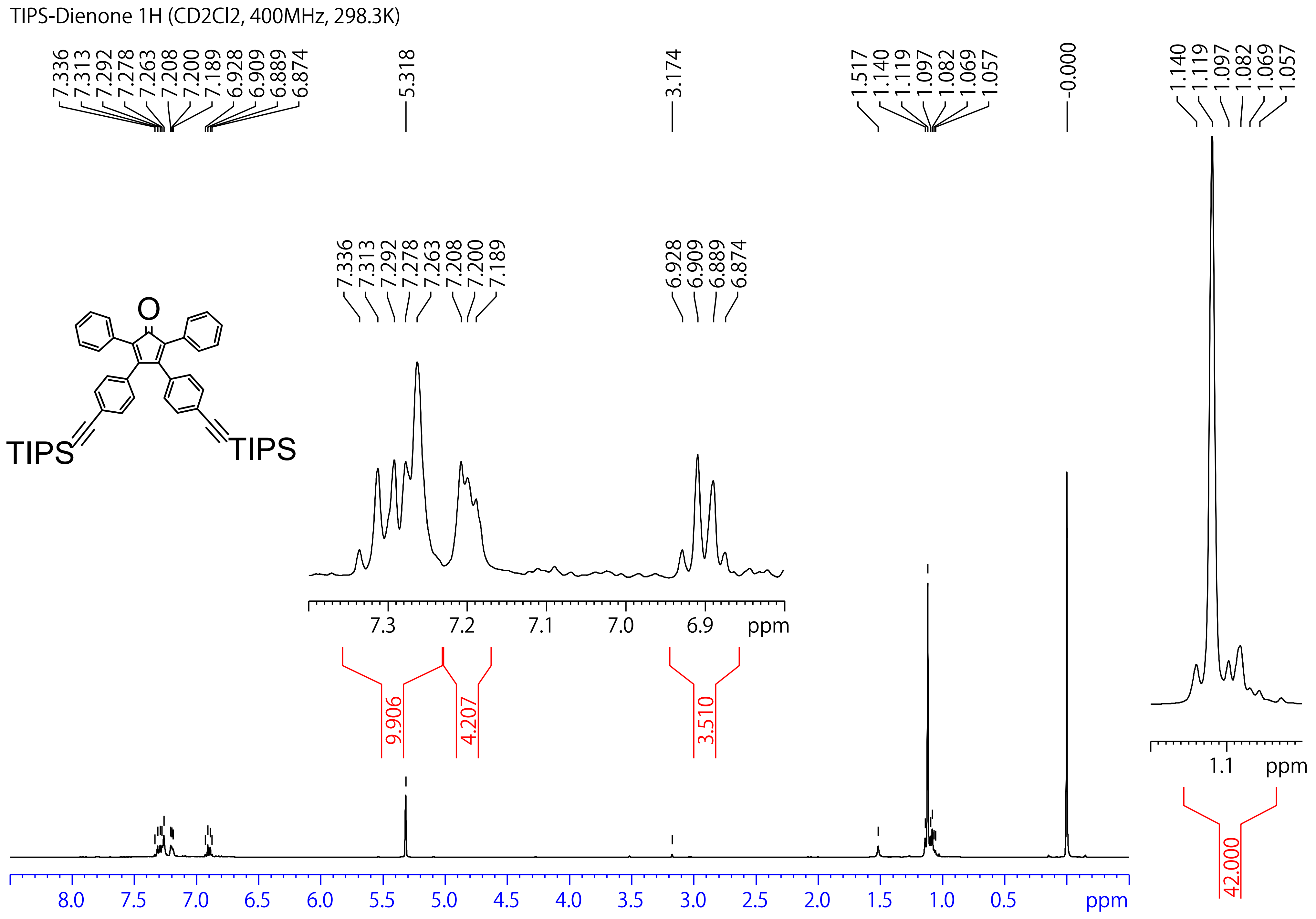

Fig C7. 1H-NMR spectra of compound 5.

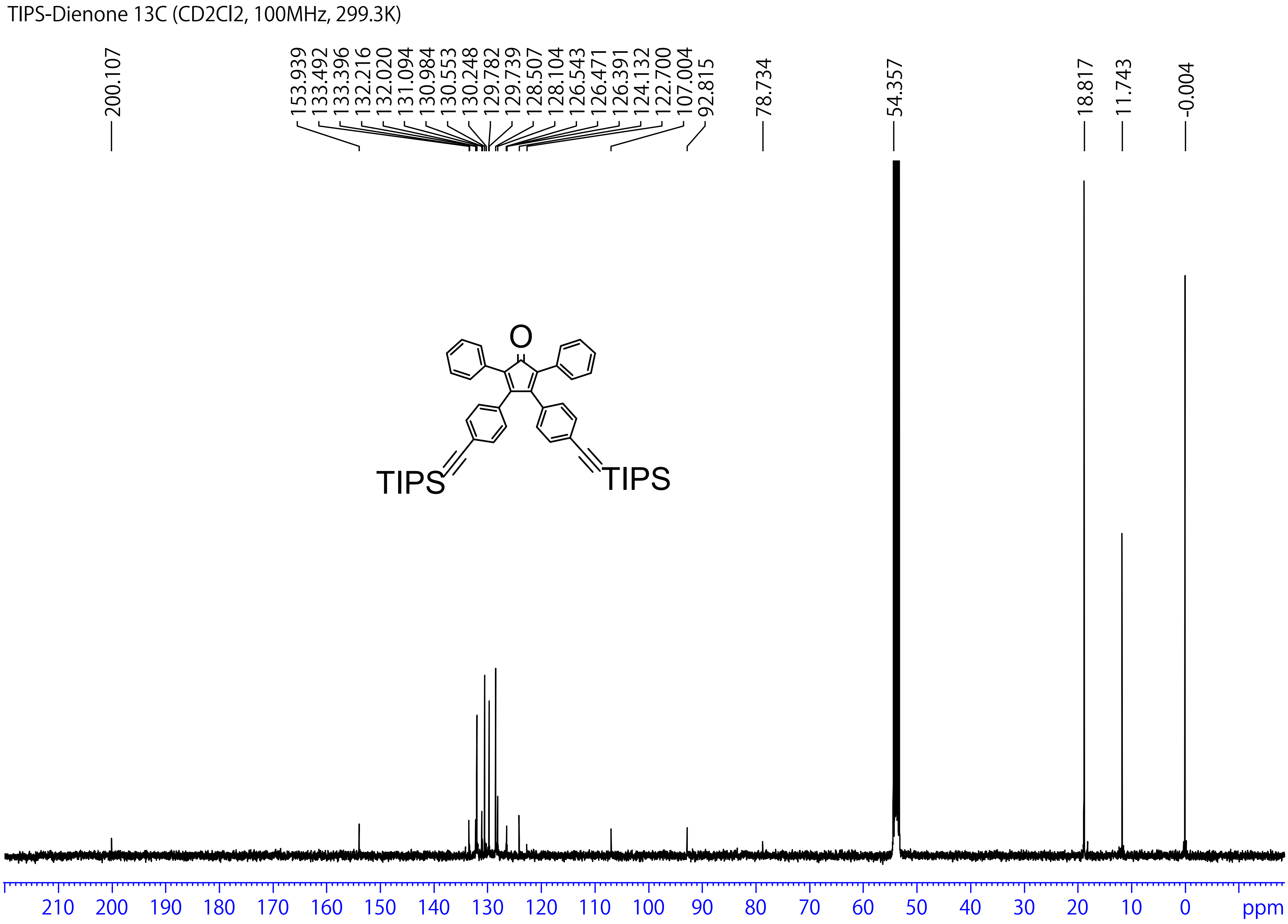

Fig C8. 13C-NMR spectra of compound 5.

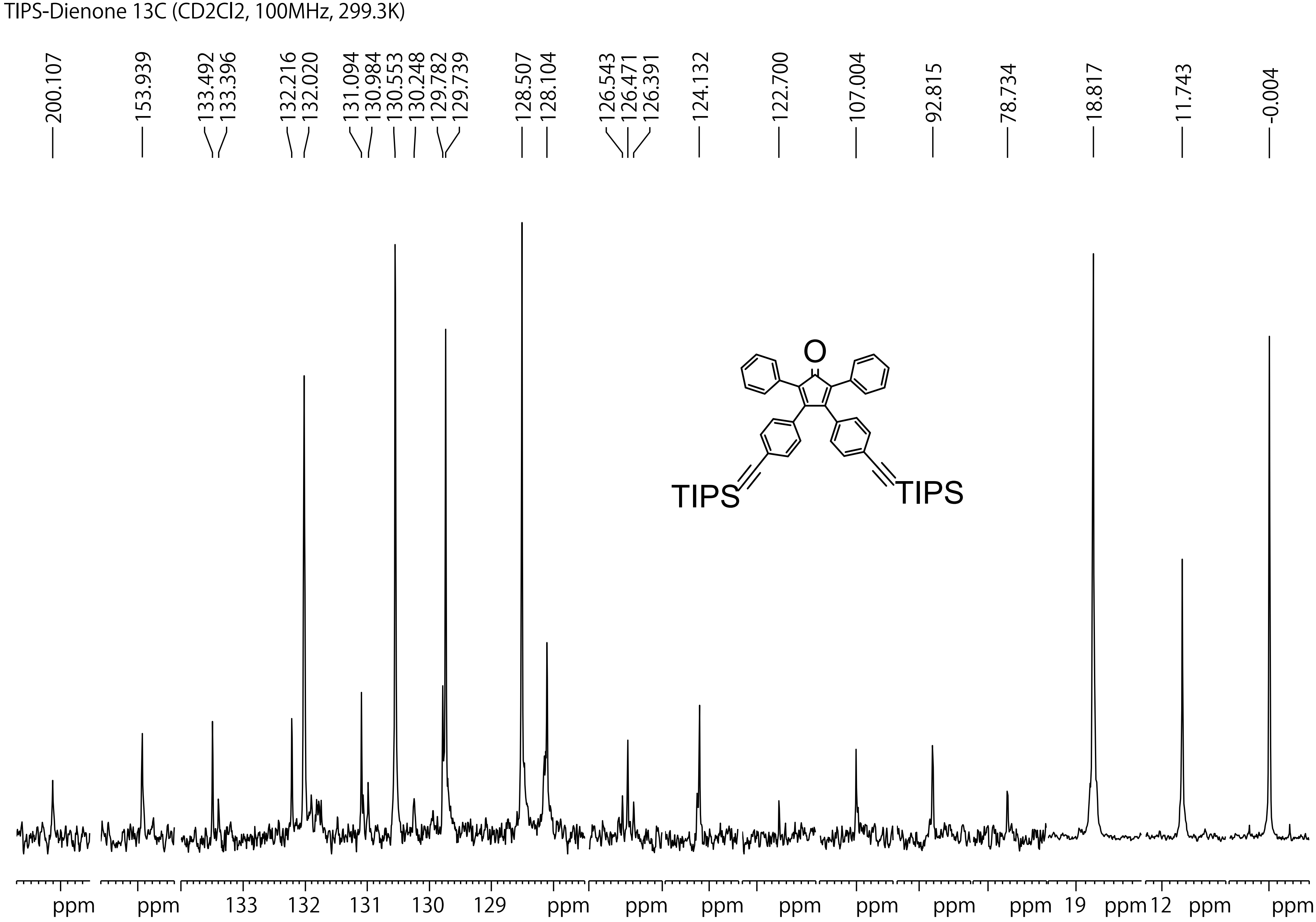

Fig C9. 13C-NMR spectra of compound 5.

TM53 monoacetyl-amino-DPM

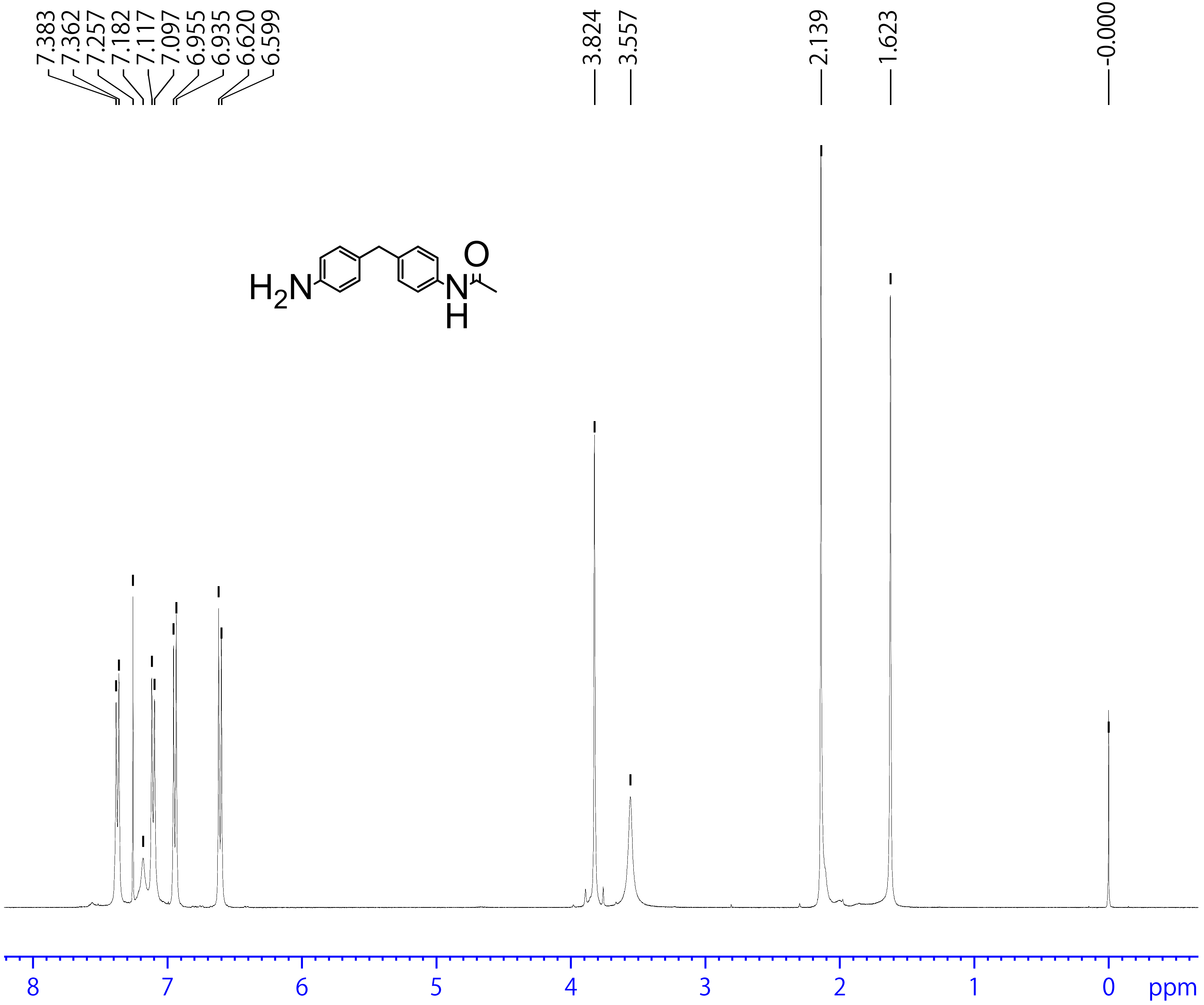

Current Data Parameters
NAME proton only
EXPNO 180
PROCNO 1

F2 - Acquisition Parameters
Date_ 20160726
Time 18.10
INSTRUM spect
PROBHD 5 mm PABBO BB-
PULPROG zg30
TD 65536
SOLVENT CDCl3
NS 16
DS 2
SWH 8223.685 Hz
FIDRES 0.125483 Hz
AQ 3.9845889 sec
RG 107.46
DW 60.800 usec
DE 6.50 usec
TE 304.5 K
D1 1.00000000 sec

======== CHANNEL f1 ========
NUC1 1H
P1 9.00 usec
PLW1 23.79999924 W
SFO1 400.1324710 MHz

F2 - Processing parameters
SI 65536
SF 400.1300109 MHz
WDW EM
SSB 0
LB 0.30 Hz
GB 0
PC 1.00

Fig C10. 1H-NMR spectra of compound 6.

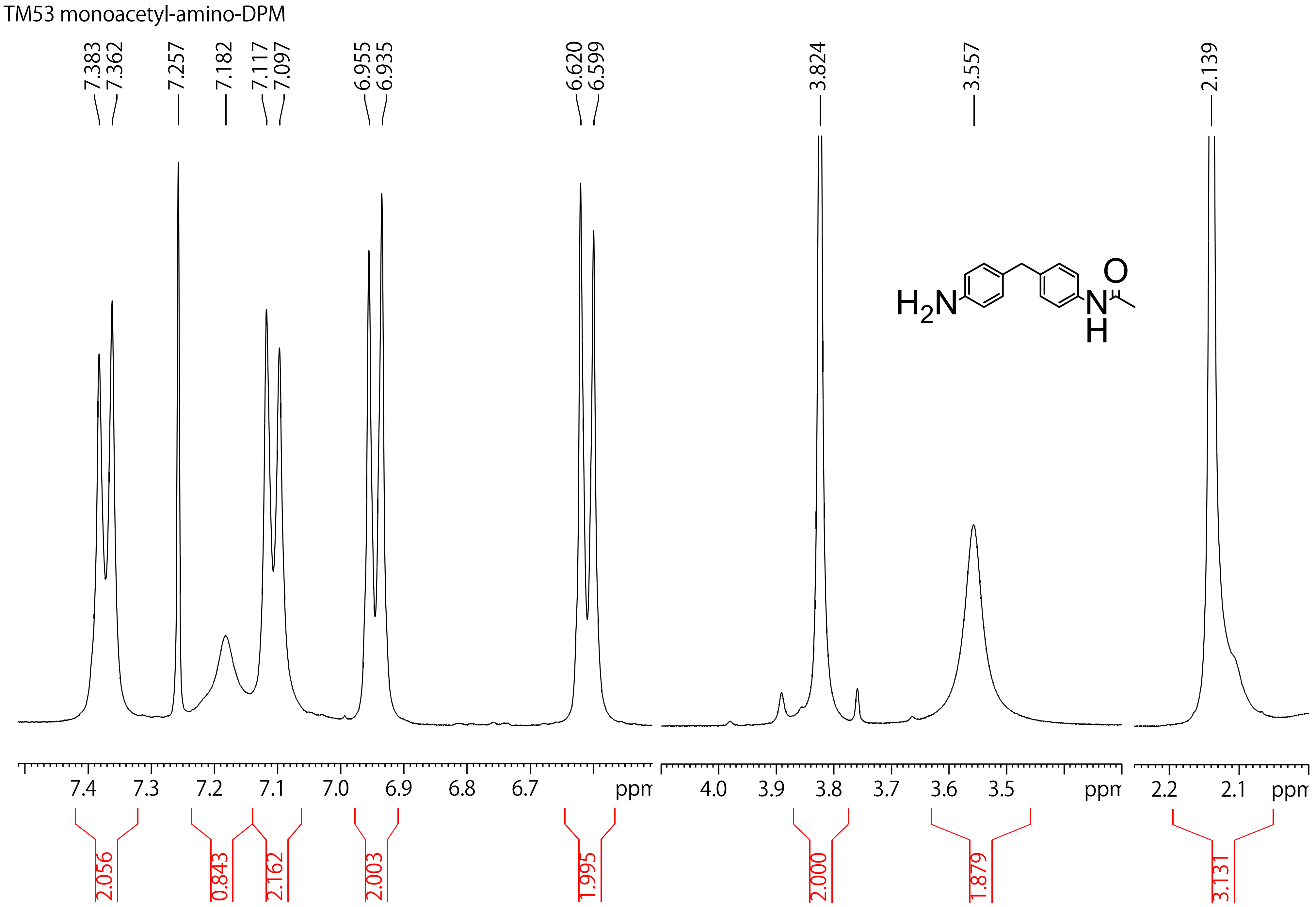

Fig C11. 1H-NMR spectra of compound 6.

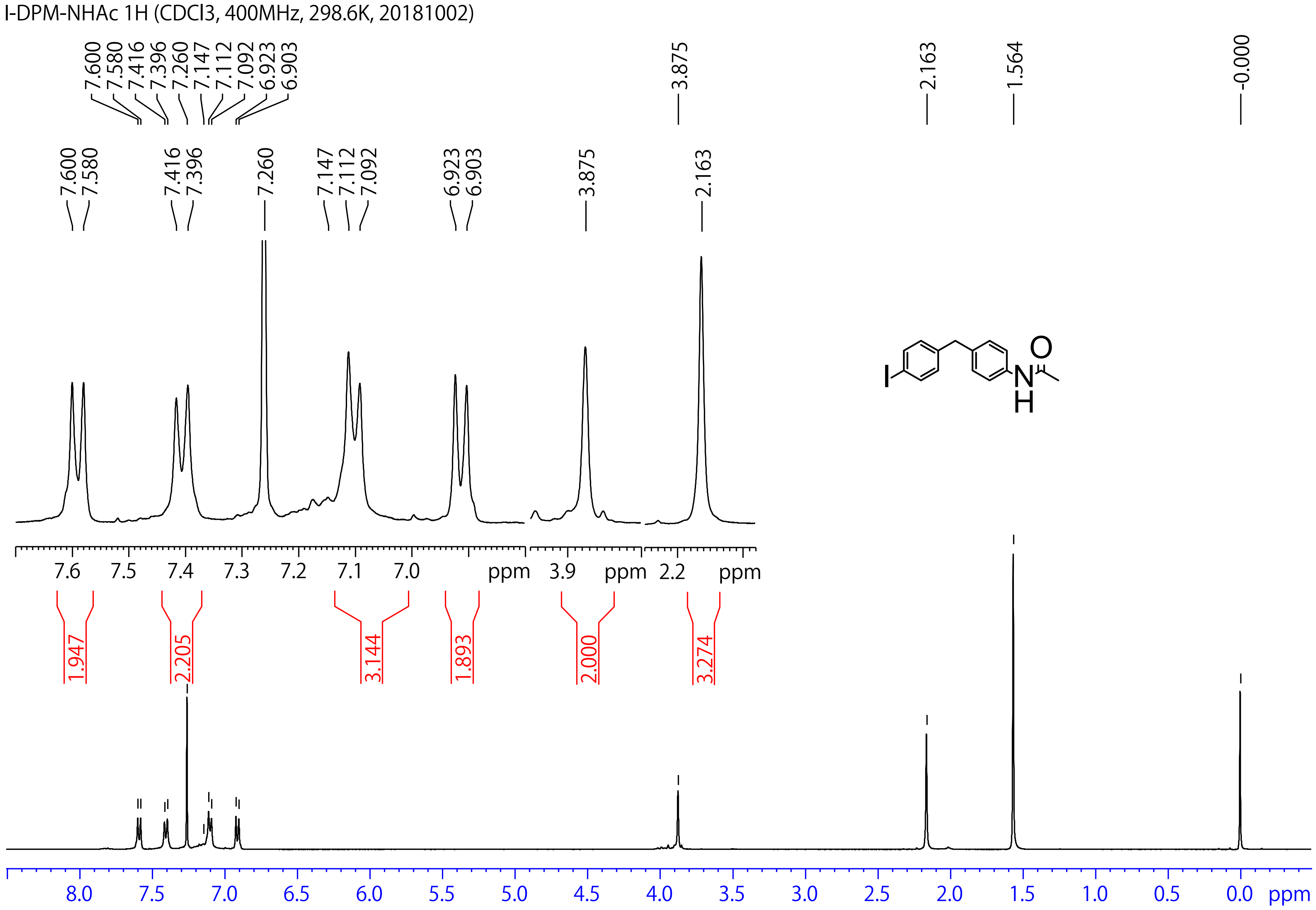

Fig C12. 1H-NMR spectra of compound 7.

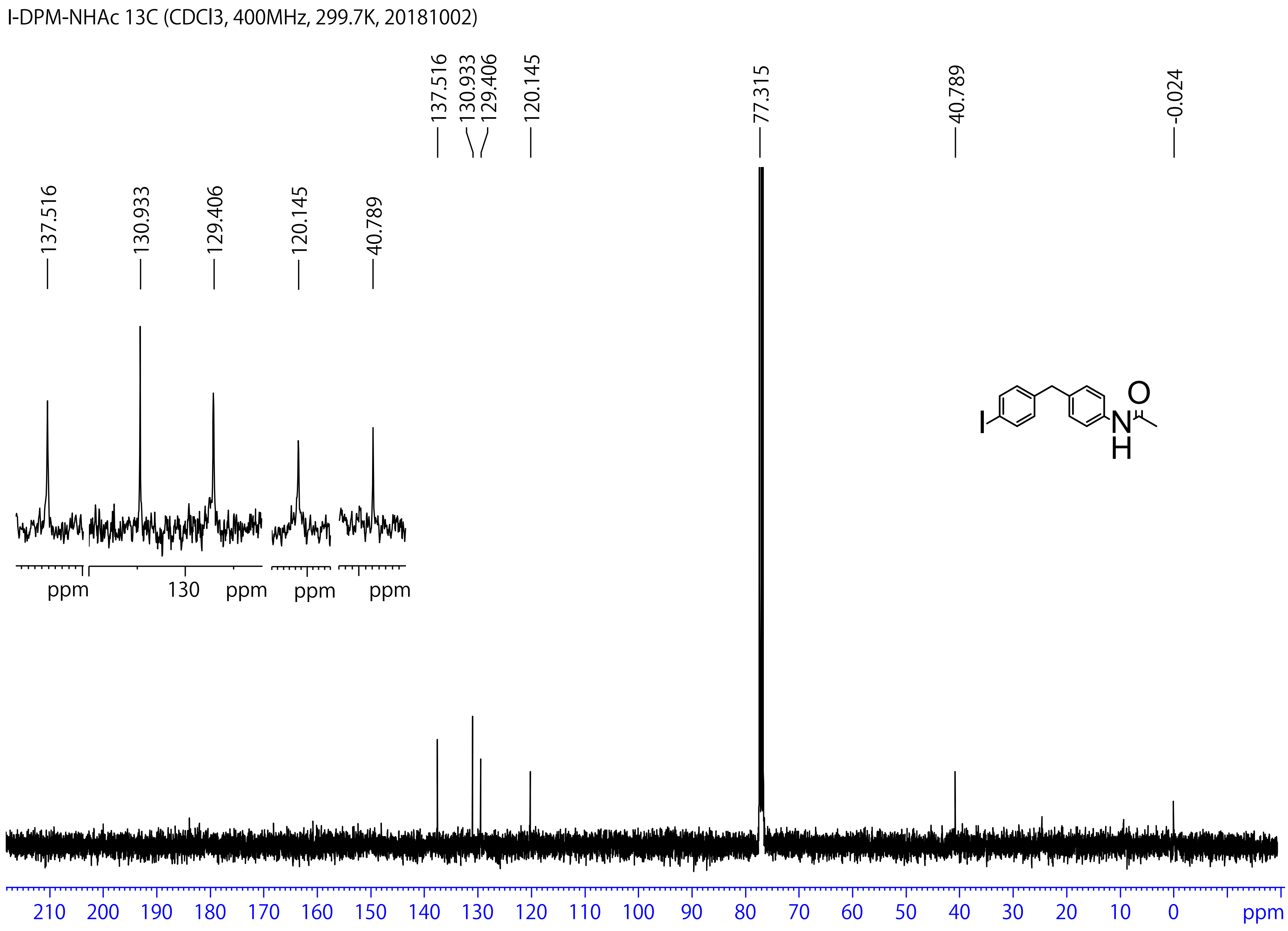

Fig C13. 13C-NMR spectra of compound 7.

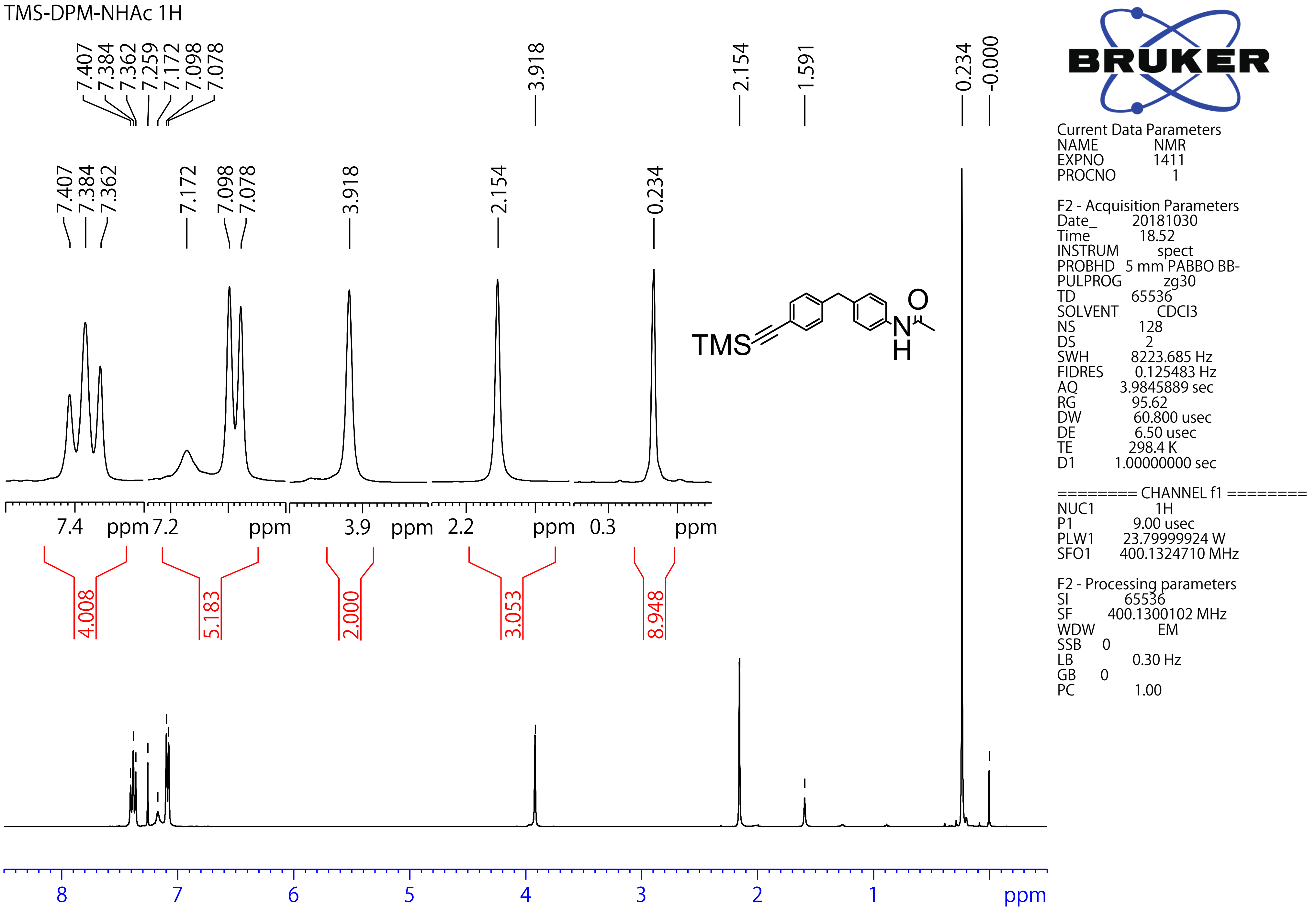

Fig C14. 1H-NMR spectra of compound 8.

TMS-DPM-NHAc 13C (CDCl3, 100MHz, 299.1K))

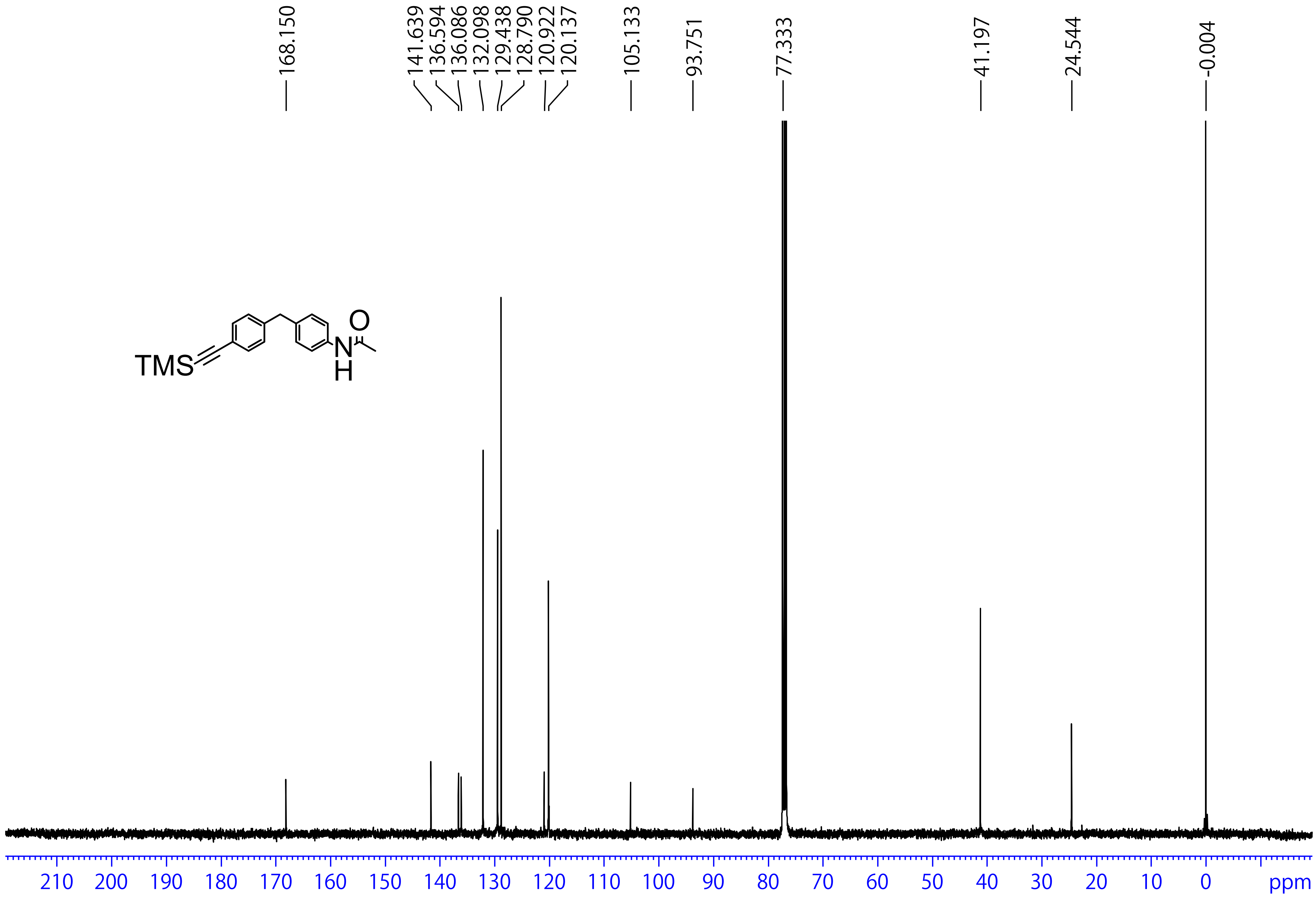

Fig C15. 13C-NMR spectra of compound 8.

Alkyn-DPM-NHAc 1H (CD2Cl2, 400MHz, 298.3K)

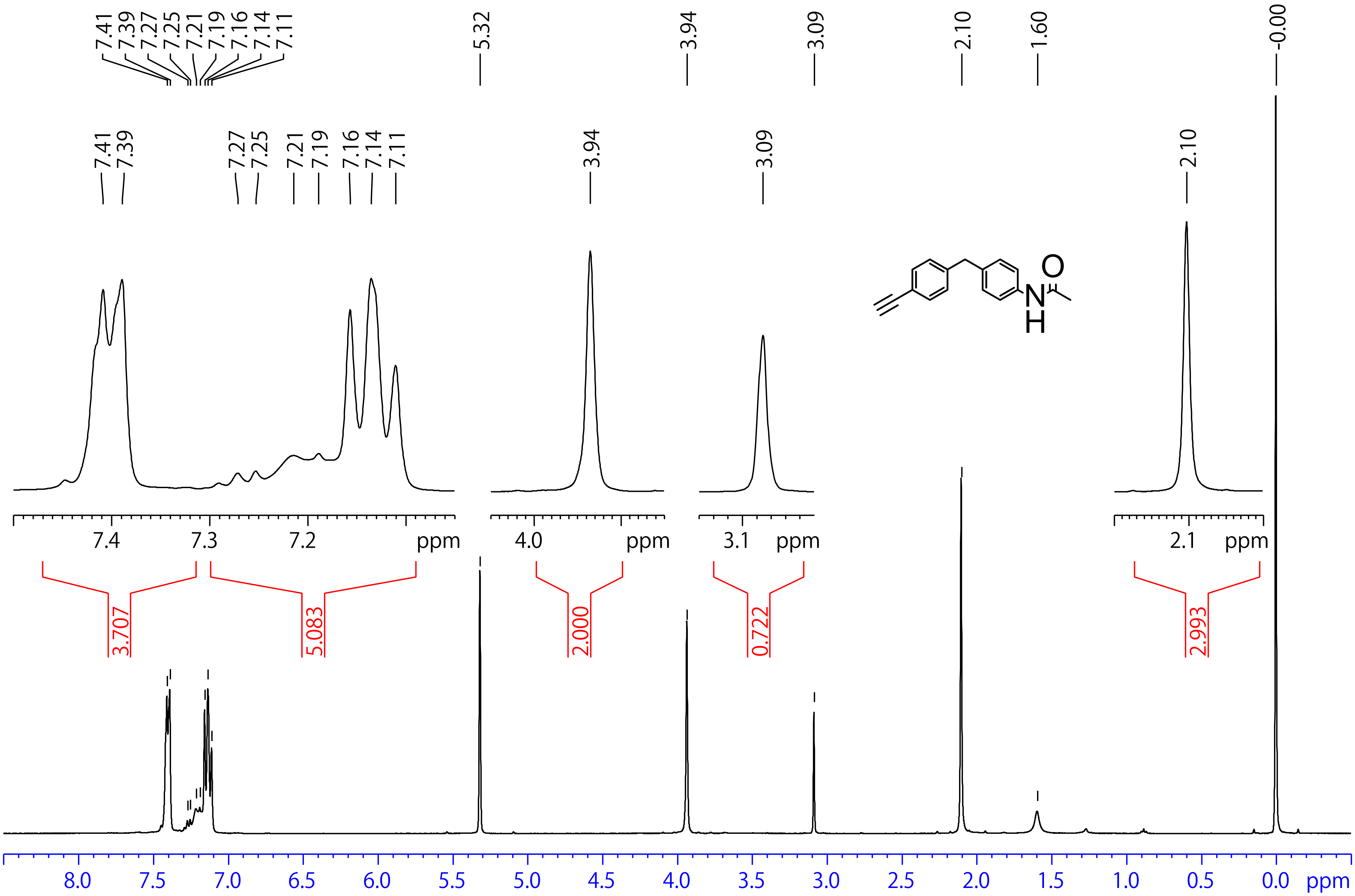

Fig C16. 1H-NMR spectra of compound 9.

Alkyn-DPM-NHAc 13C (CD2Cl2, 100MHz, 299.5K)

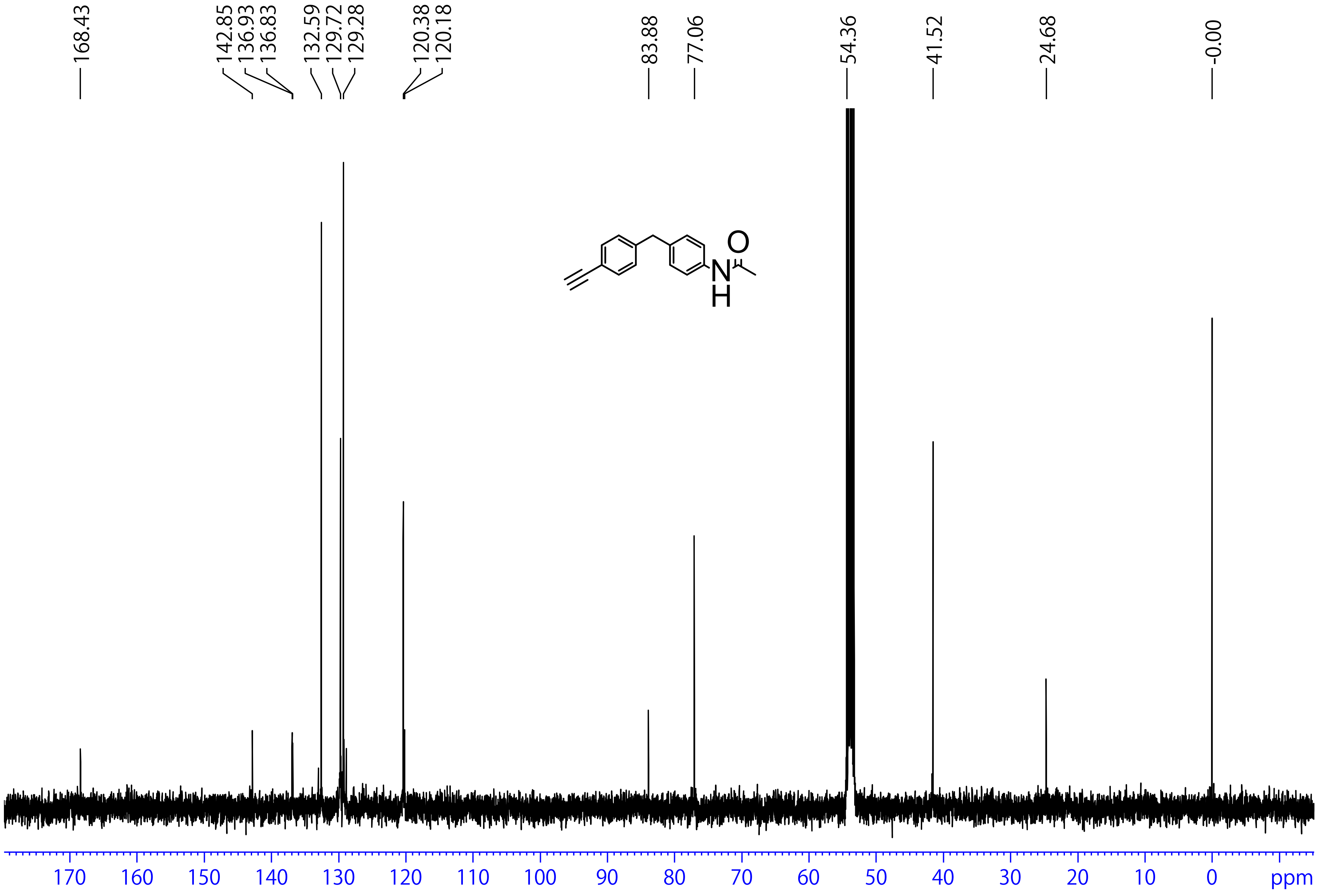

Fig C17. 13C-NMR spectra of compound 9.

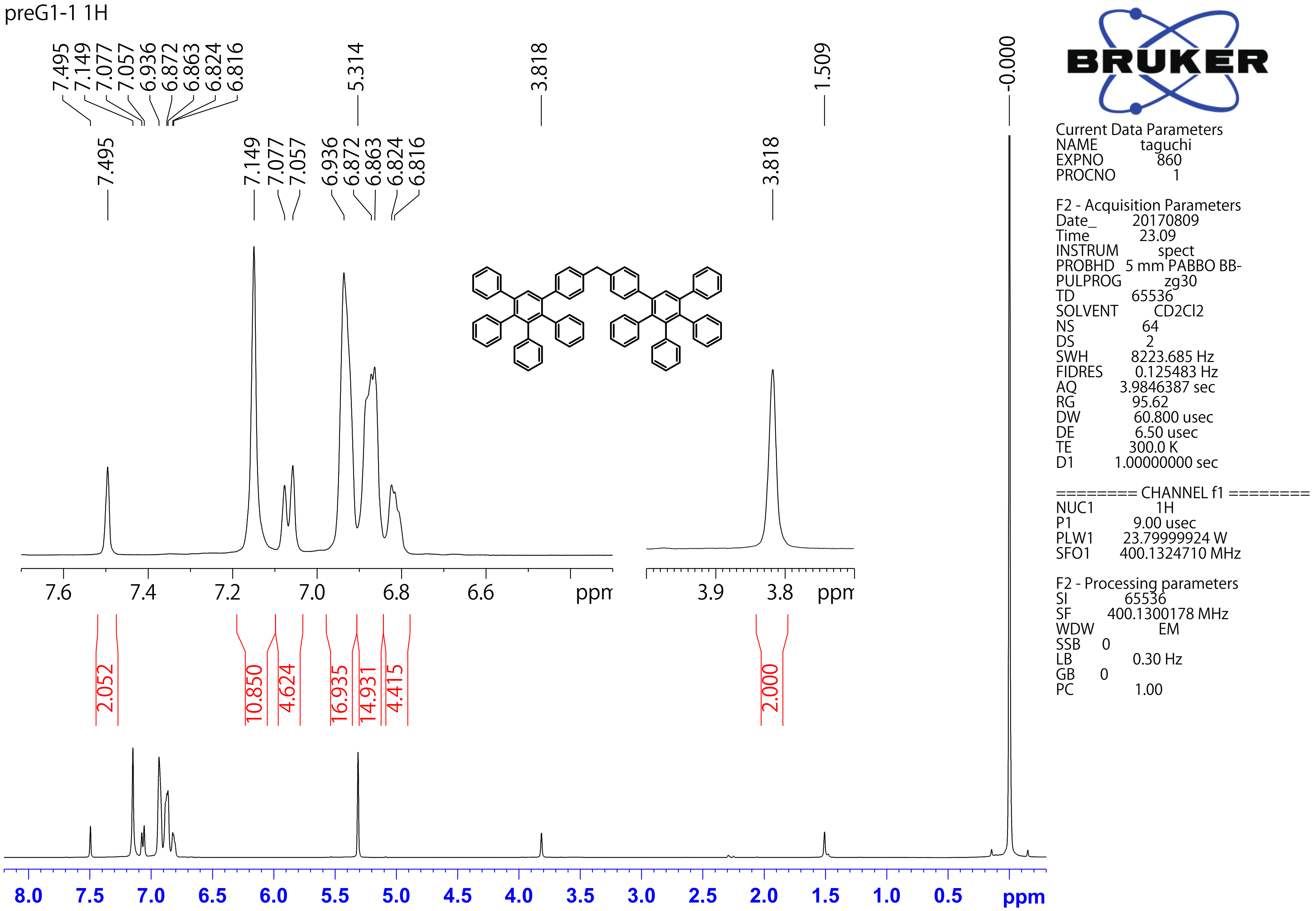

Fig C18. 1H-NMR spectra of compound 10.

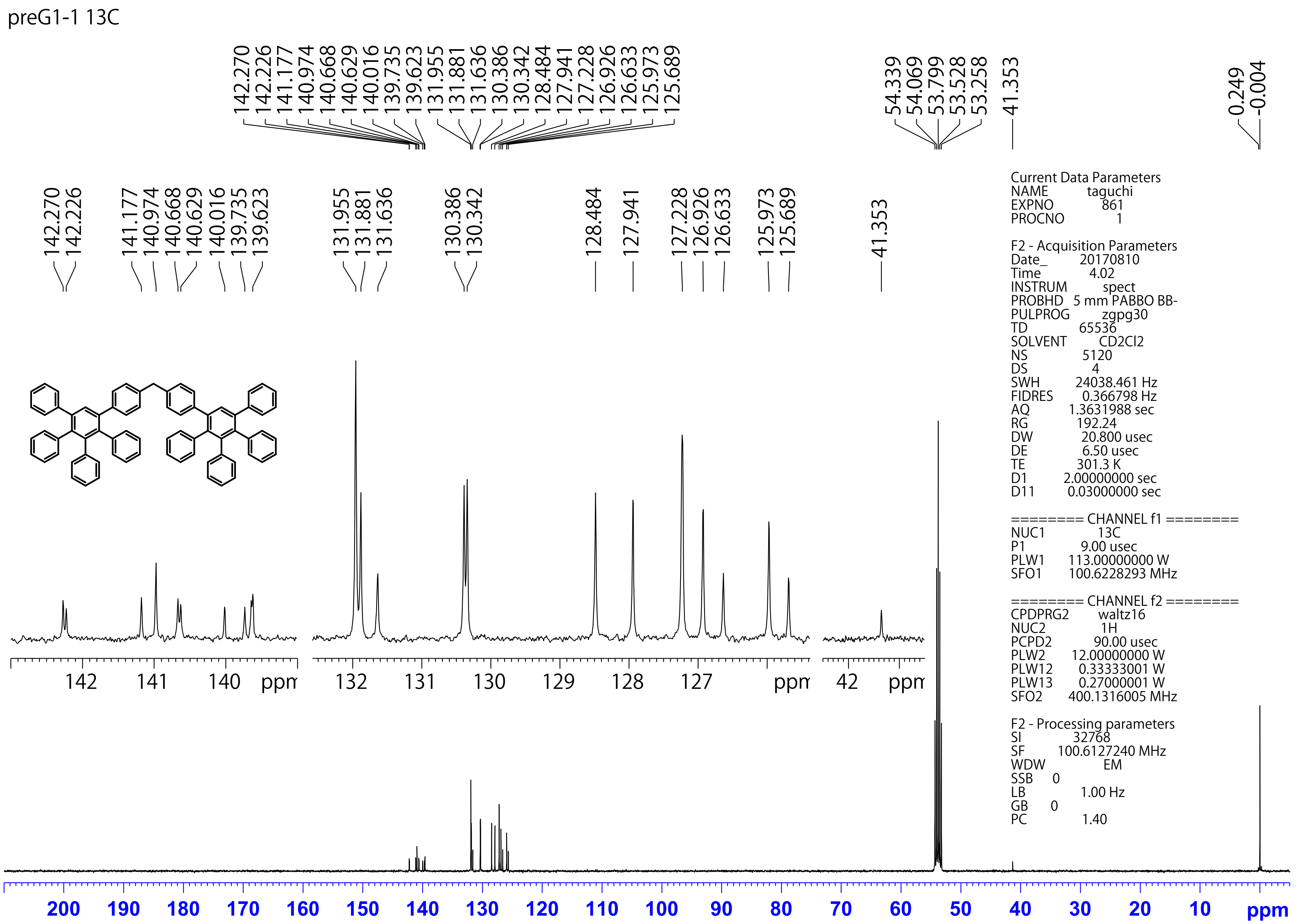

Fig C19. 13C-NMR spectra of compound 10.

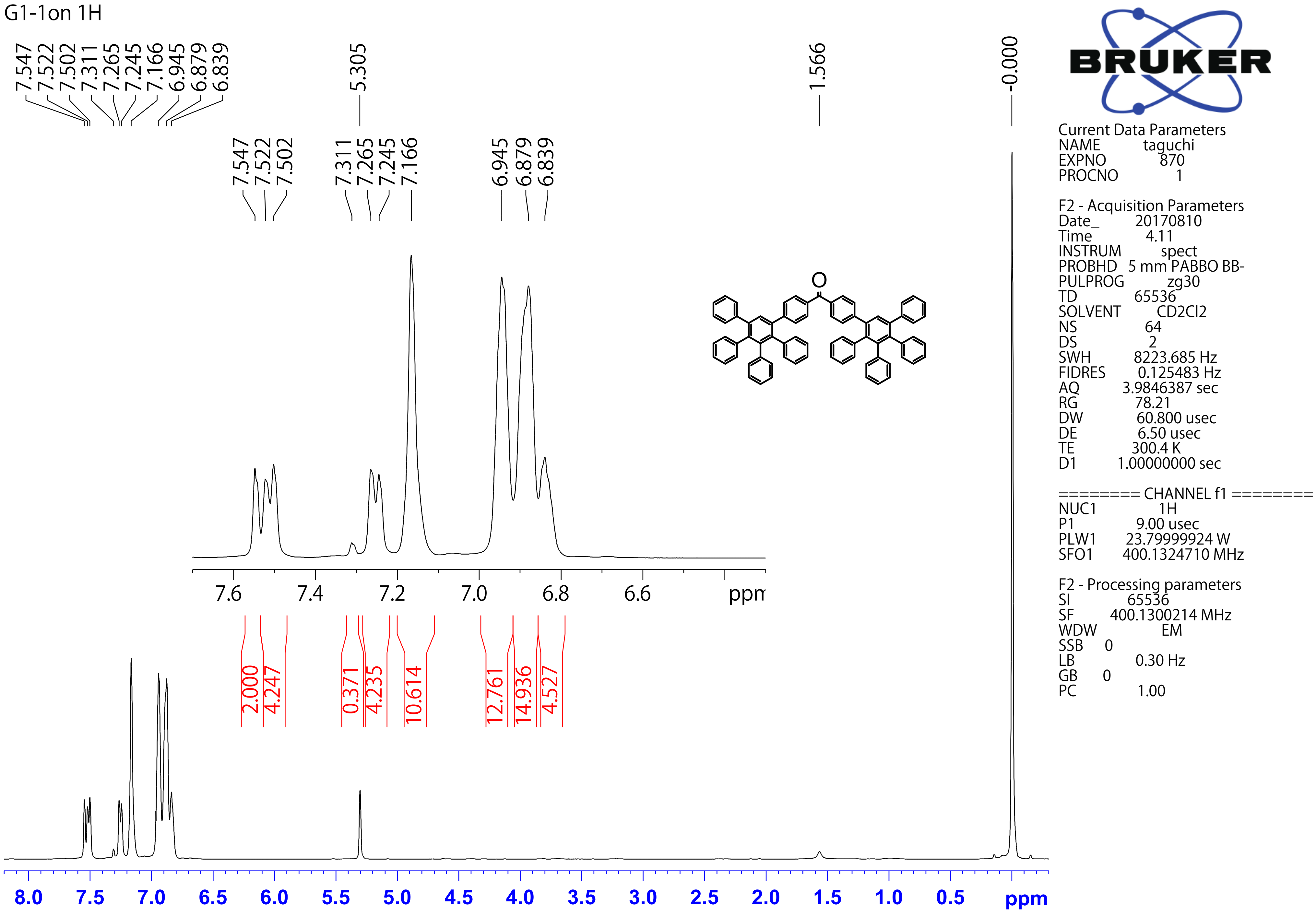

Fig C20. 1H-NMR spectra of compound 11.

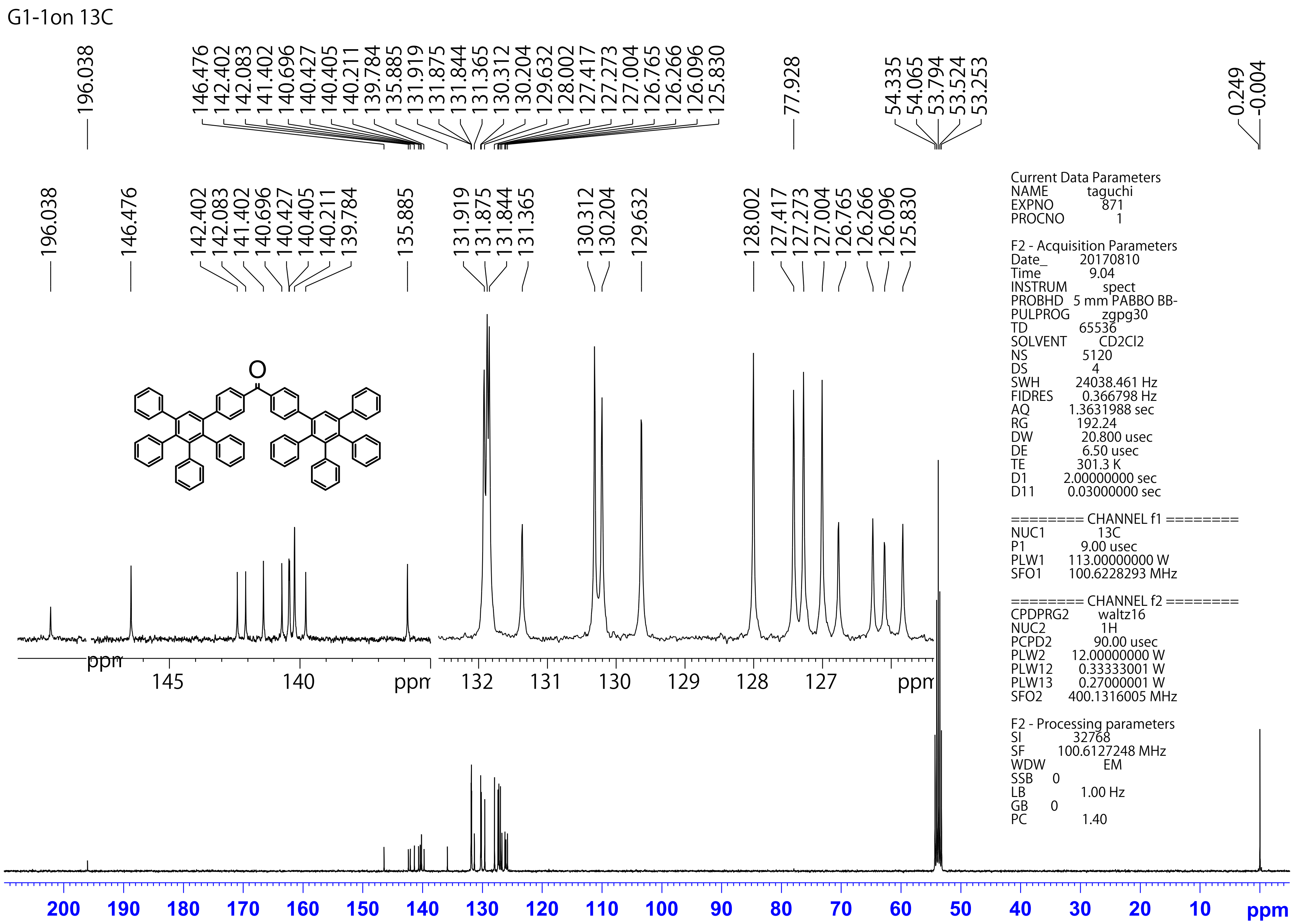

Fig C21. 13C-NMR spectra of compound 11.

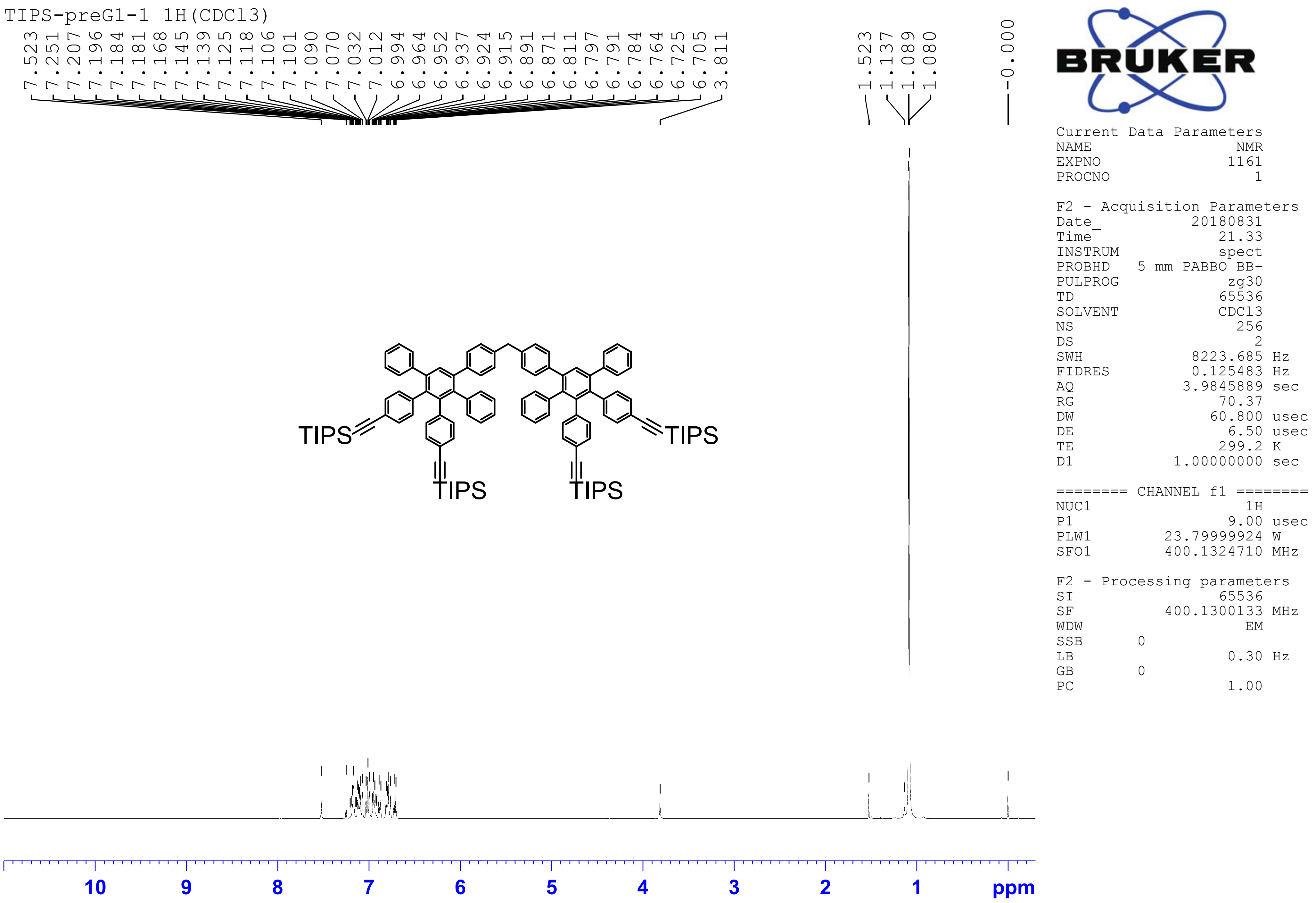

Fig C22. 1H-NMR spectra of compound 12.

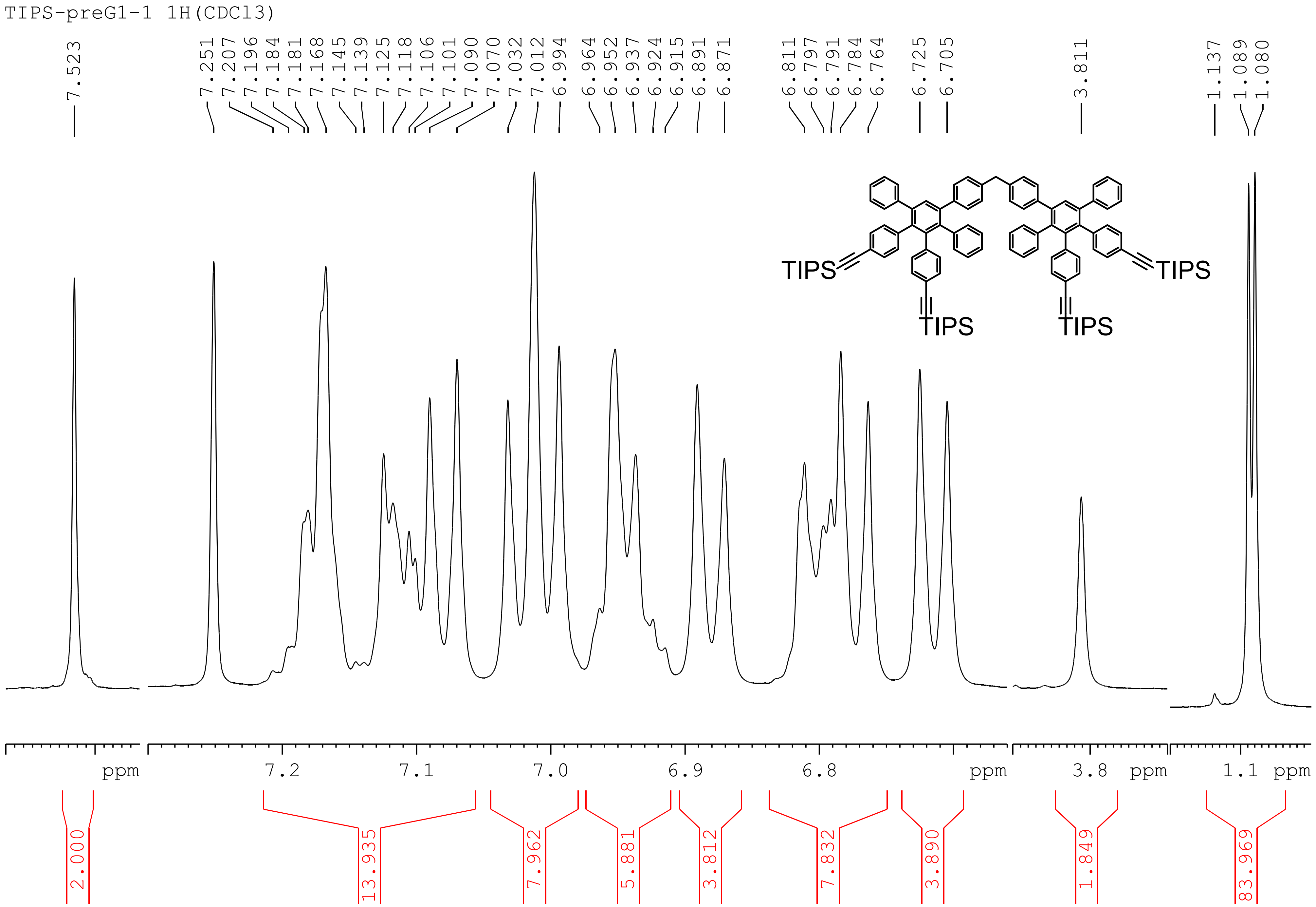

Fig C23. 1H-NMR spectra of compound 12.

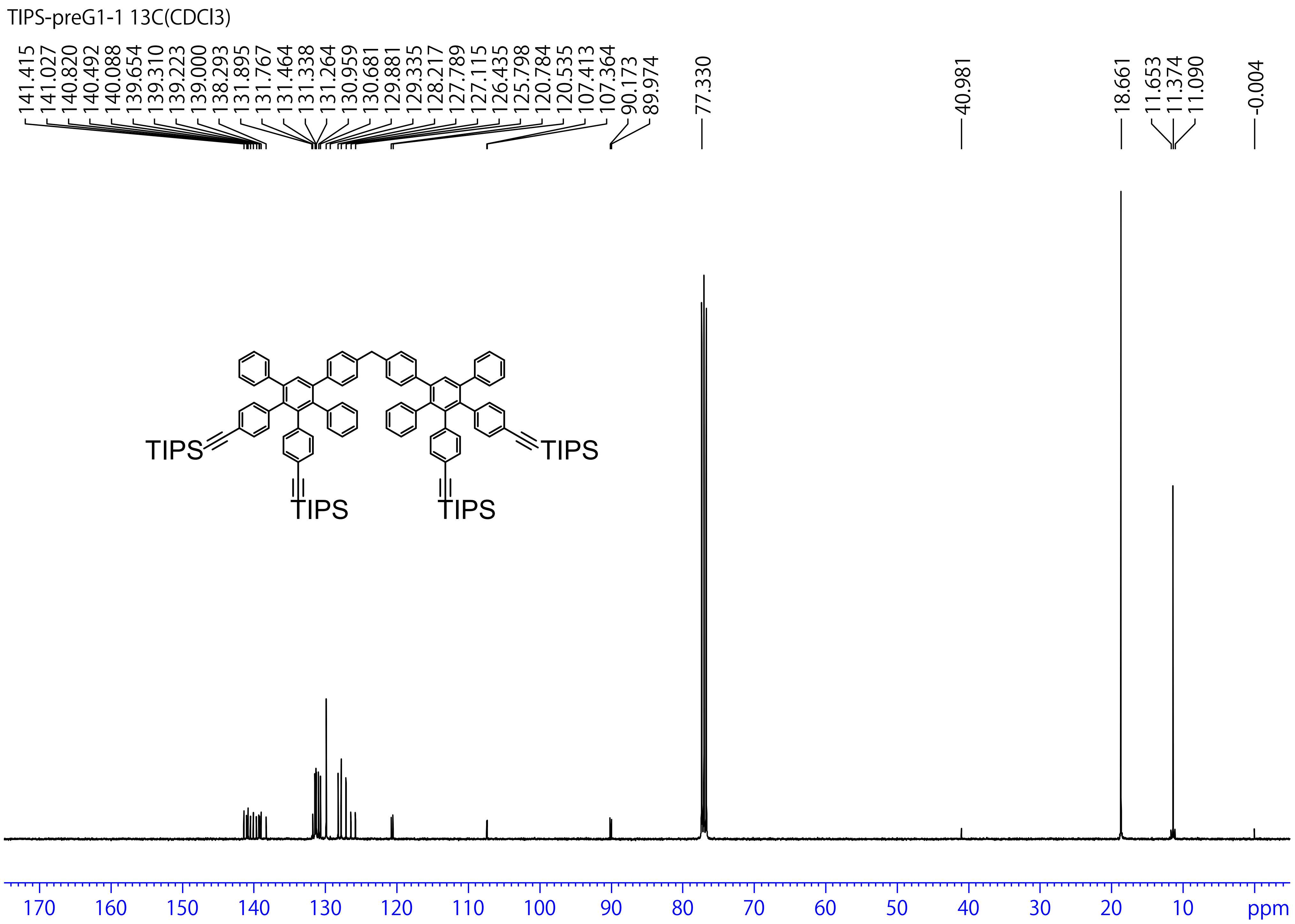

Fig C24. 13C-NMR spectra of compound 12.

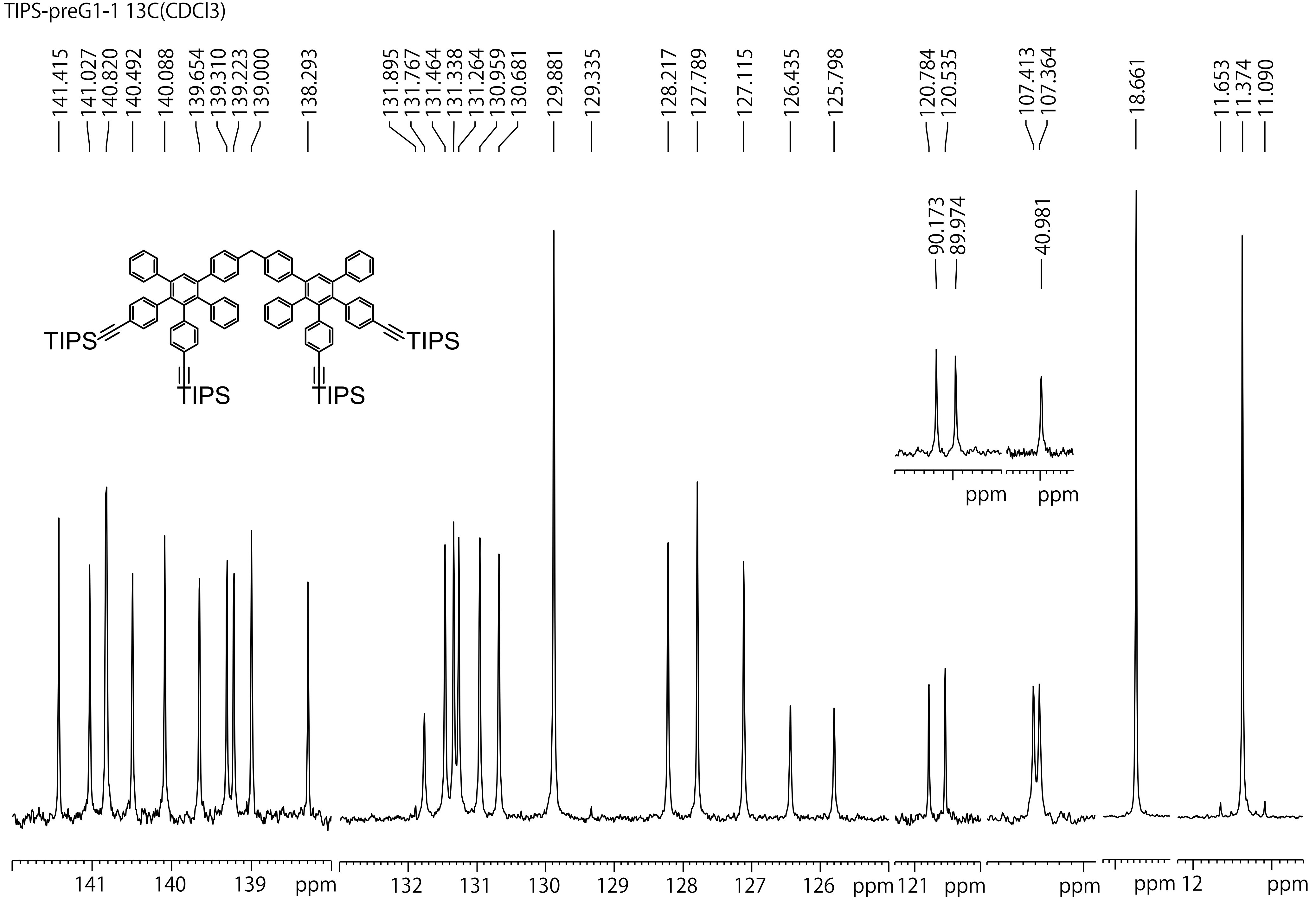

Fig C25. 13C-NMR spectra of compound 12.

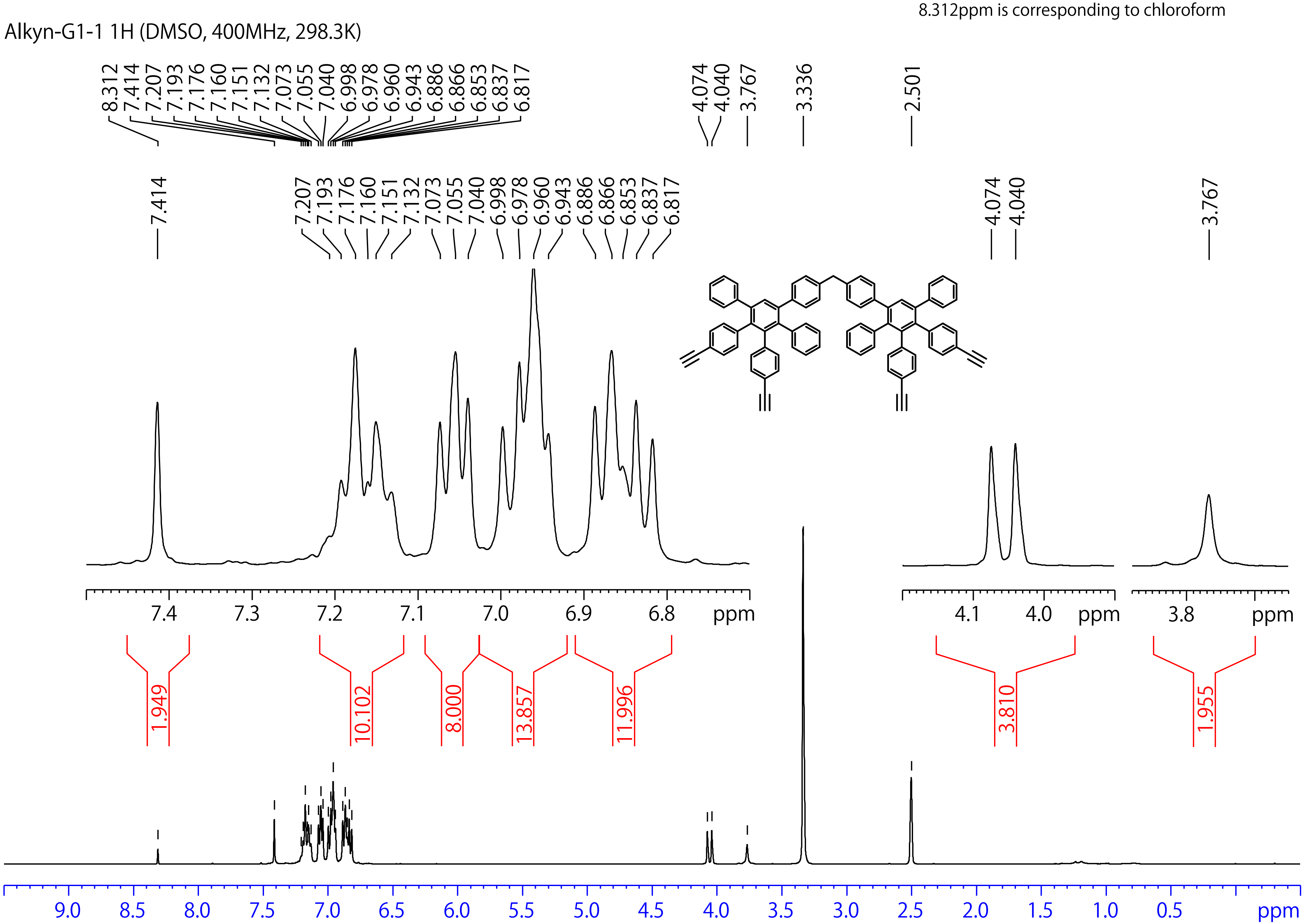

Fig C26. 1H-NMR spectra of compound 13.

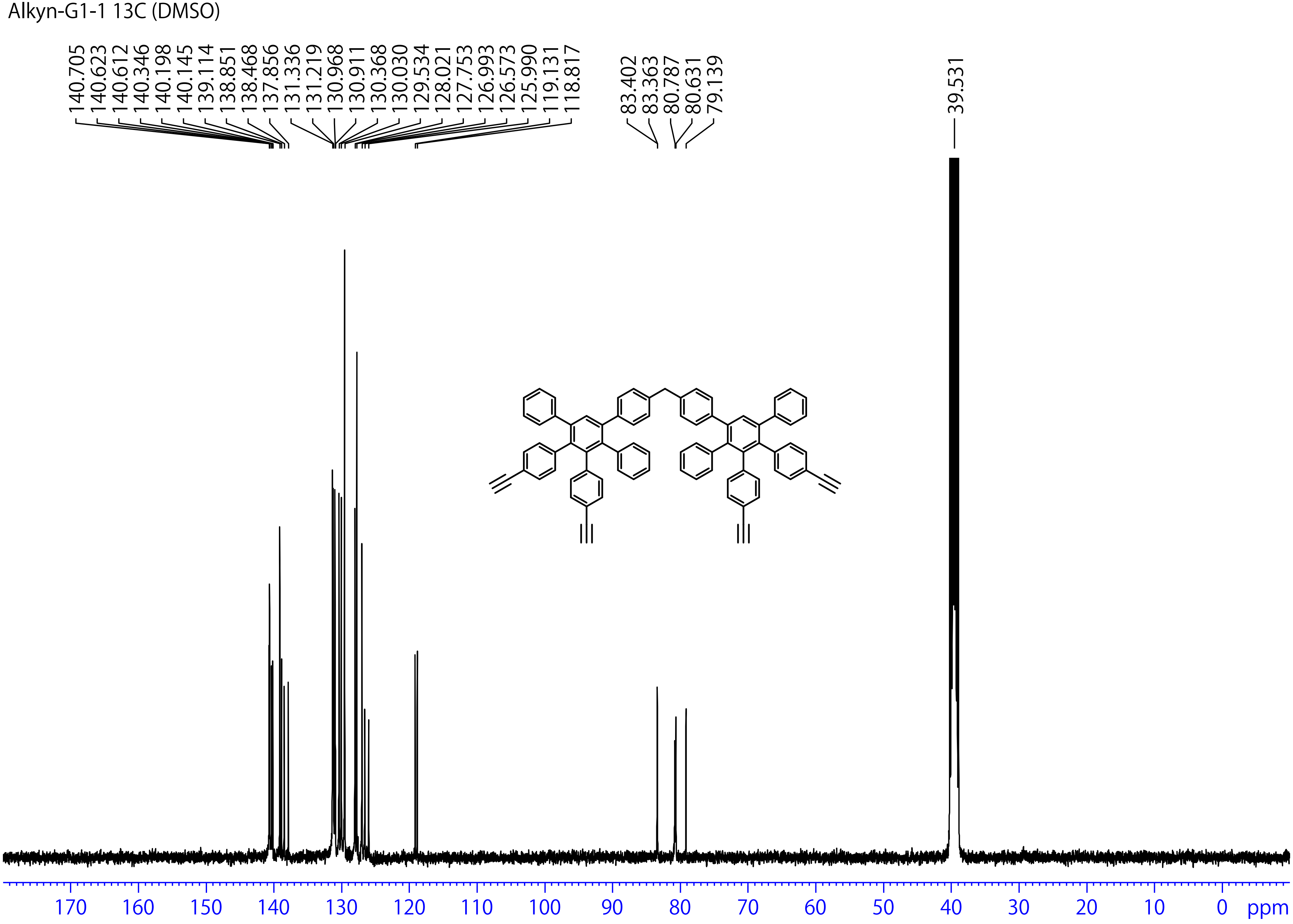

Fig C27. 13C-NMR spectra of compound 13.

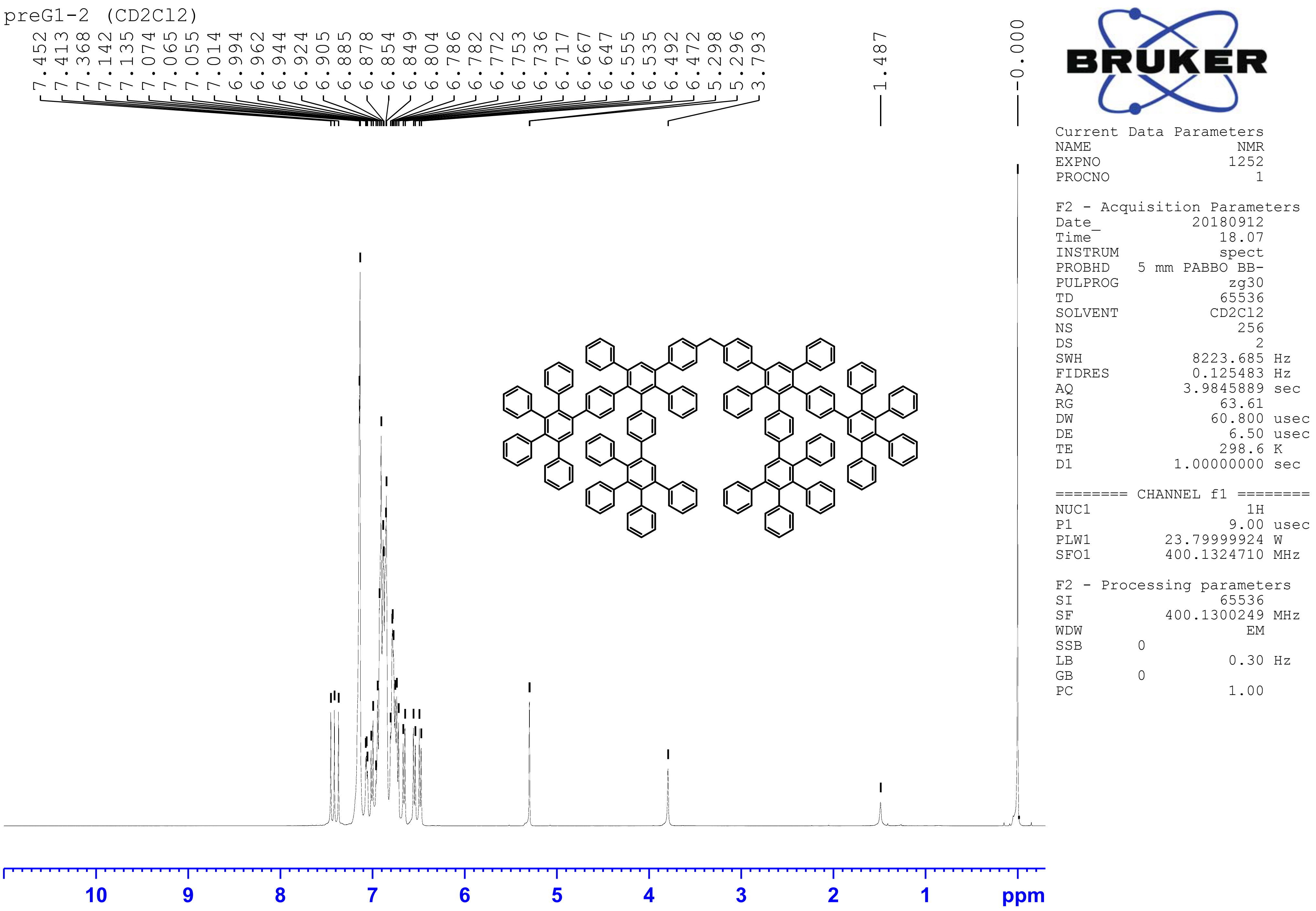

Fig C28. 1H-NMR spectra of compound 14.

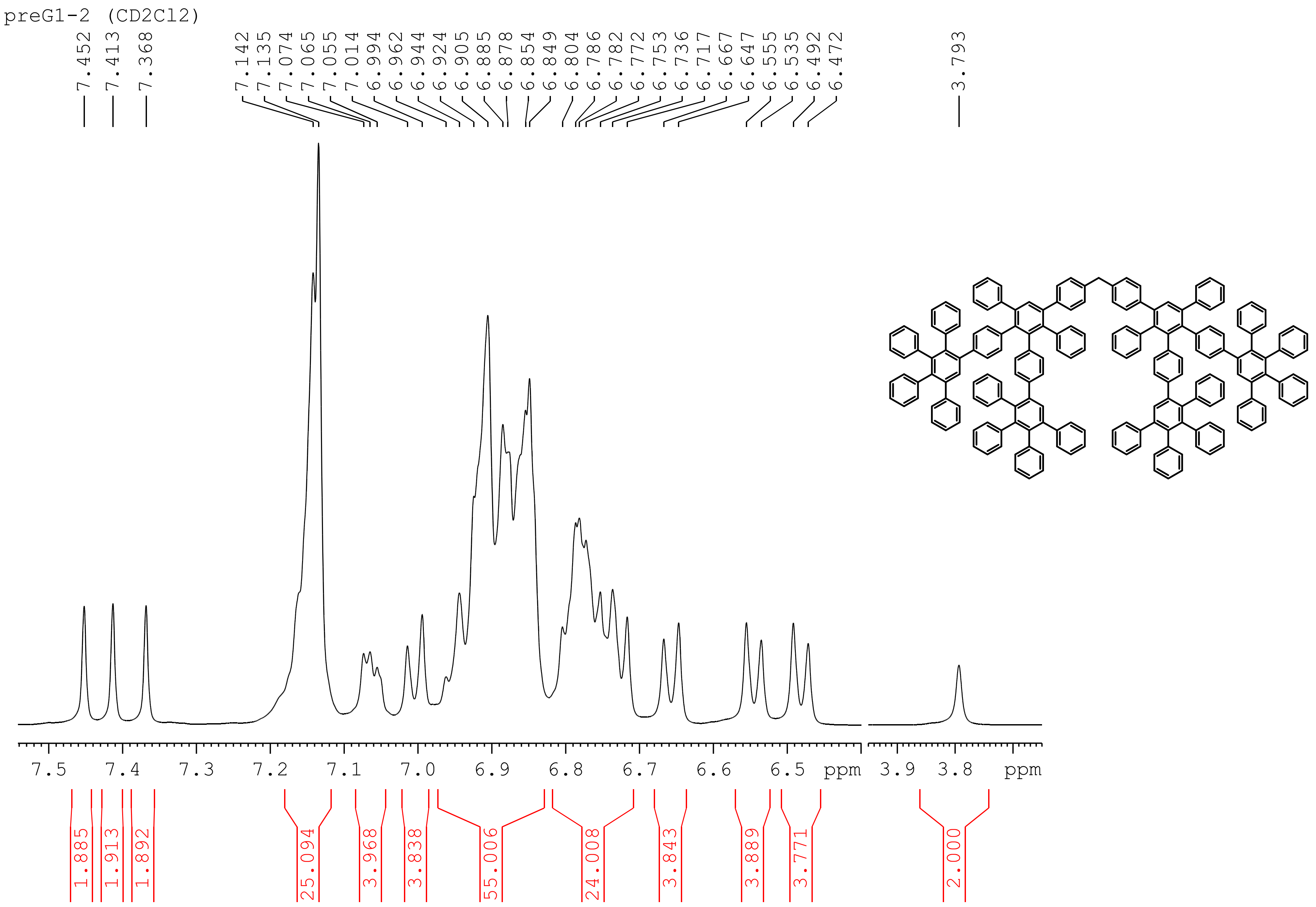

Fig C29. 1H-NMR spectra of compound 14.

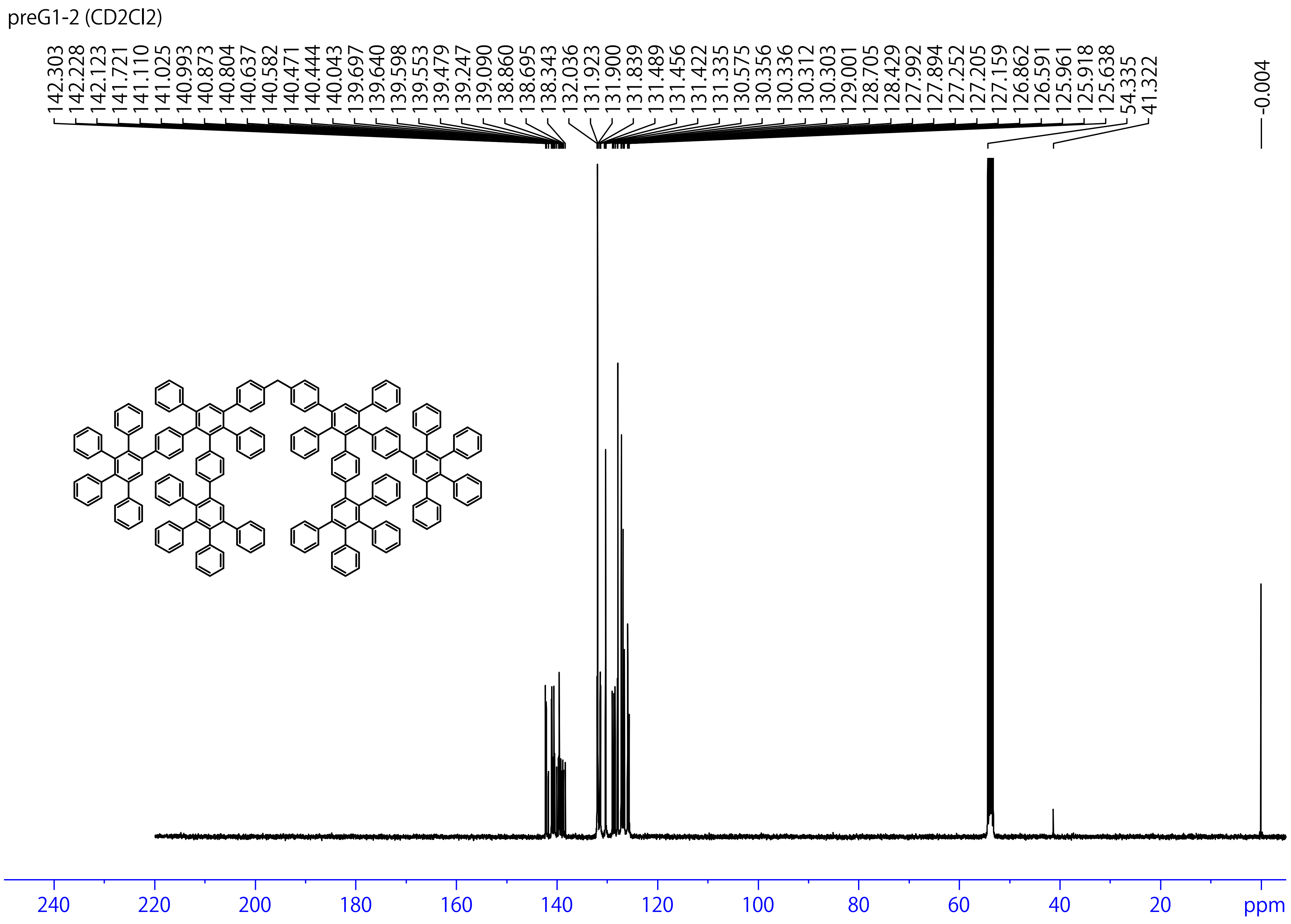

Fig C30. 13C-NMR spectra of compound 14.

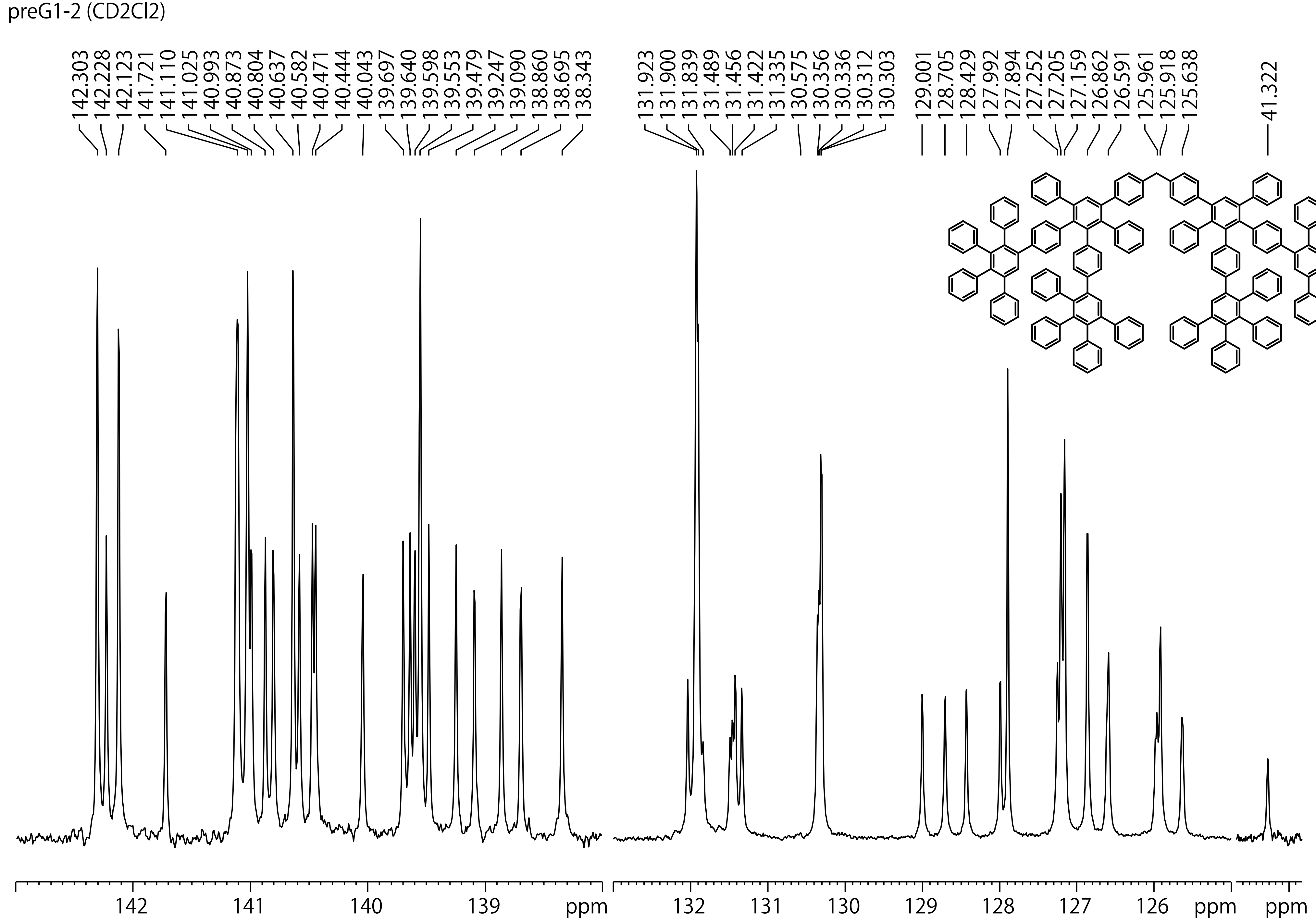

Fig C31. 13C-NMR spectra of compound 14.

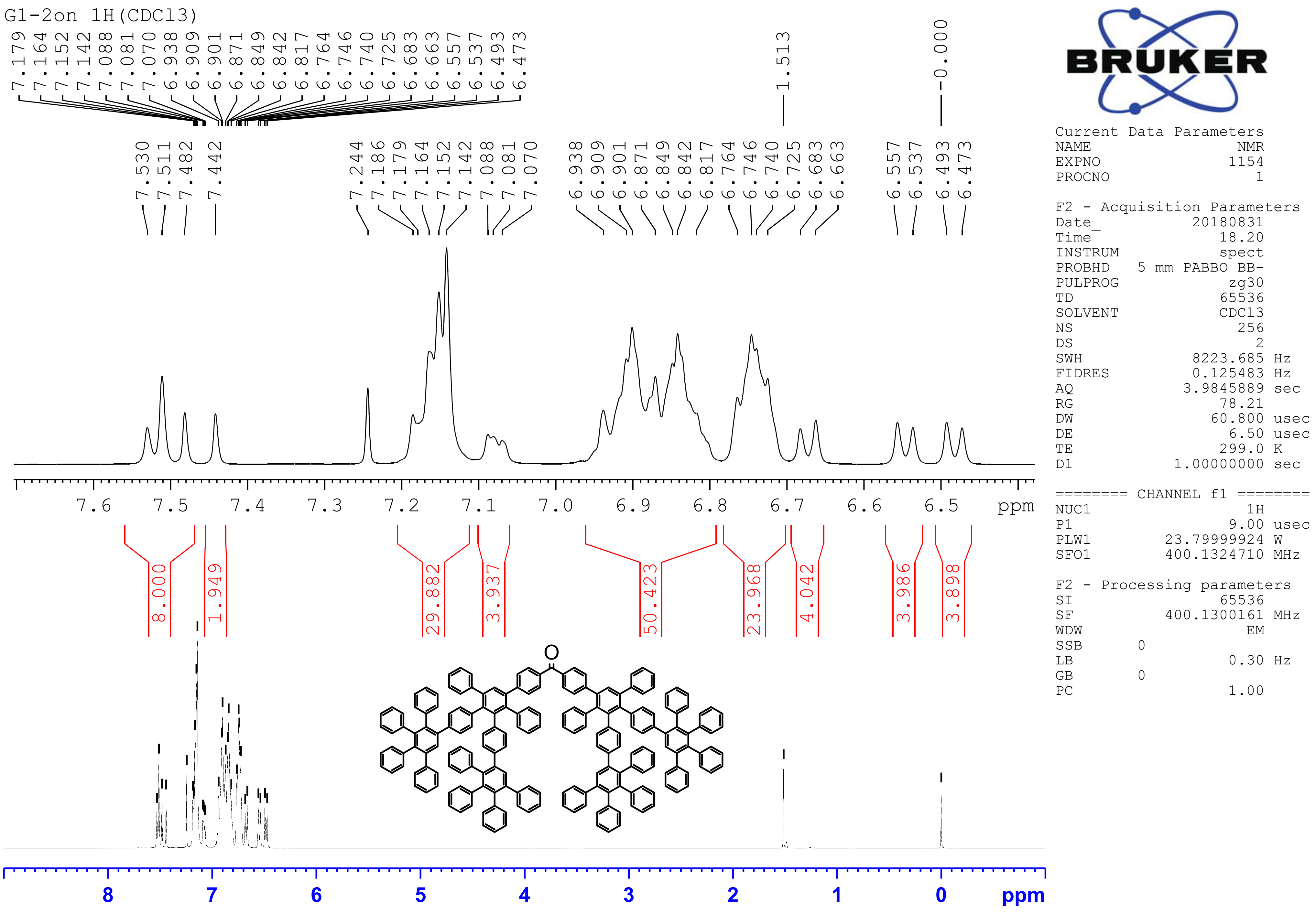

Fig C32. 1H-NMR spectra of compound 15.

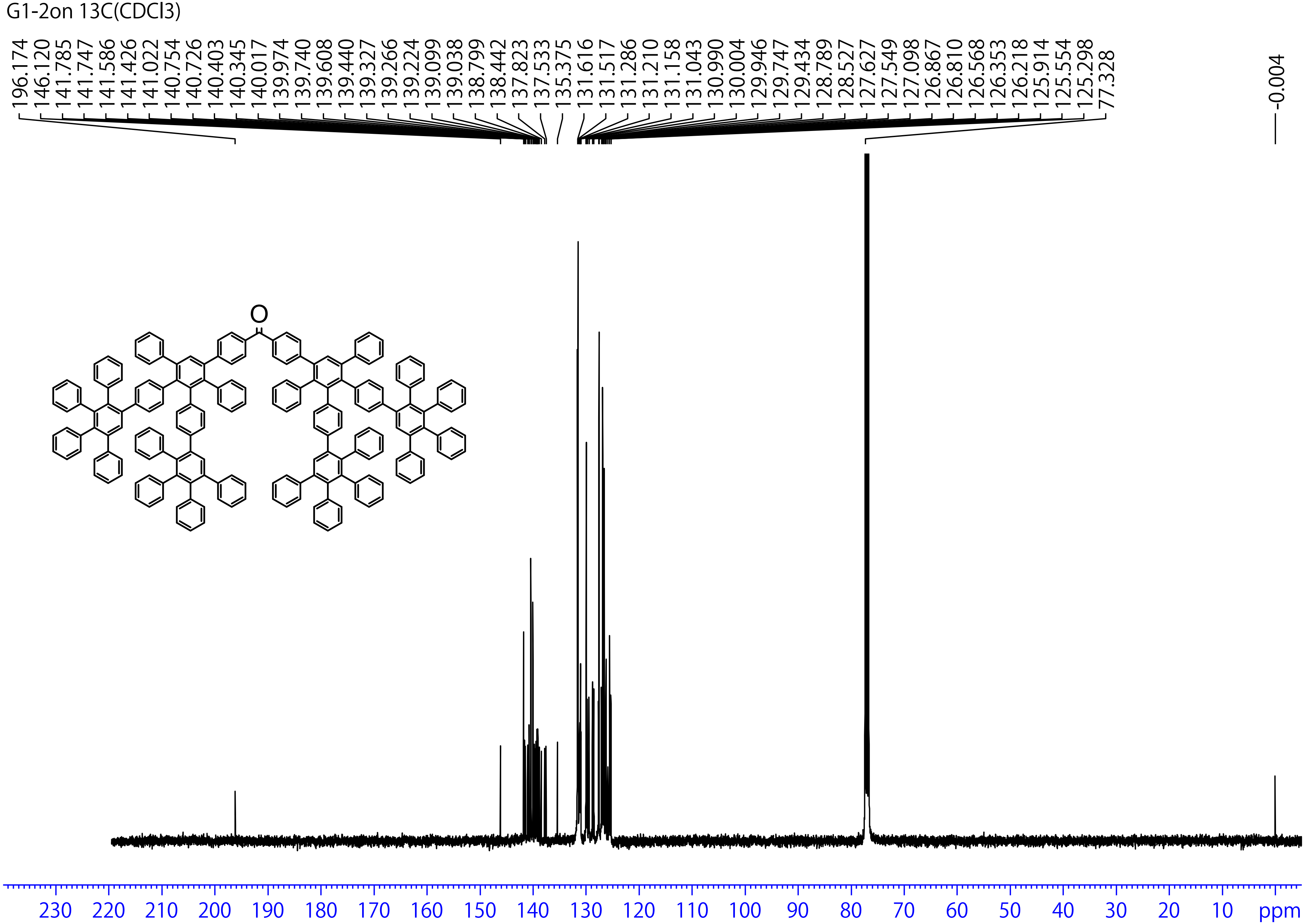

Fig C33. 13C-NMR spectra of compound 15.

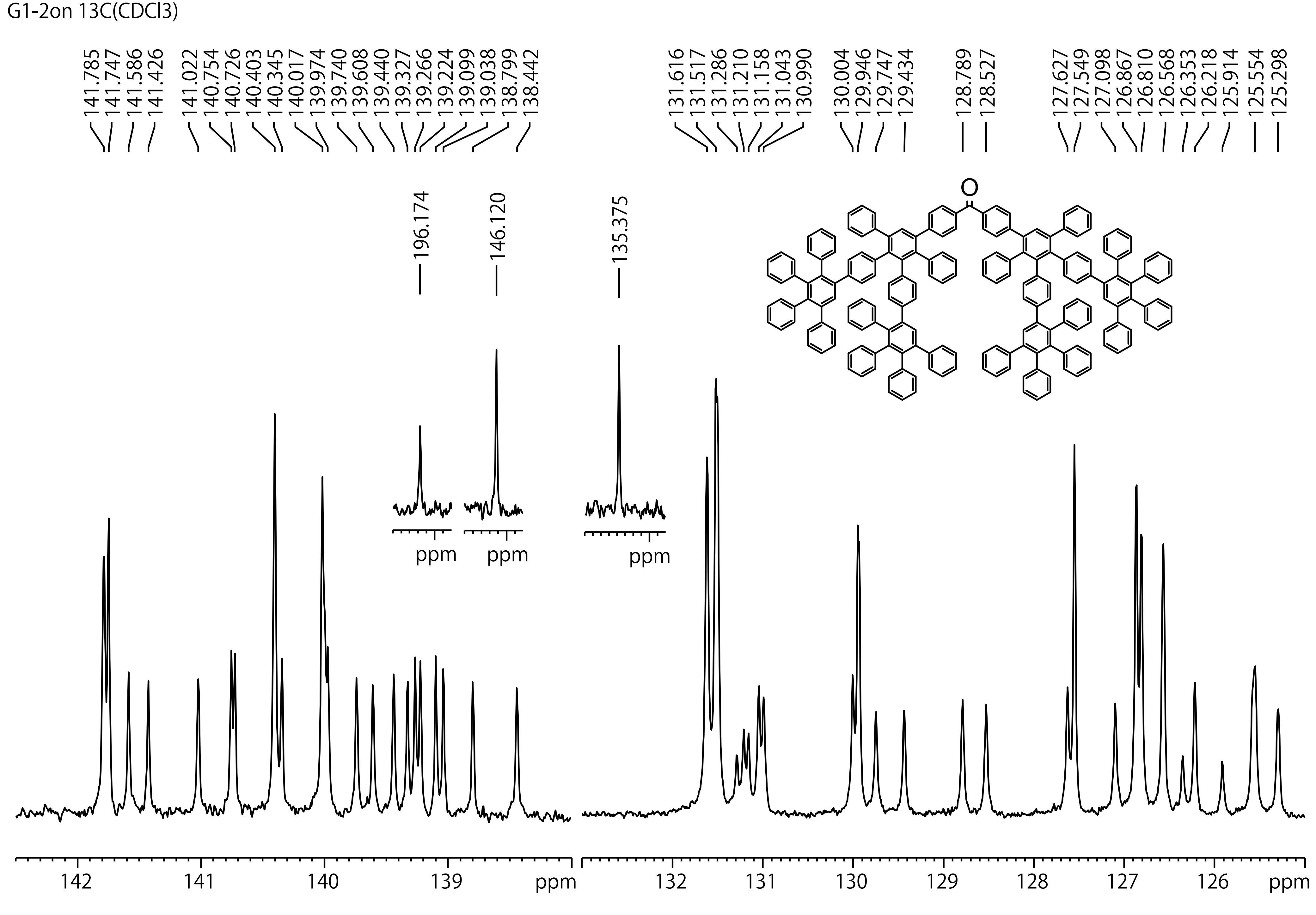

Fig C34. 13C-NMR spectra of compound 15.

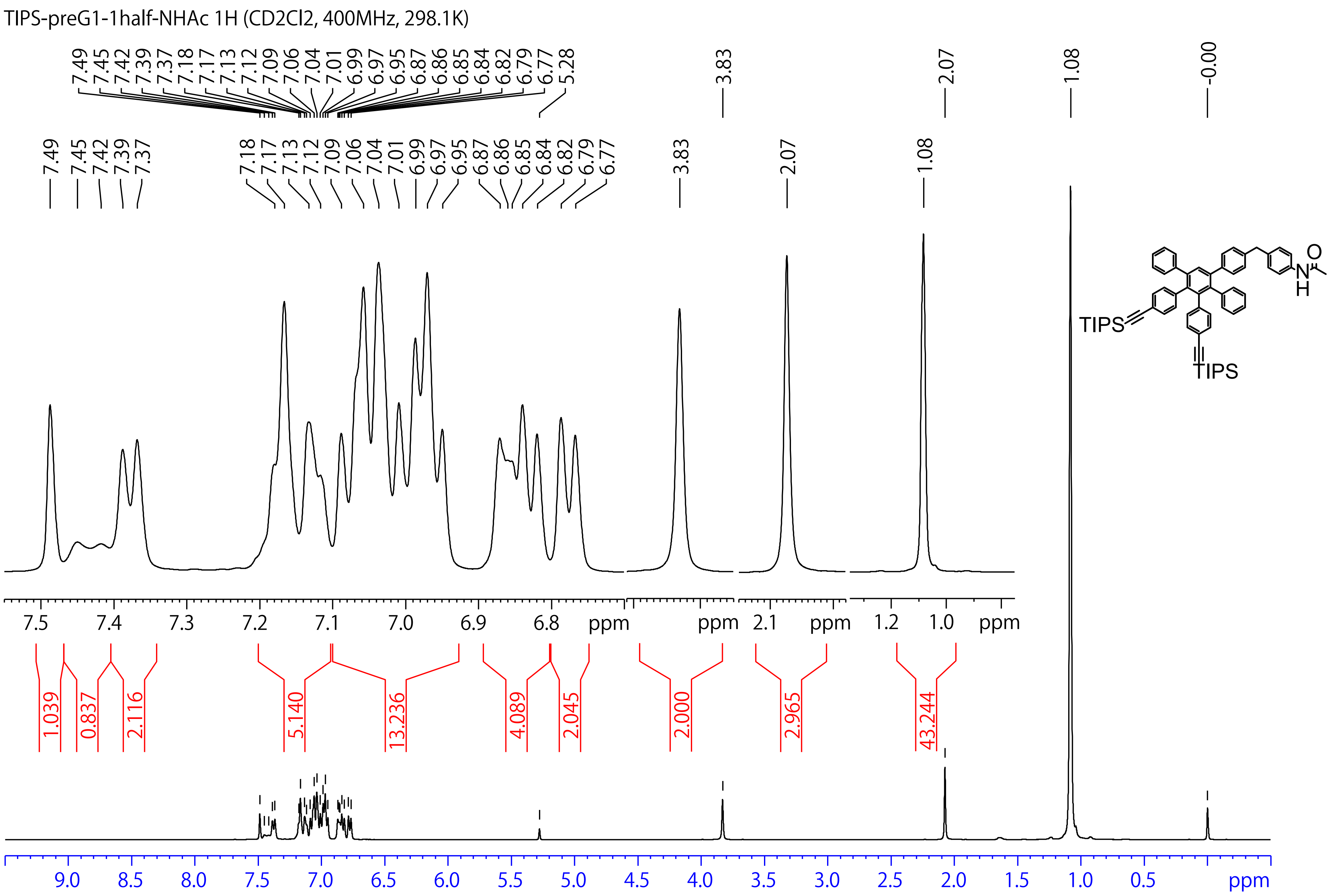

Fig C35. 1H-NMR spectra of compound 16.

TIPS-preG1-1half-NHAc 13C (CD2Cl2, 100MHz, 299.4K)

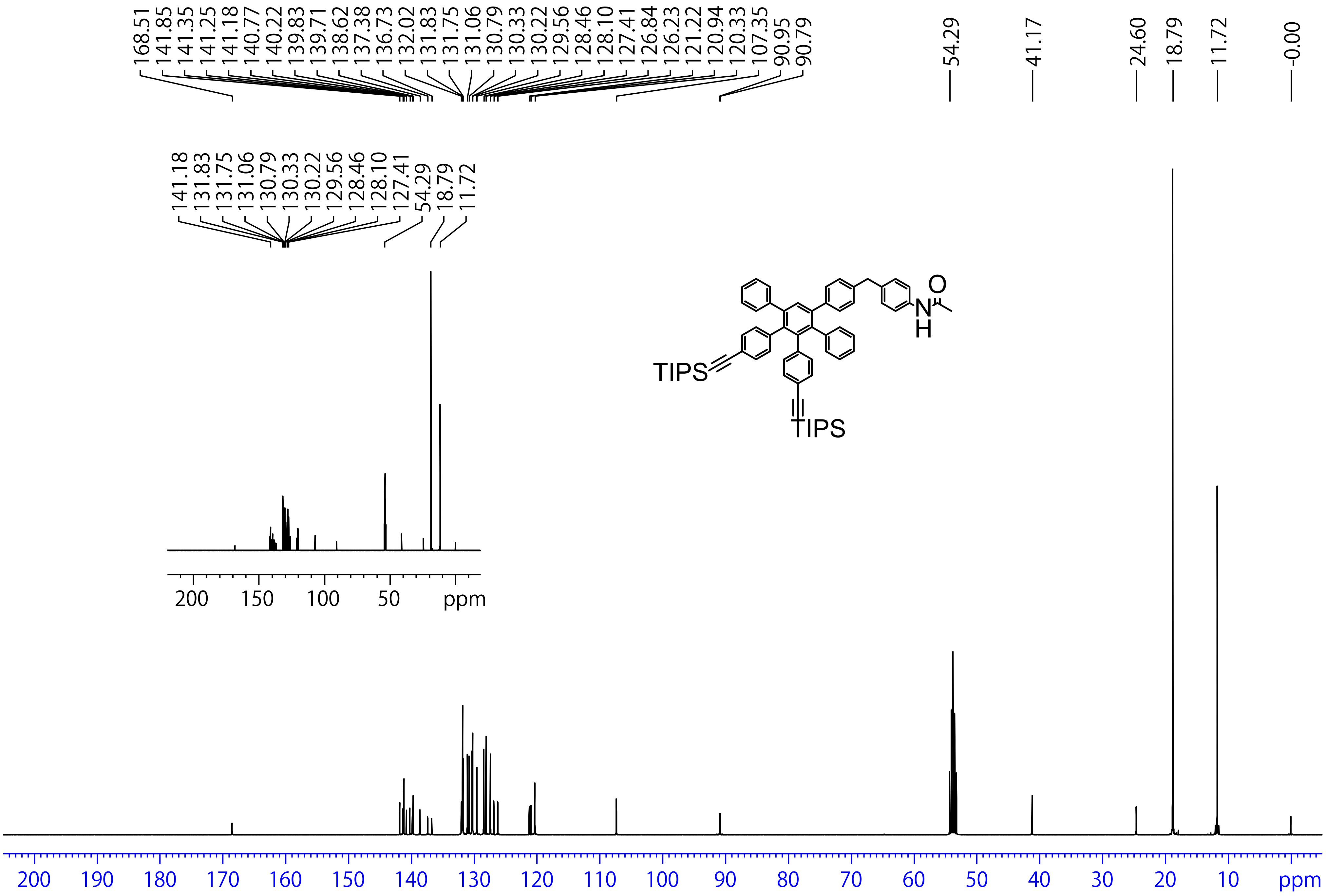

Fig C36. 13C-NMR spectra of compound 16.

Alkyn-preG1-1half-NHAc 1H (CD2Cl2, 400MHz, 298.2K)

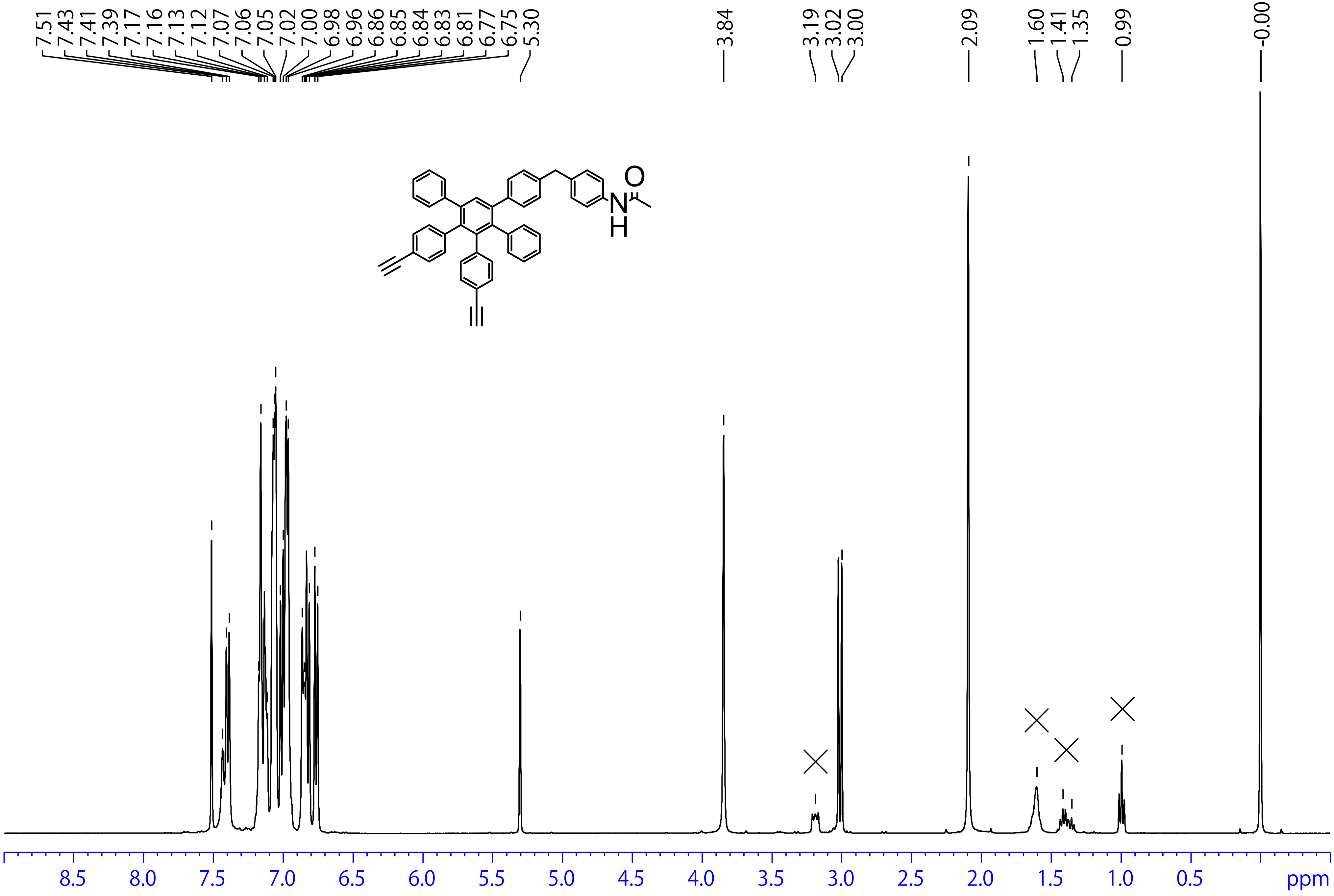

Fig C37. 1H-NMR spectra of compound 17.

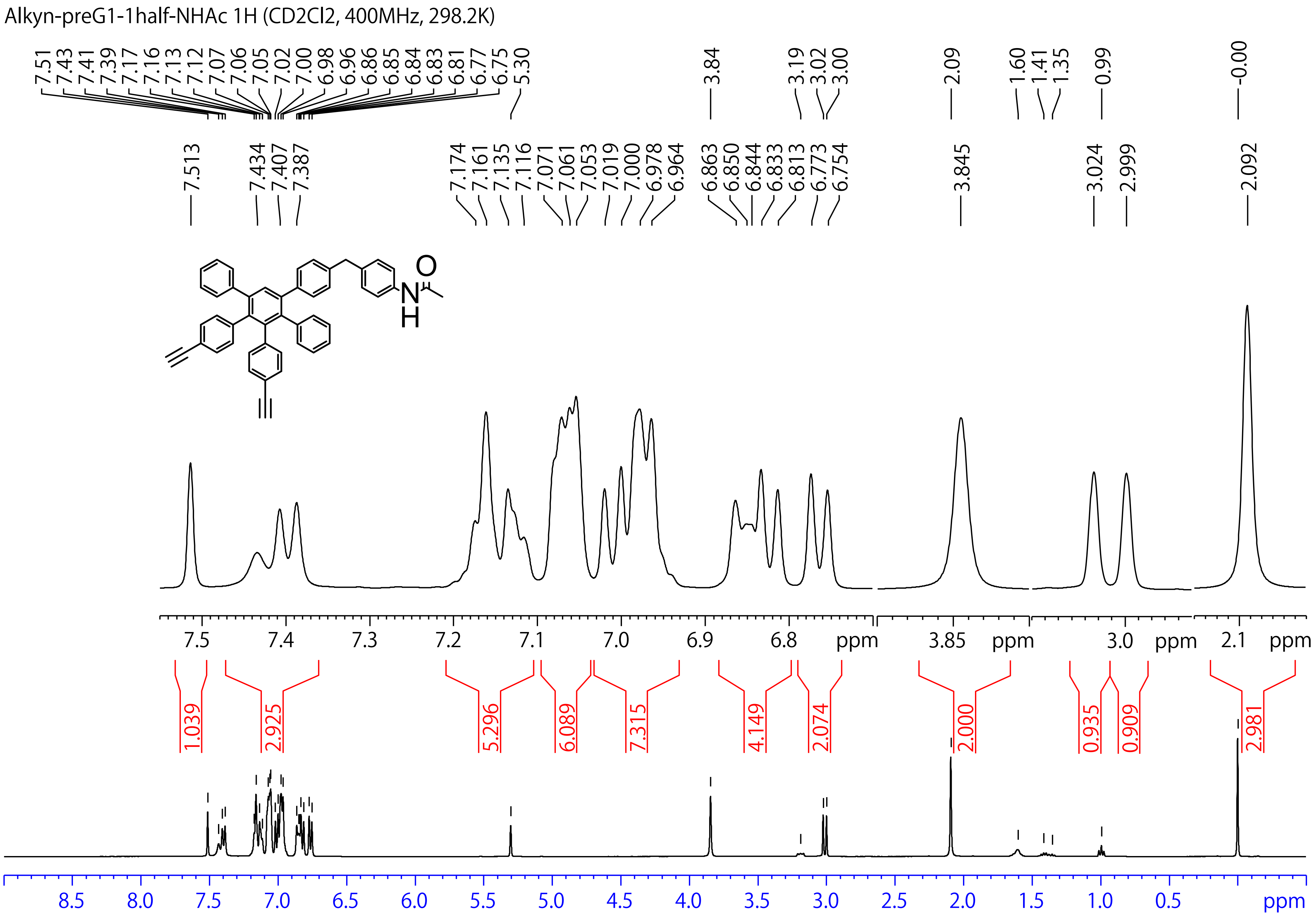

Fig C38. 1H-NMR spectra of compound 17.

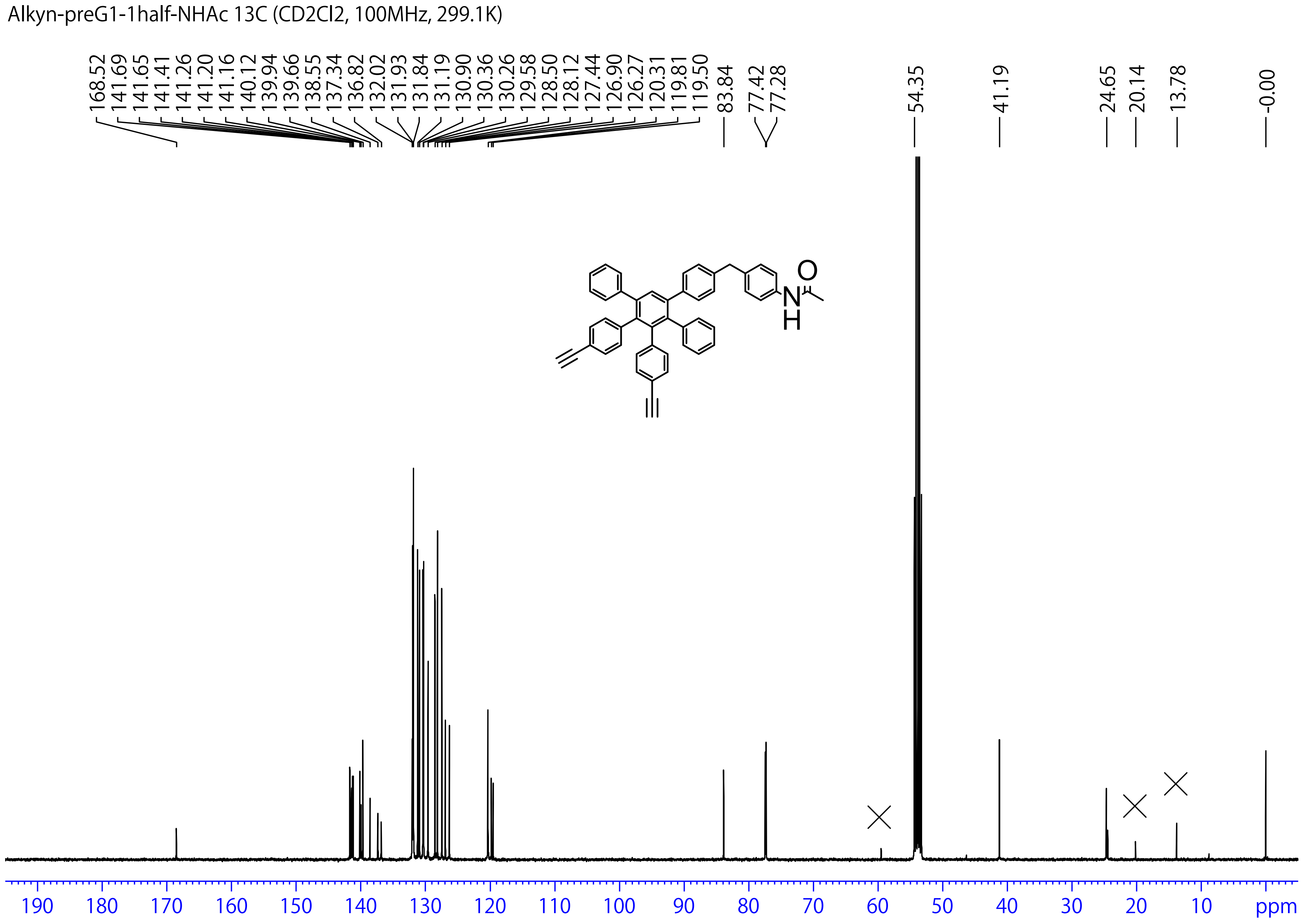

Fig C39. 13C-NMR spectra of compound 17.

preG1-2half-NHAc 1H(CDCl3)

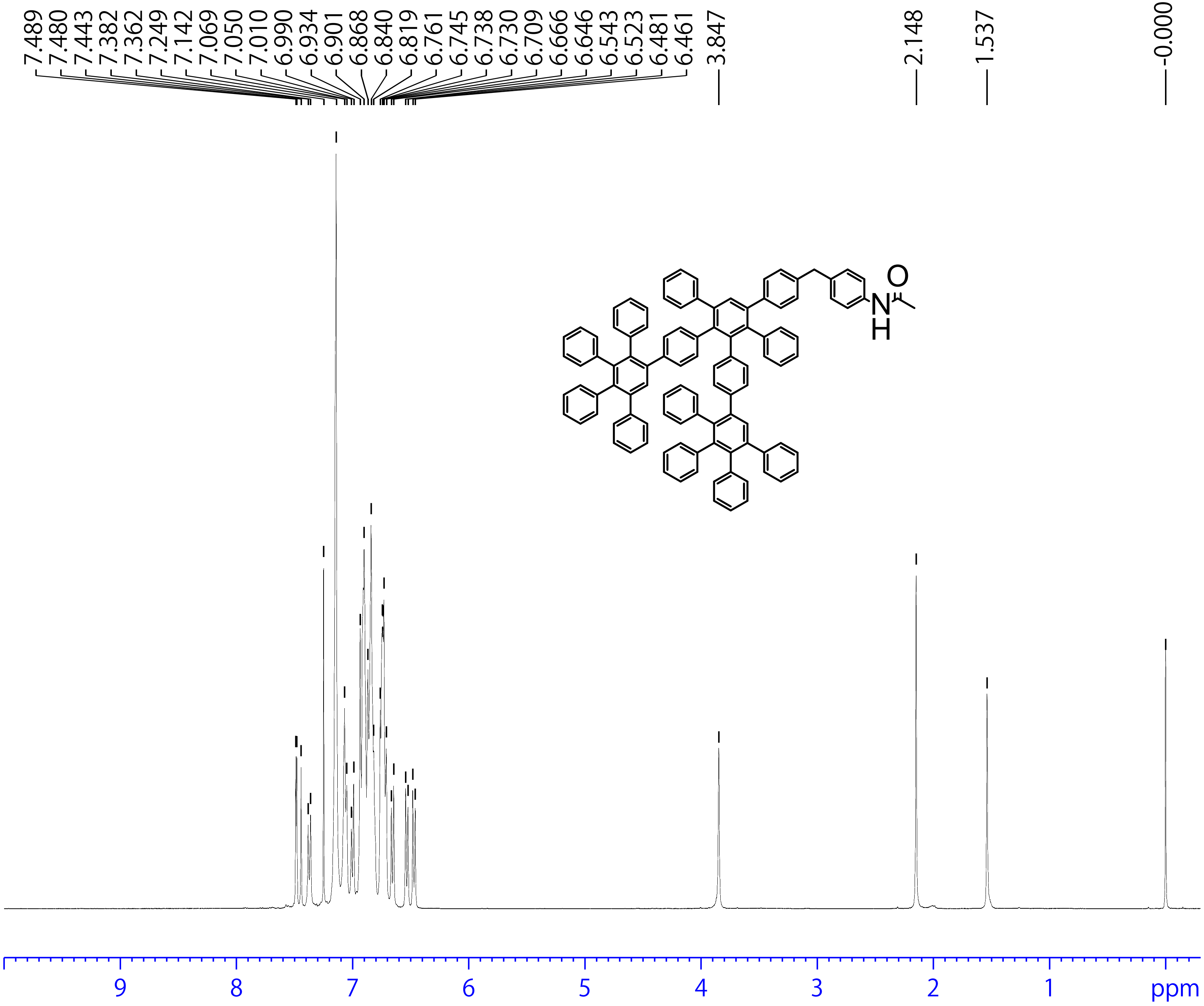

BRUKER

Current Data Parameters
NAME NMR
EXPNO 1191
PROCNO 1

F2 - Acquisition Parameters
Date_ 20180906
Time 5.25
INSTRUM spect
PROBHD 5 mm PABBO BB-
PULPROG zg30
TD 65536
SOLVENT CDCl3
NS 256
DS 2
SWH 8223.685 Hz
FIDRES 0.125483 Hz
AQ 3.9845889 sec
RG 85.96
DW 60.800 usec
DE 6.50 usec
TE 298.9 K
D1 1.00000000 sec

======== CHANNEL f1 ========
NUC1 1H
P1 9.00 usec
PLW1 23.79999924 W
SFO1 400.1324710 MHz

F2 - Processing parameters
SI 65536
SF 400.1300142 MHz
WDW EM
SSB 0
LB 0.30 Hz
GB 0
PC 1.00

Fig C40. 1H-NMR spectra of compound 18.

preG1-2half-NHAc 1H(CDCl3)

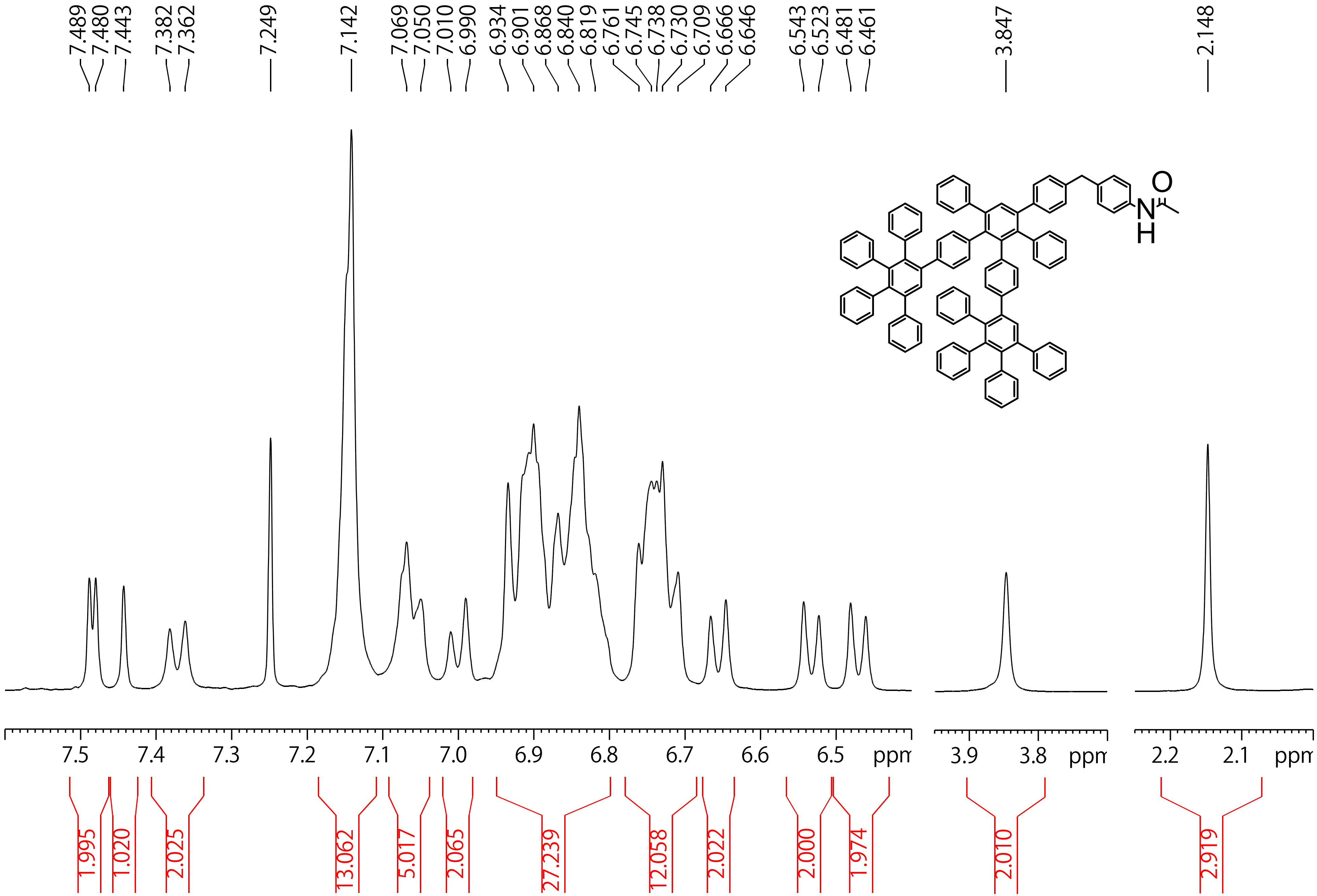

Fig C41. 1H-NMR spectra of compound 18.

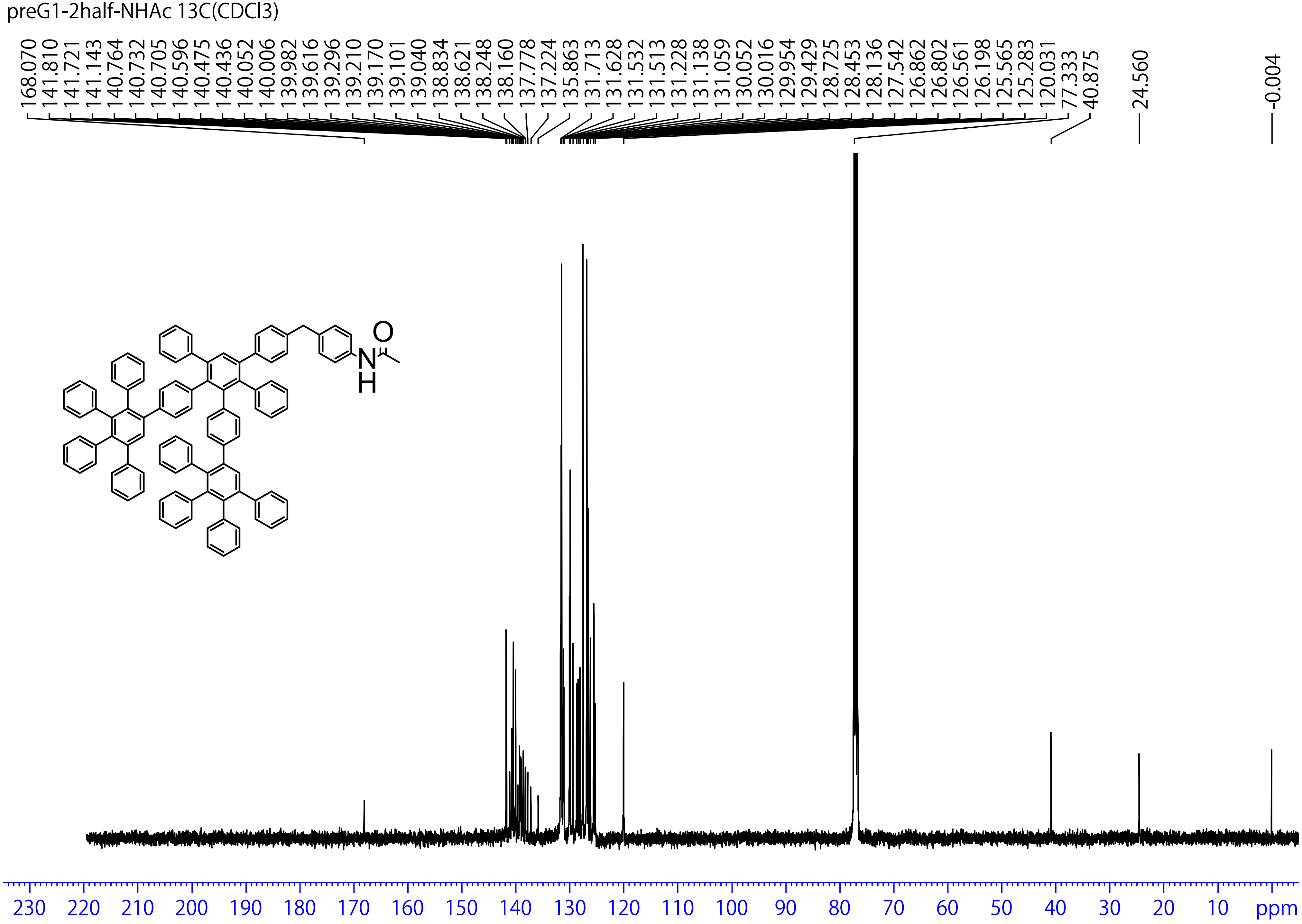

Fig C42. 13C-NMR spectra of compound 18.

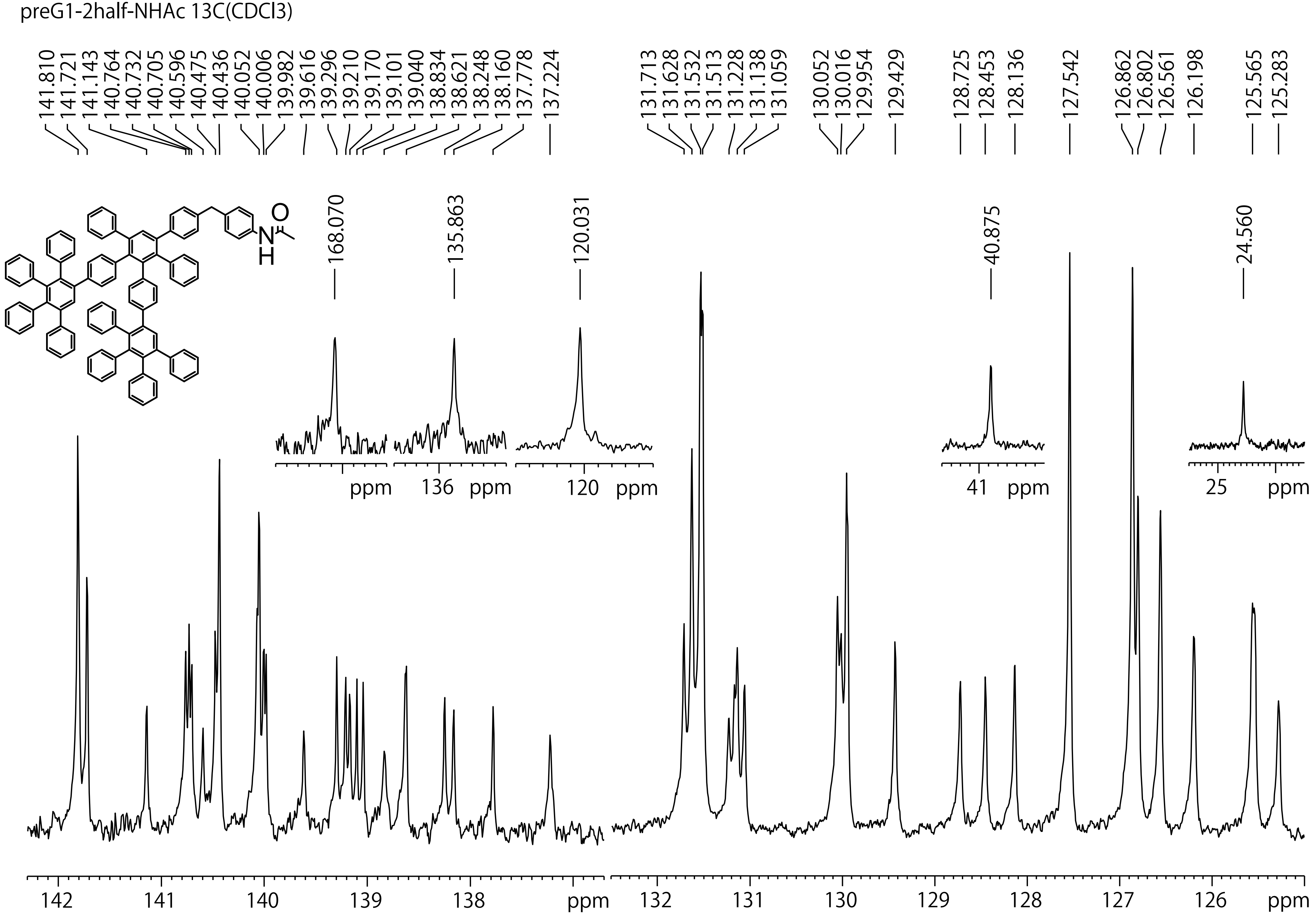

Fig C43. 13C-NMR spectra of compound 18.

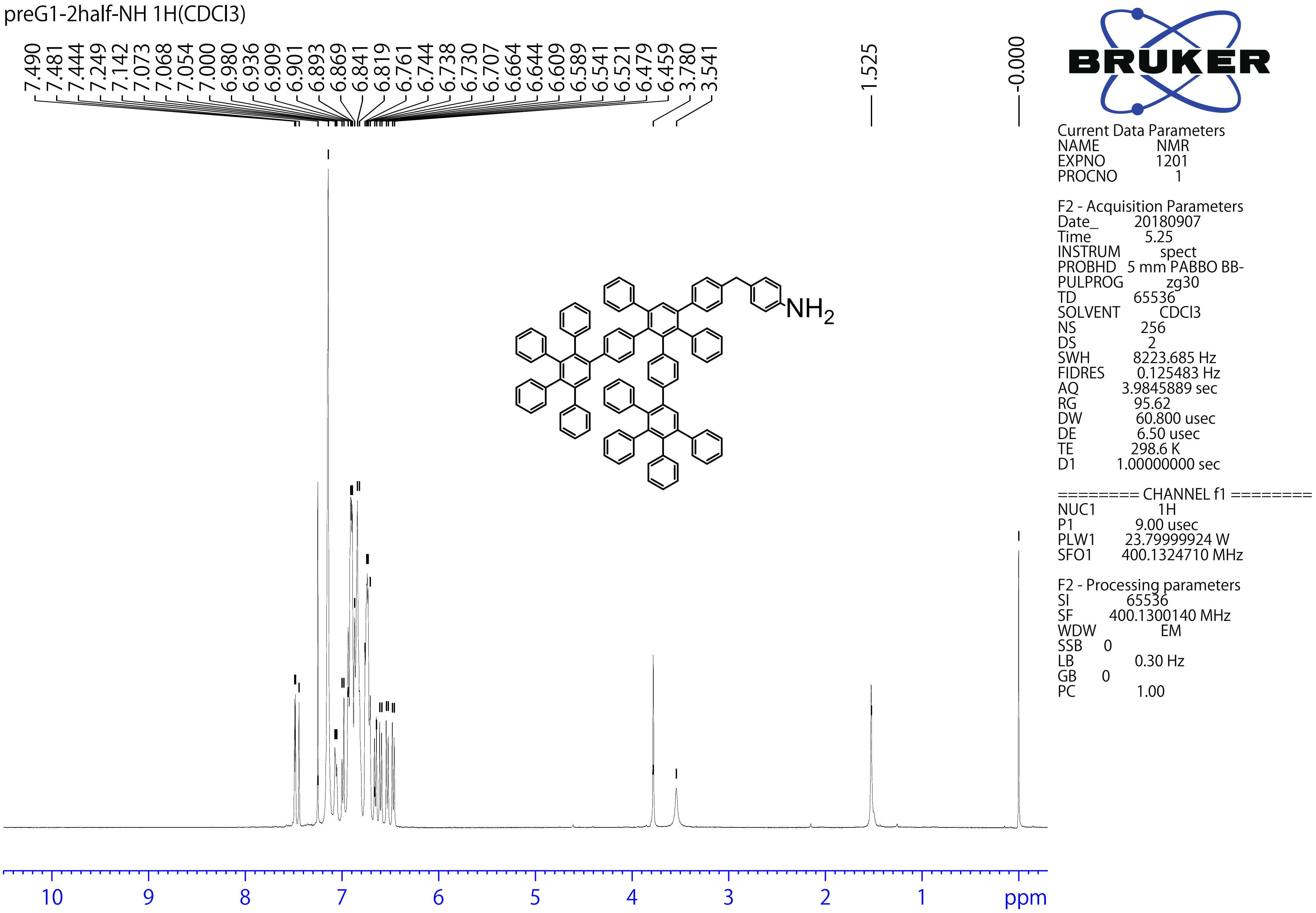

Fig C44. 1H-NMR spectra of compound 19.

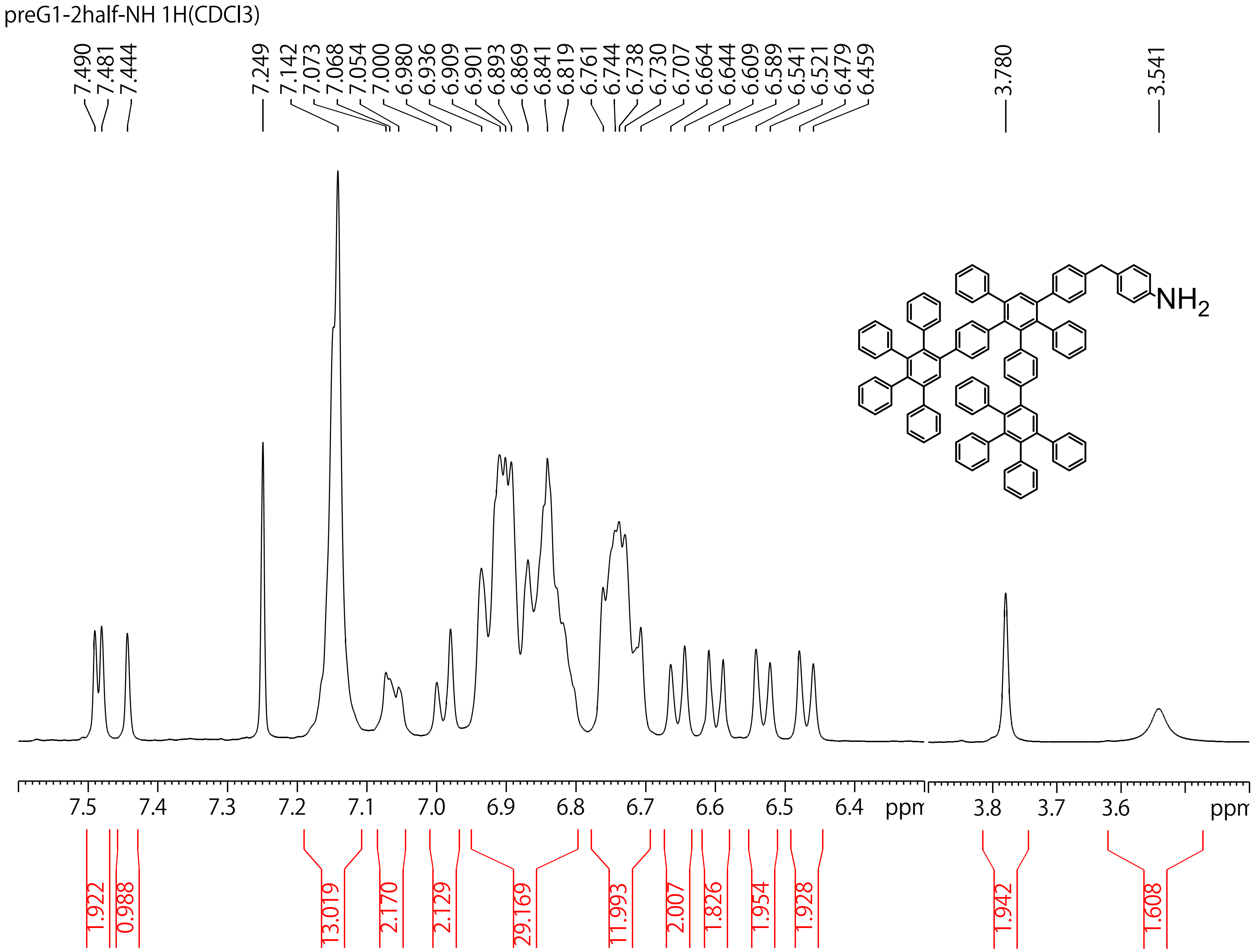

Fig C45. 1H-NMR spectra of compound 19.

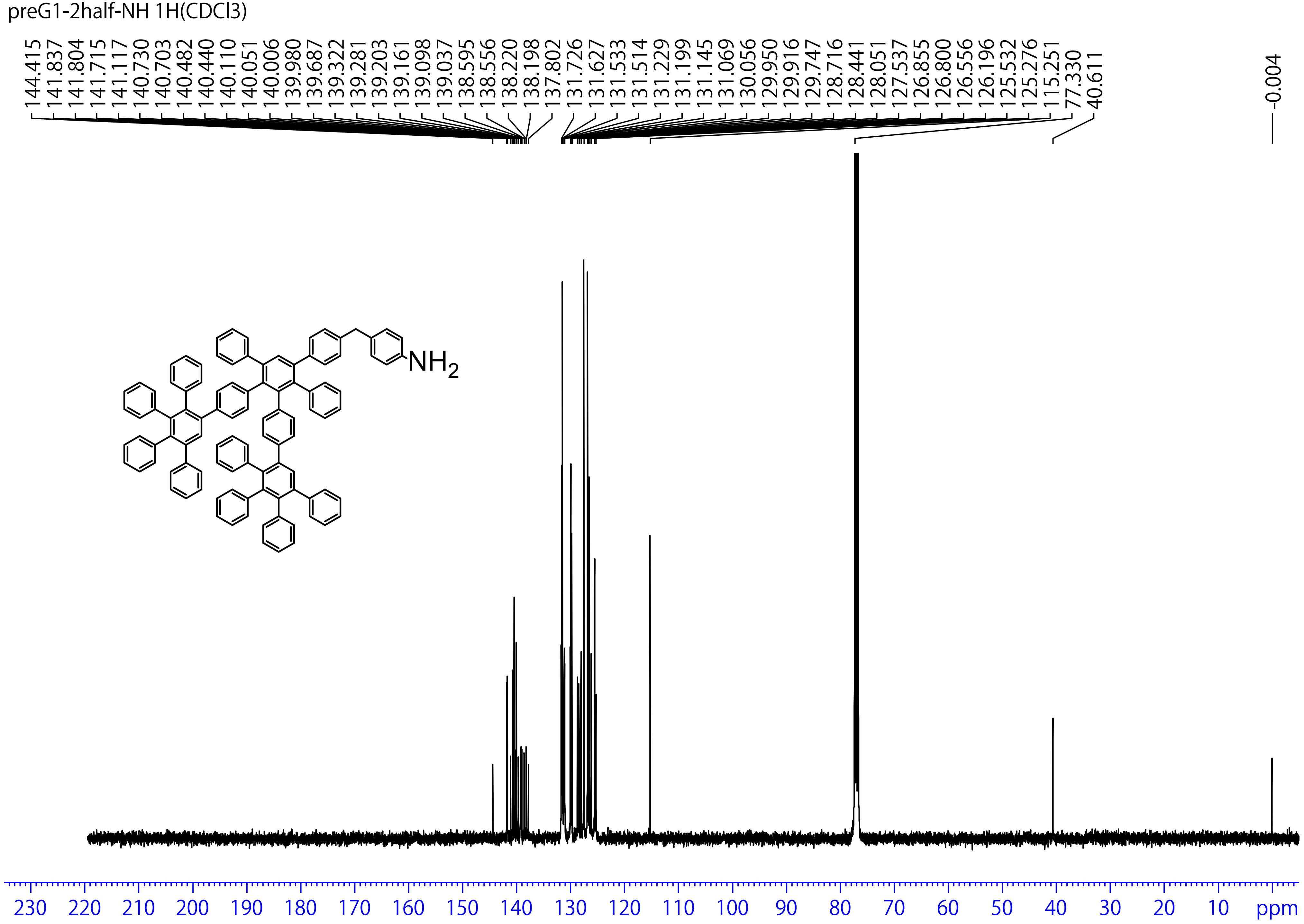

Fig C46. 13C-NMR spectra of compound 19.

preG1-2half-NH 1H(CDCl3)

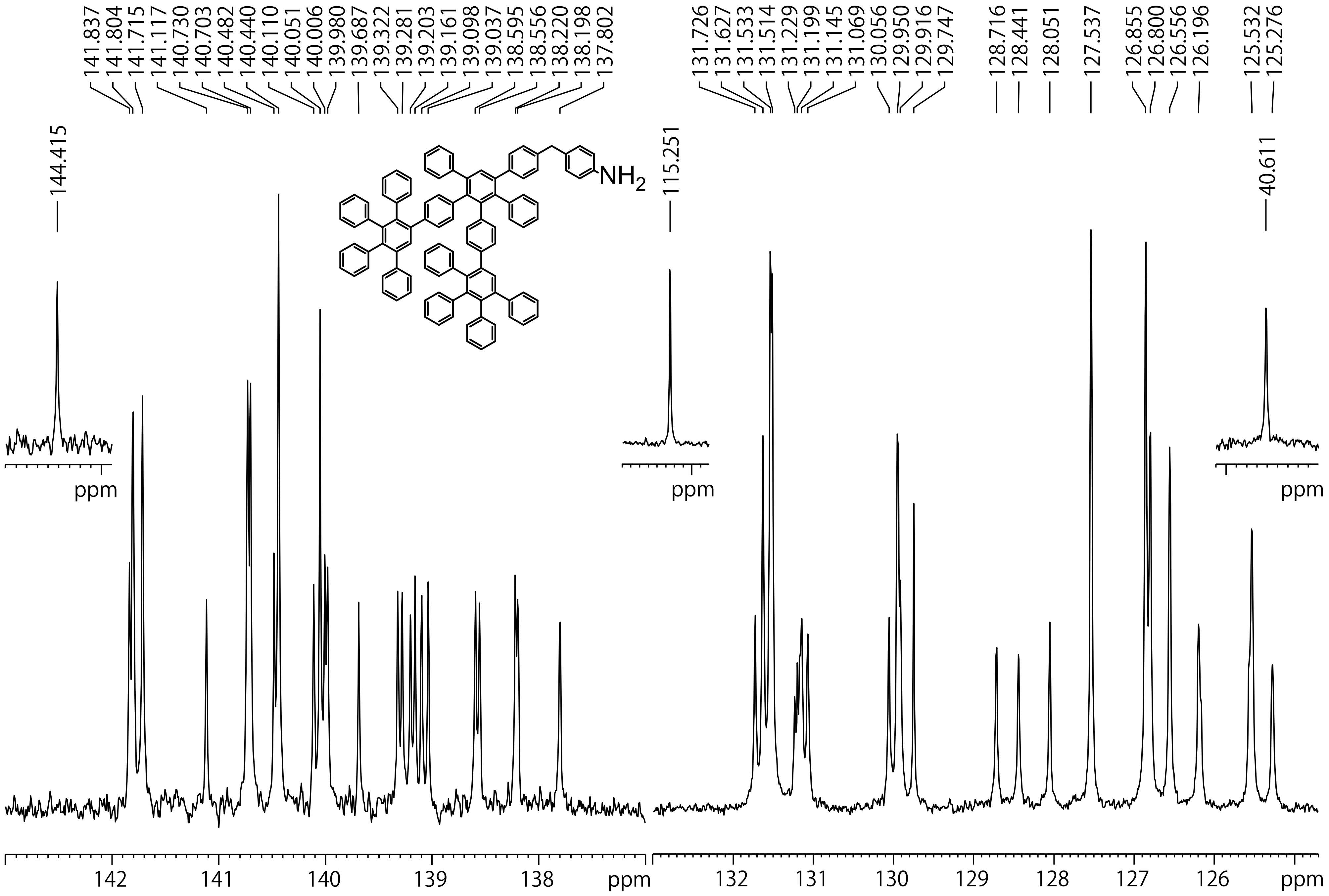

Fig C47. 13C-NMR spectra of compound 19.

[M+H] 1325.58

(22)

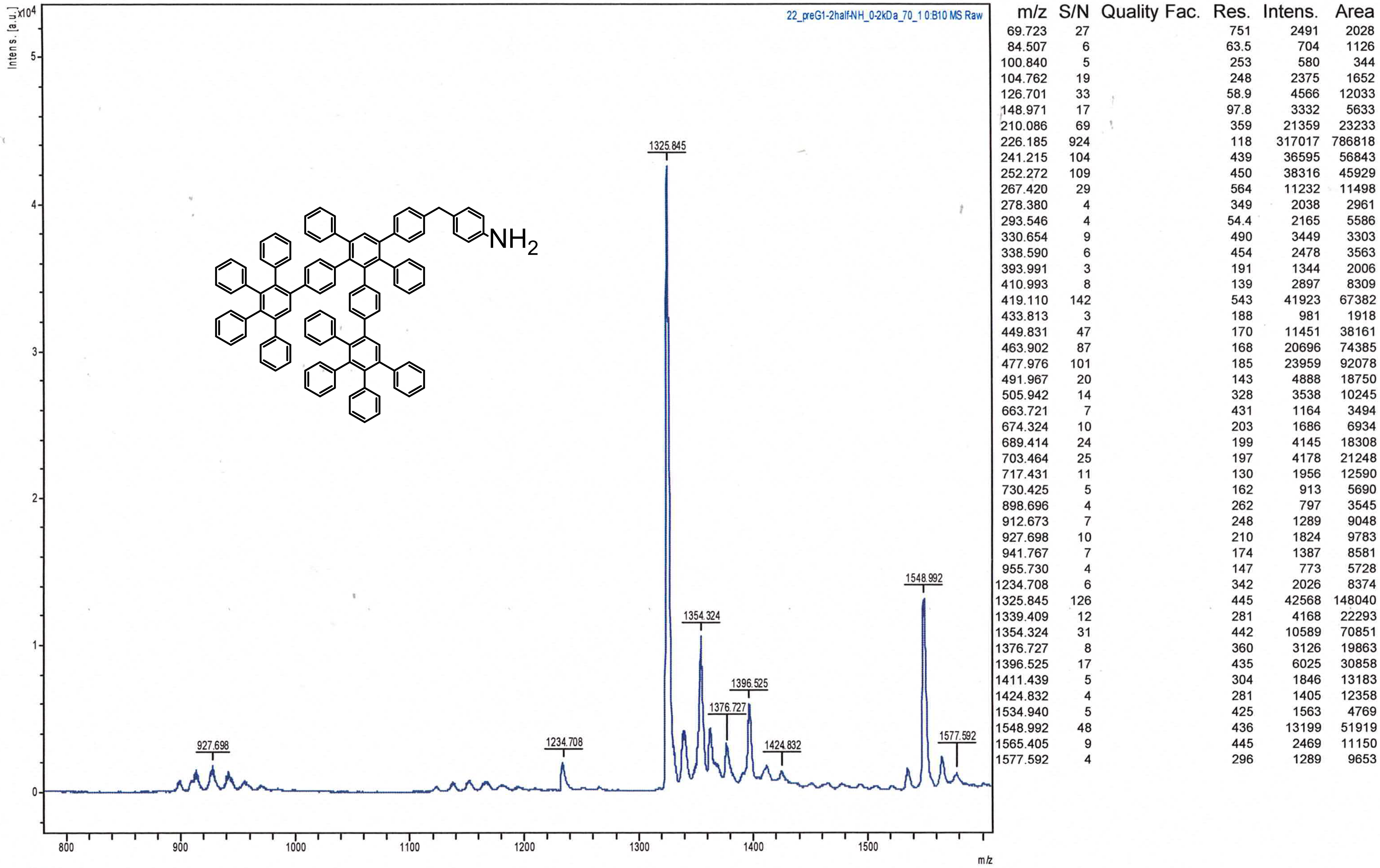

m/z	S/N	Quality Fac.	Res.	Intens.	Area
69.723	27		751	2491	2028
84.507	6		63.5	704	1126
100.840	5		253	580	344
104.762	19		248	2375	1652
126.701	33		58.9	4566	12033
148.971	17		97.8	3332	5633
210.086	69		359	21359	23233
226.185	924		118	317017	786818
241.215	104		439	36595	56843
252.272	109		450	38316	45929
267.420	29		564	11232	11498
278.380	4		349	2038	2961
293.546	4		54.4	2165	5586
330.654	9		490	3449	3303
338.590	6		454	2478	3563
393.991	3		191	1344	2006
410.993	8		139	2897	8309
419.110	142		543	41923	67382
433.813	3		188	981	1918
449.831	47		170	11451	38161
463.902	87		168	20696	74385
477.976	101		185	23959	92078
491.967	20		143	4888	18750
505.942	14		328	3538	10245
663.721	7		431	1164	3494
674.324	10		203	1686	6934
689.414	24		199	4145	18308
703.464	25		197	4178	21248
717.431	11		130	1956	12590
730.425	5		162	913	5690
898.696	4		262	797	3545
912.673	7		248	1289	9048
927.698	10		210	1824	9783
941.767	7		174	1387	8581
955.730	4		147	773	5728
1234.708	6		342	2026	8374
1325.845	126		445	42568	148040
1339.409	12		281	4168	22293
1354.324	31		442	10589	70851
1376.727	8		360	3126	19863
1396.525	17		435	6025	30858
1411.439	5		304	1846	13183
1424.832	4		281	1405	12358
1534.940	5		425	1563	4769
1548.992	48		436	13199	51919
1565.405	9		445	2469	11150
1577.592	4		296	1289	9653

11/29/2018 4:55:21 PM

Fig C48. MALDI-TOF-MS spectra of compound 19.

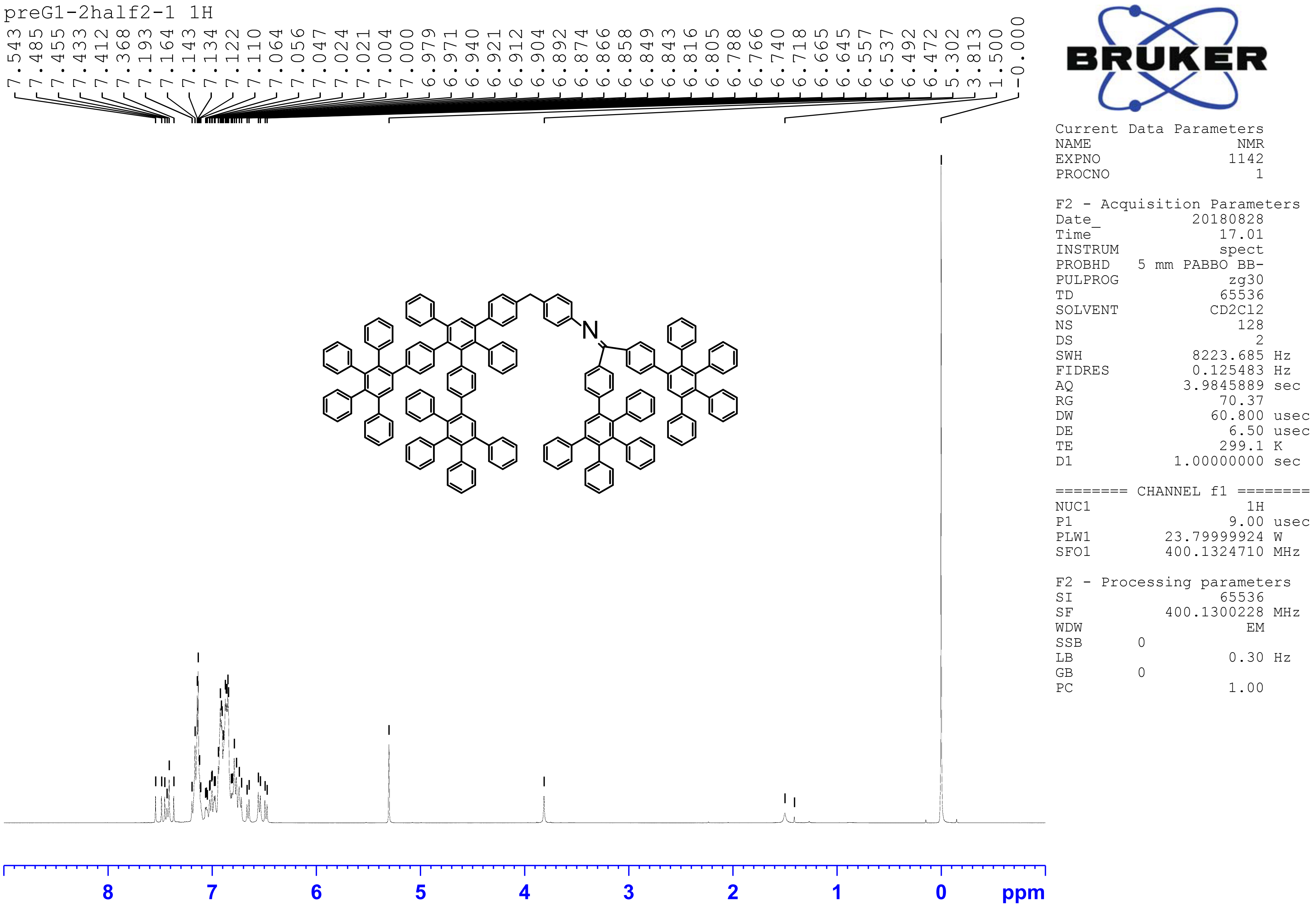

Fig C49. 1H-NMR spectra of compound 20.

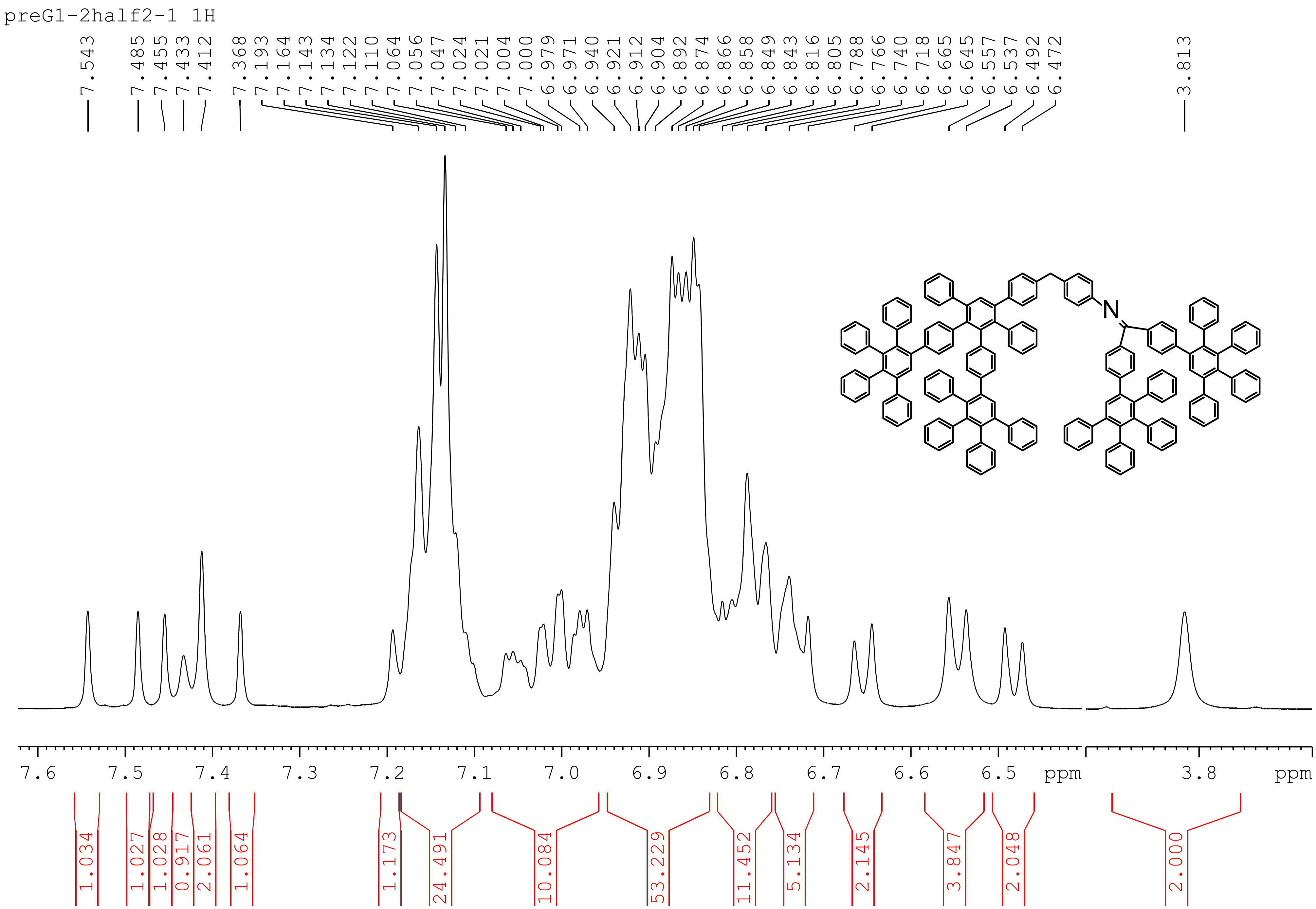

Fig C50. 1H-NMR spectra of compound 20.

[M+H] 2249.96

(23)

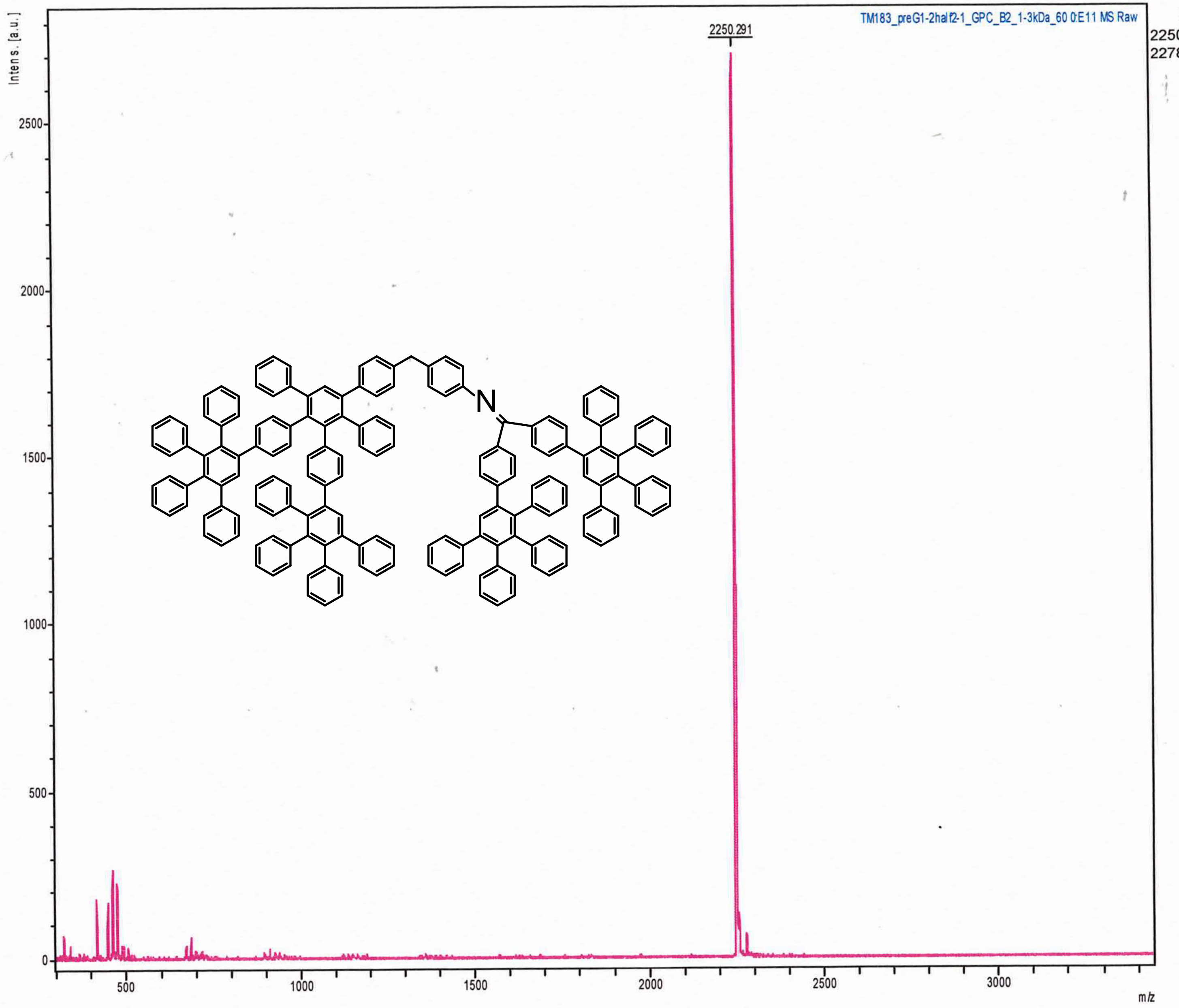

m/z	S/N	Quality Fac.	Res.	Intens.	Area
2250.291	373		695	2707	10546
2278.842	8		792	59.0	297

11/30/2018 2:33:44 PM

Fig C51. MALDI-TOF-MS spectra of compound 20.

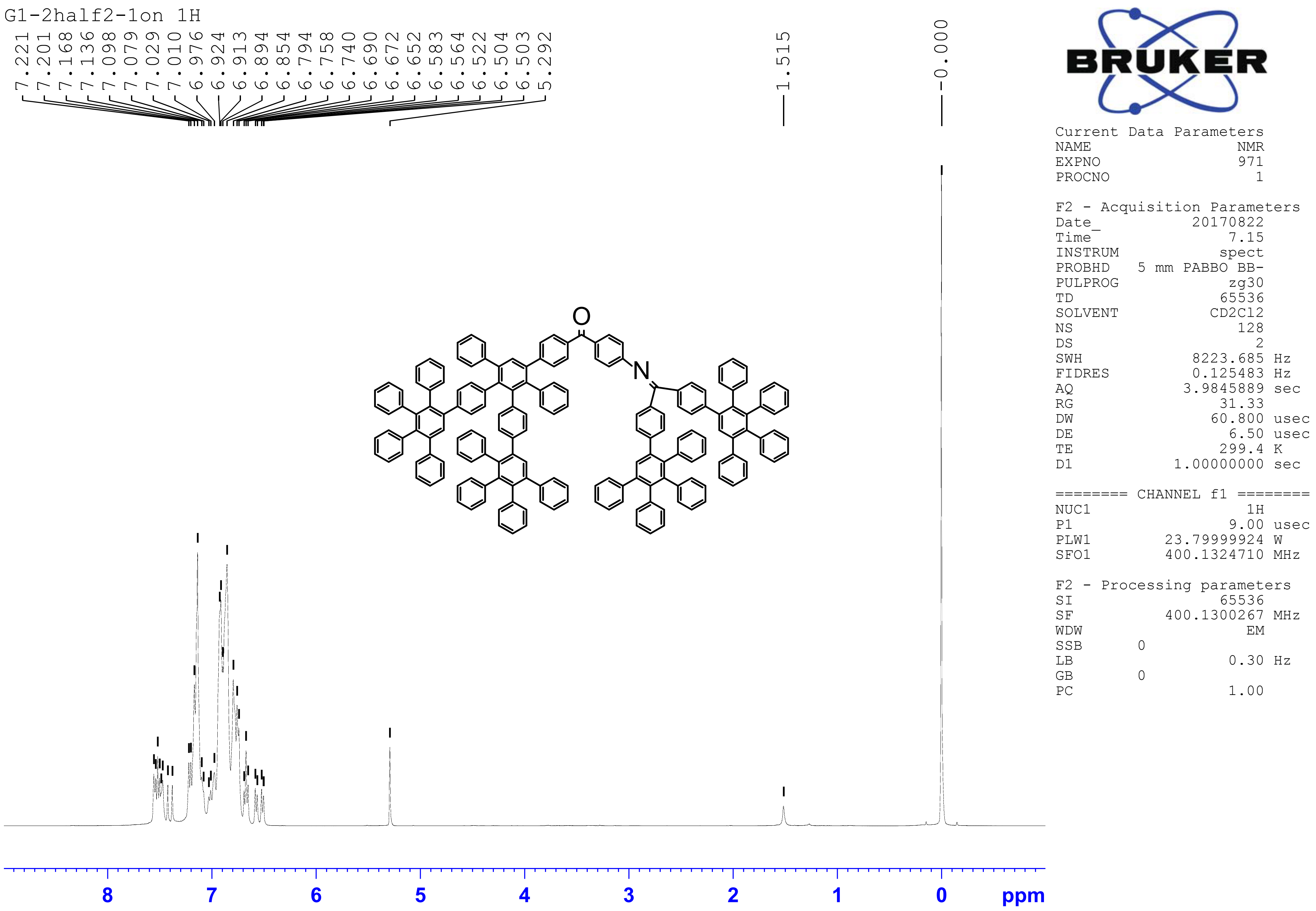

Fig C52. 1H-NMR spectra of compound 21.

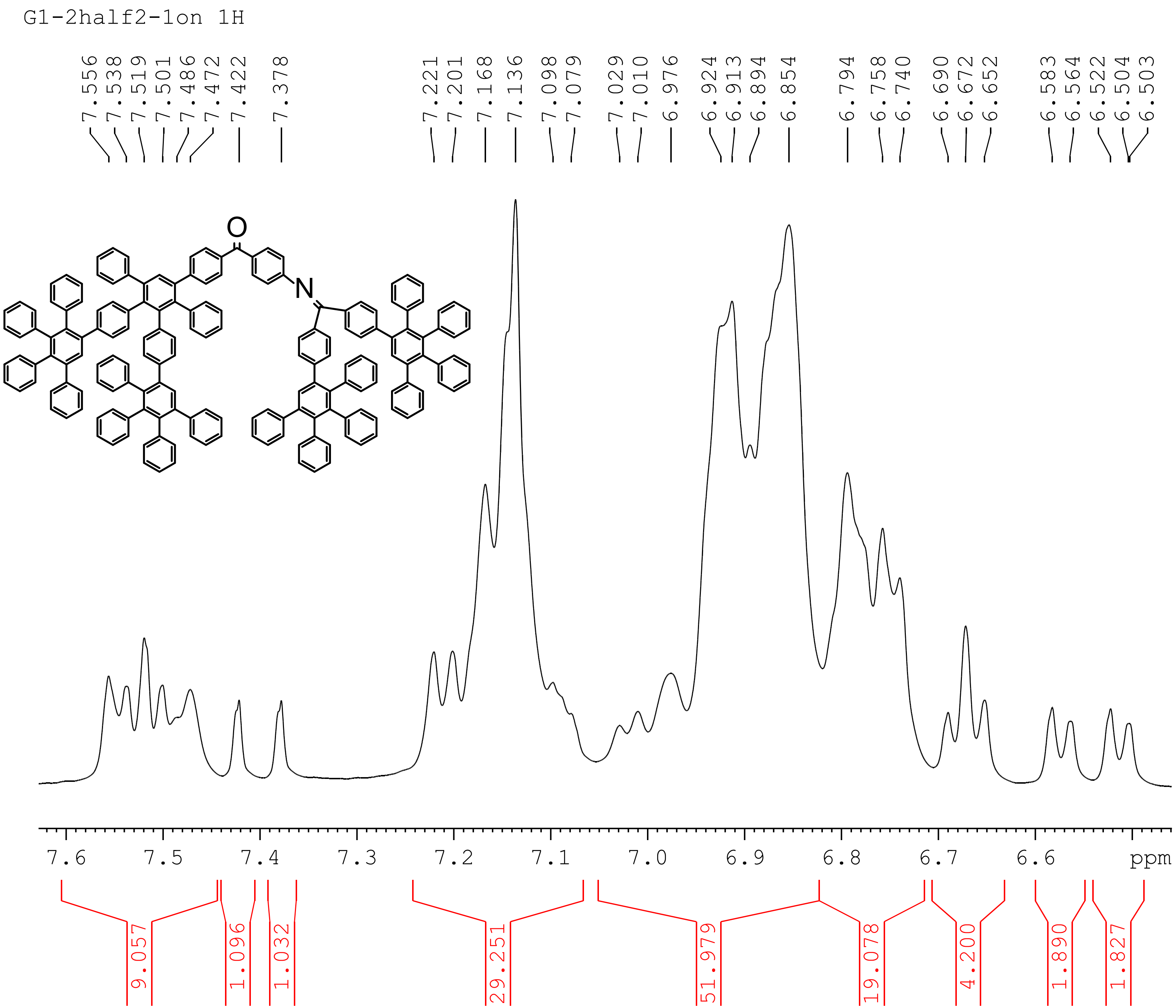


```
Current Data Parameters
NAME                NMR
EXPNO               971
PROCNO                1

F2 - Acquisition Parameters
Date_          20170822
Time               7.15
INSTRUM           spect
PROBHD   5 mm PABBO BB-
PULPROG            zg30
TD                65536
SOLVENT          CD2Cl2
NS                  128
DS                    2
SWH            8223.685 Hz
FIDRES         0.125483 Hz
AQ            3.9845889 sec
RG                31.33
DW               60.800 usec
DE                 6.50 usec
TE                299.4 K
D1           1.00000000 sec

======== CHANNEL f1 ========
NUC1                 1H
P1                 9.00 usec
PLW1        23.79999924 W
SFO1        400.1324710 MHz

F2 - Processing parameters
SI                65536
SF          400.1300267 MHz
WDW                  EM
SSB      0
LB                 0.30 Hz
GB       0
PC                 1.00
```

Fig C53. 1H-NMR spectra of compound 21.

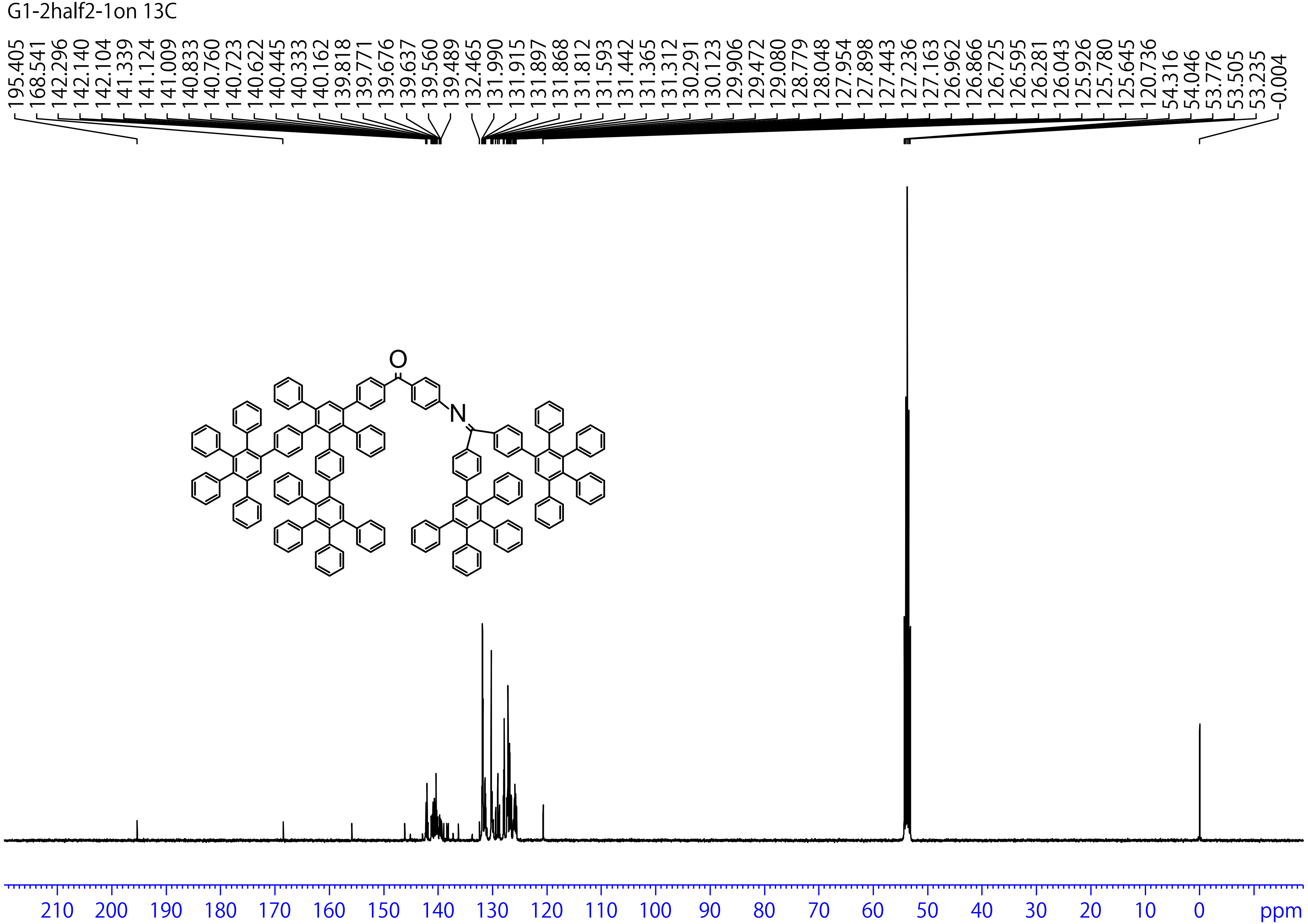

Fig C54. 13C-NMR spectra of compound 21.

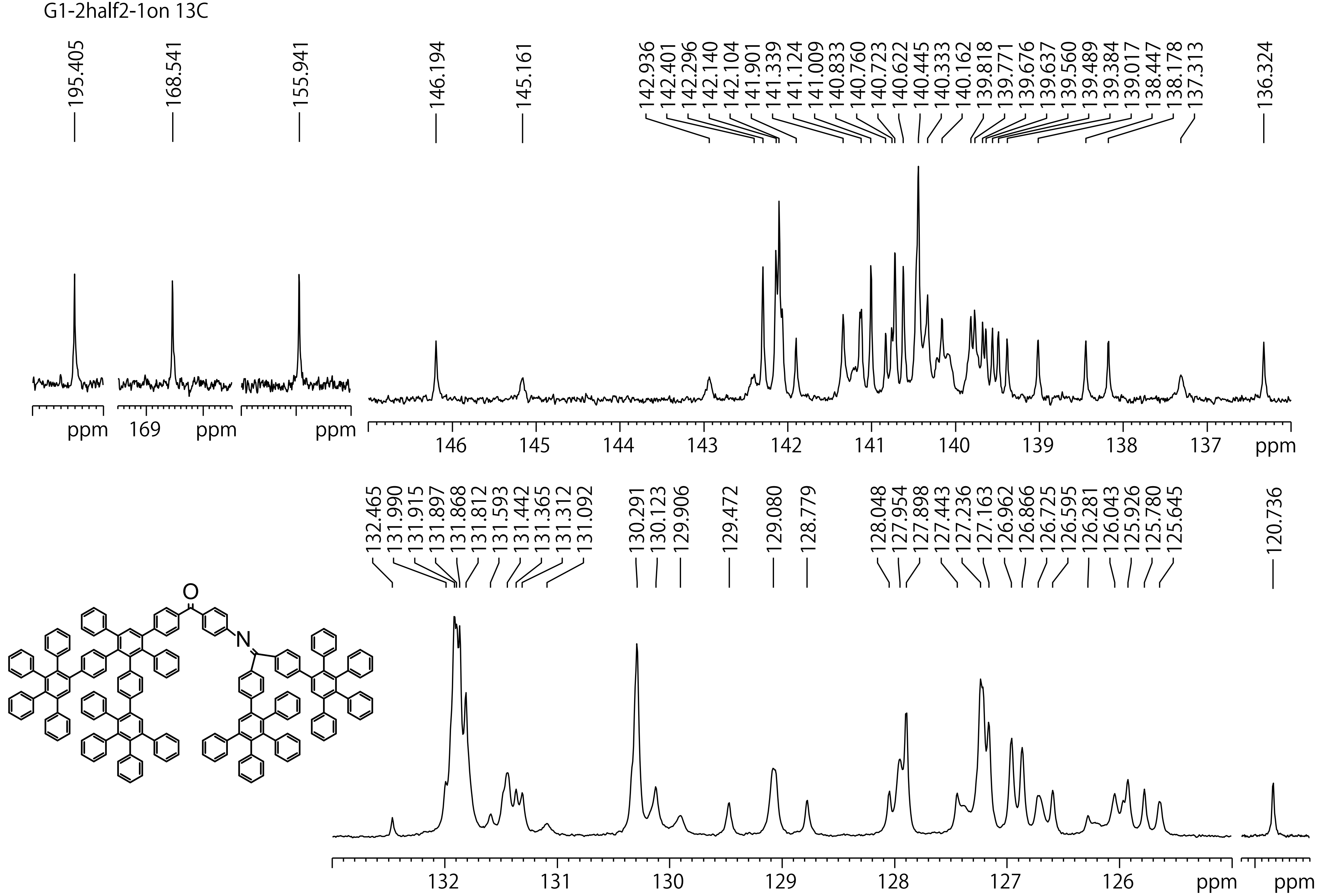

Fig C55. 13C-NMR spectra of compound 21.

[M+H] 2263.94 (24)

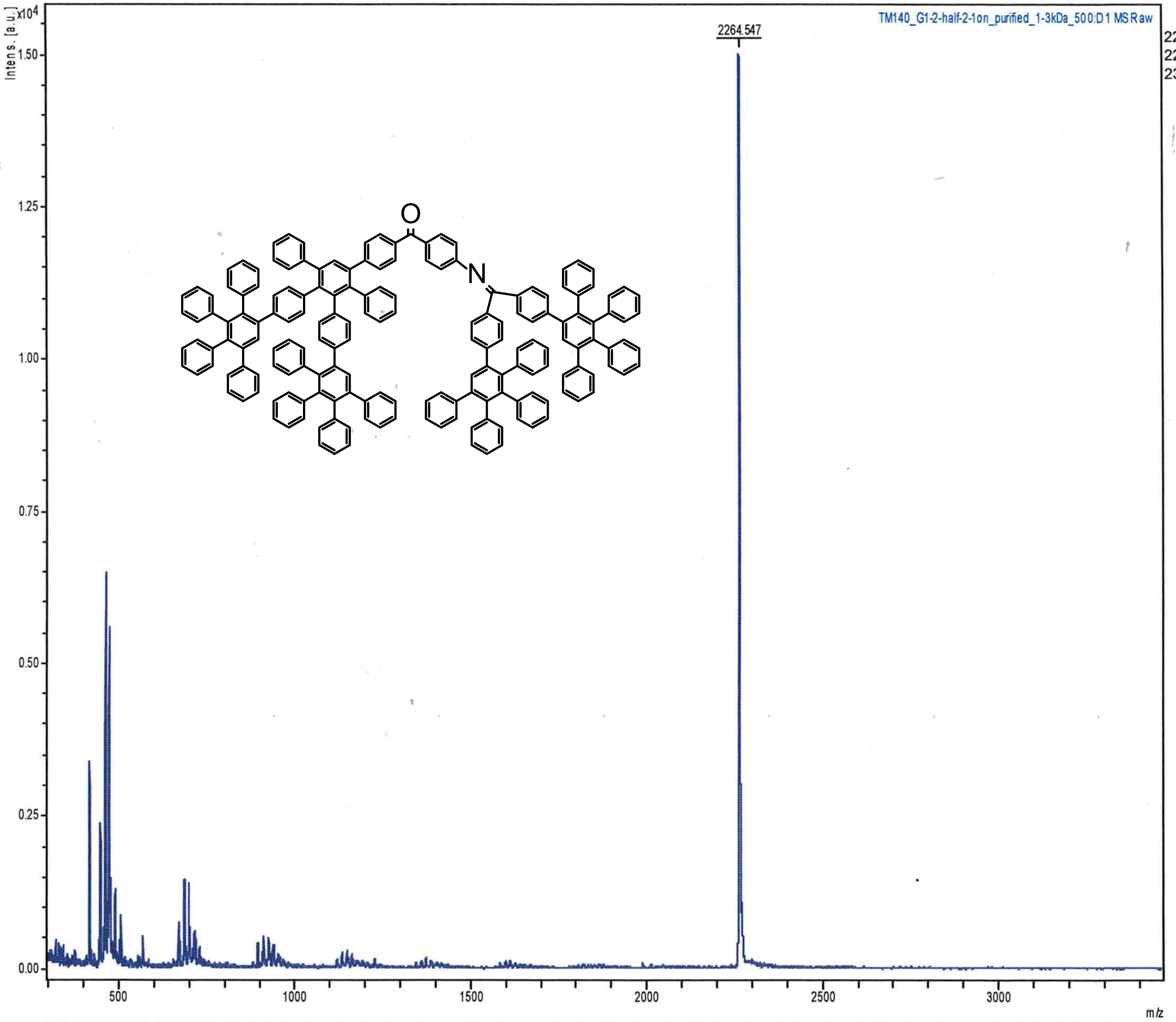

m/z	S/N	Quality Fac.	Res.	Intens.	Area
2264.547	662		681	15024	63421
2298.242	6		248	147	1872
2324.798	3		305	87.0	736

11/30/2018 2:35:40 PM

Fig C56. MALDI-TOF-MS spectra of compound 21.

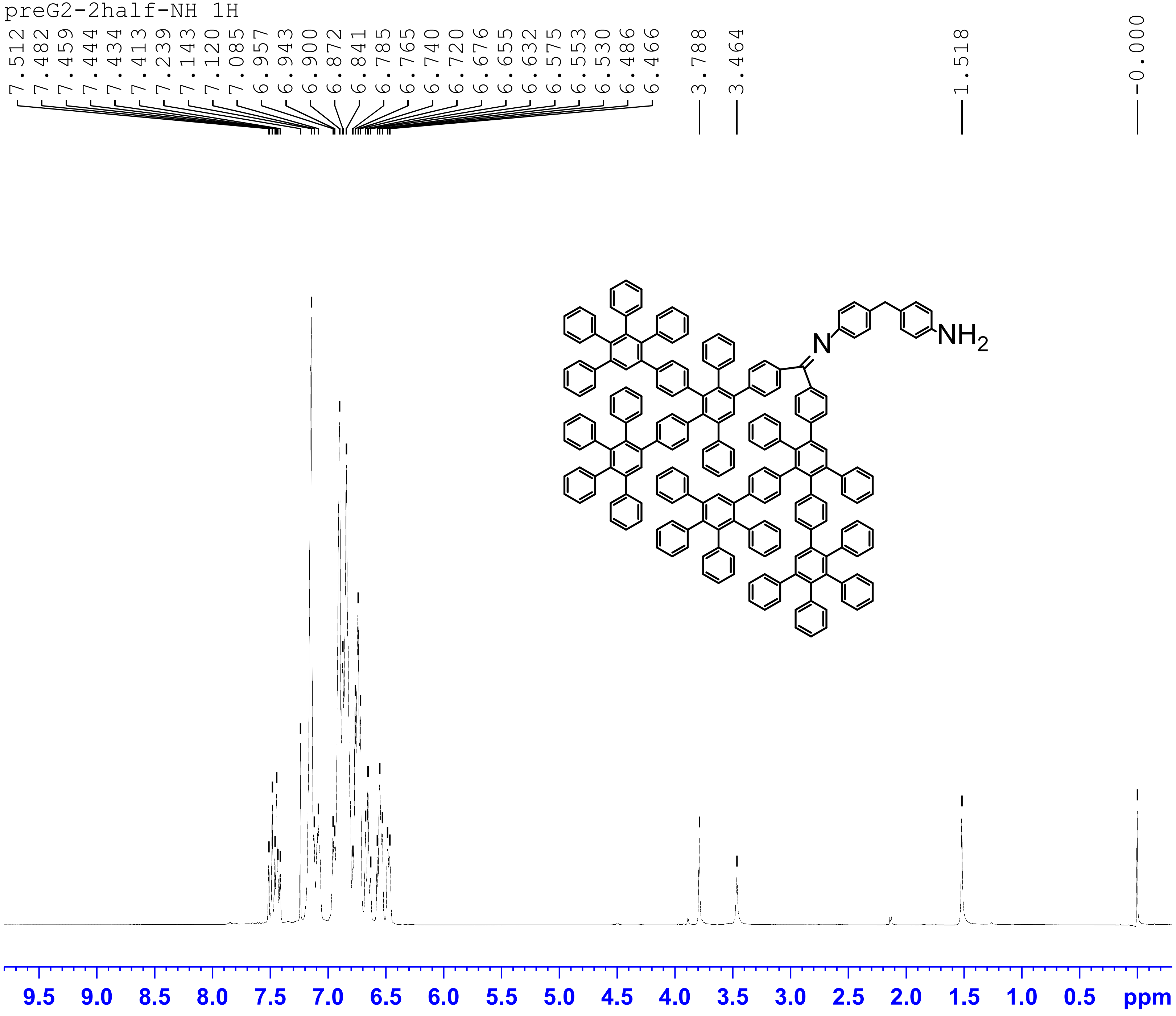

Current Data Parameters
NAME NMR
EXPNO 1271
PROCNO 1

F2 - Acquisition Parameters
Date_ 20180921
Time 3.16
INSTRUM spect
PROBHD 5 mm PABBO BB-
PULPROG zg30
TD 65536
SOLVENT CDCl3
NS 512
DS 2
SWH 8223.685 Hz
FIDRES 0.125483 Hz
AQ 3.9845889 sec
RG 63.61
DW 60.800 usec
DE 6.50 usec
TE 298.4 K
D1 1.00000000 sec

======== CHANNEL f1 ========
NUC1 1H
P1 9.00 usec
PLW1 23.79999924 W
SFO1 400.1324710 MHz

F2 - Processing parameters
SI 65536
SF 400.1300183 MHz
WDW EM
SSB 0
LB 0.30 Hz
GB 0
PC 1.00

Fig C57 1H-NMR spectra of compound 22.

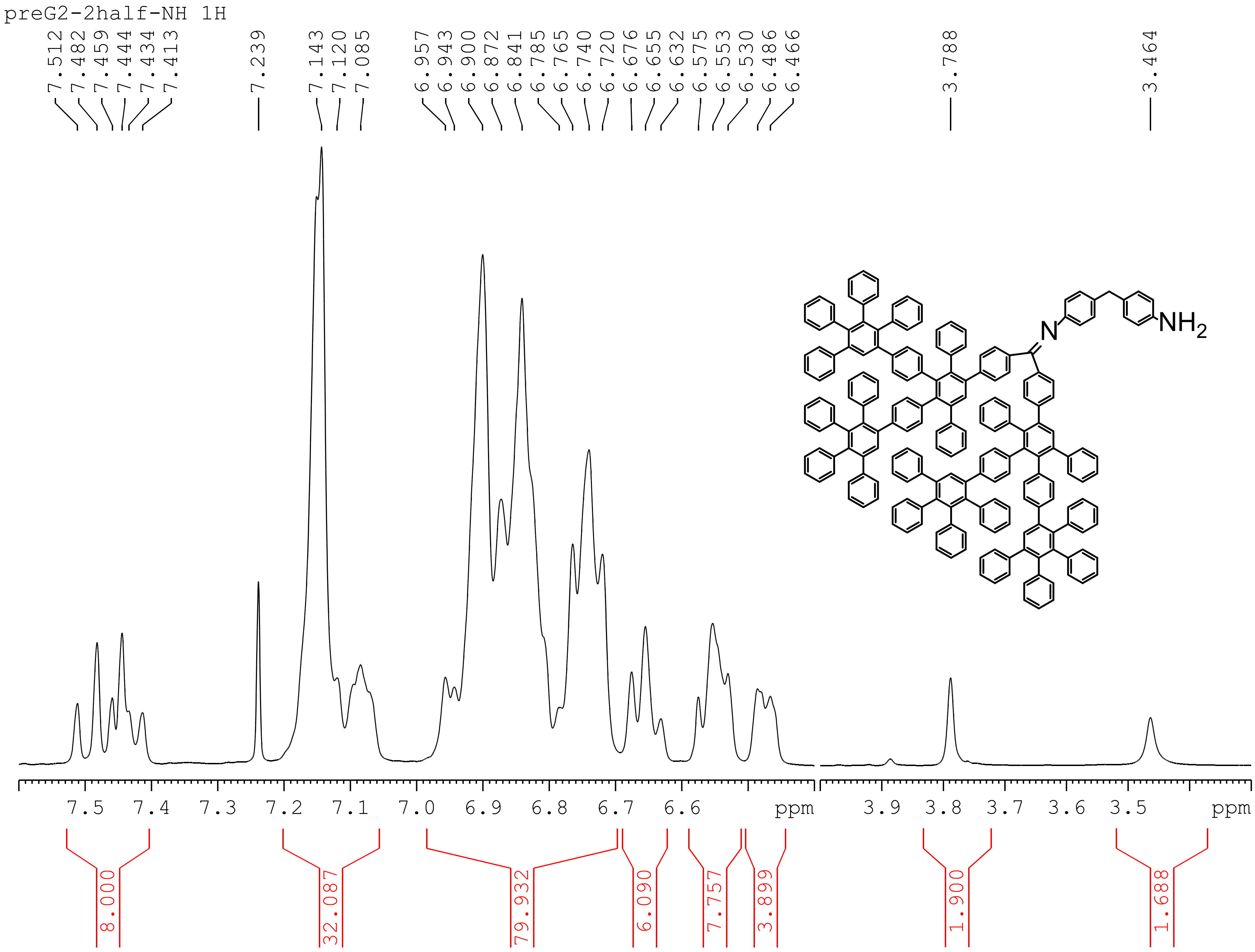

Fig C58. 1H-NMR spectra of compound 22.

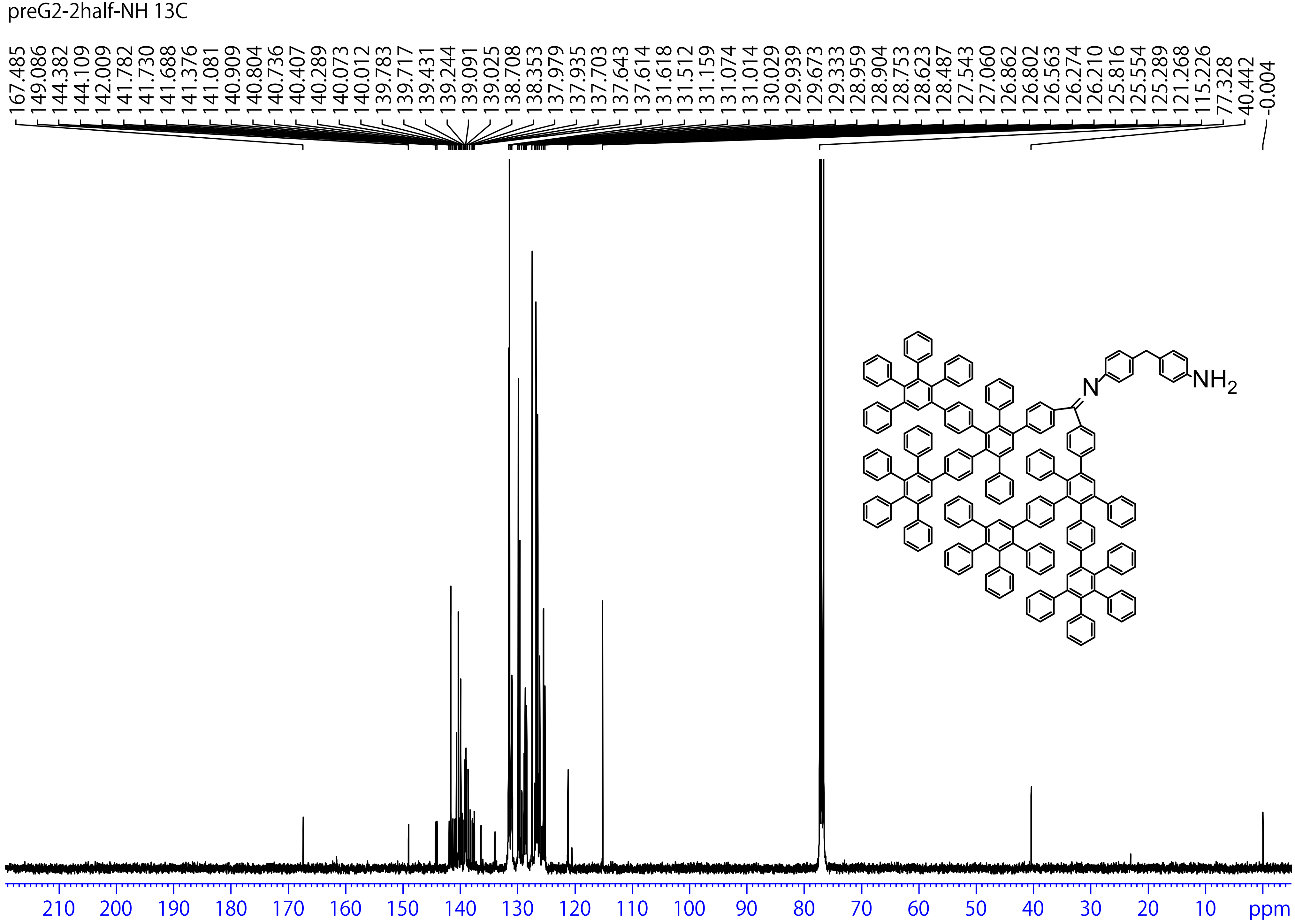

Fig C59. 13C-NMR spectra of compound 22.

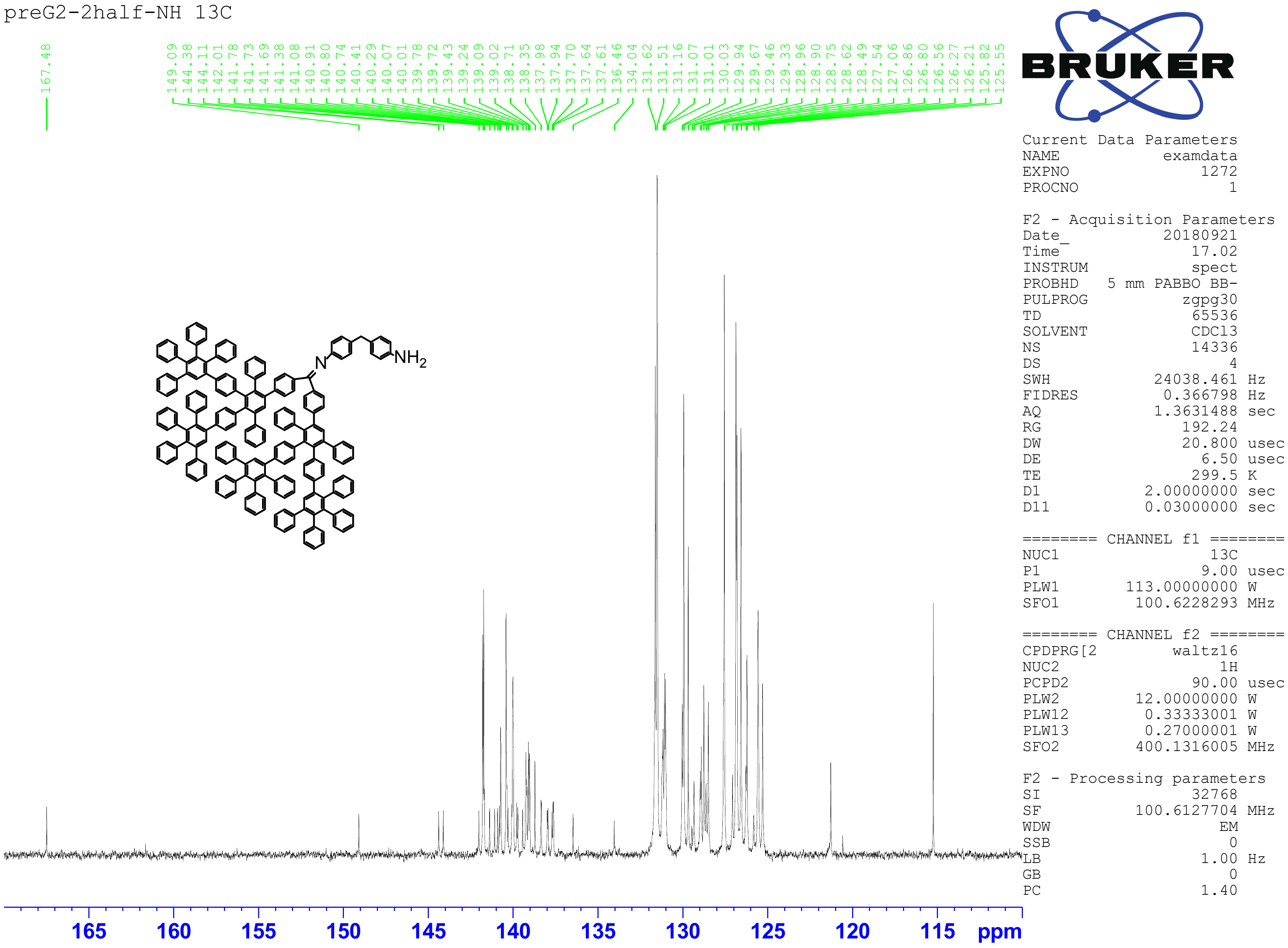

Fig C60. 13C-NMR spectra of compound 22.

[M+H] 2646.13 m/z

20180918 \ TM190_preG2-2half-NH_GPC_F2_1-3kDa_60

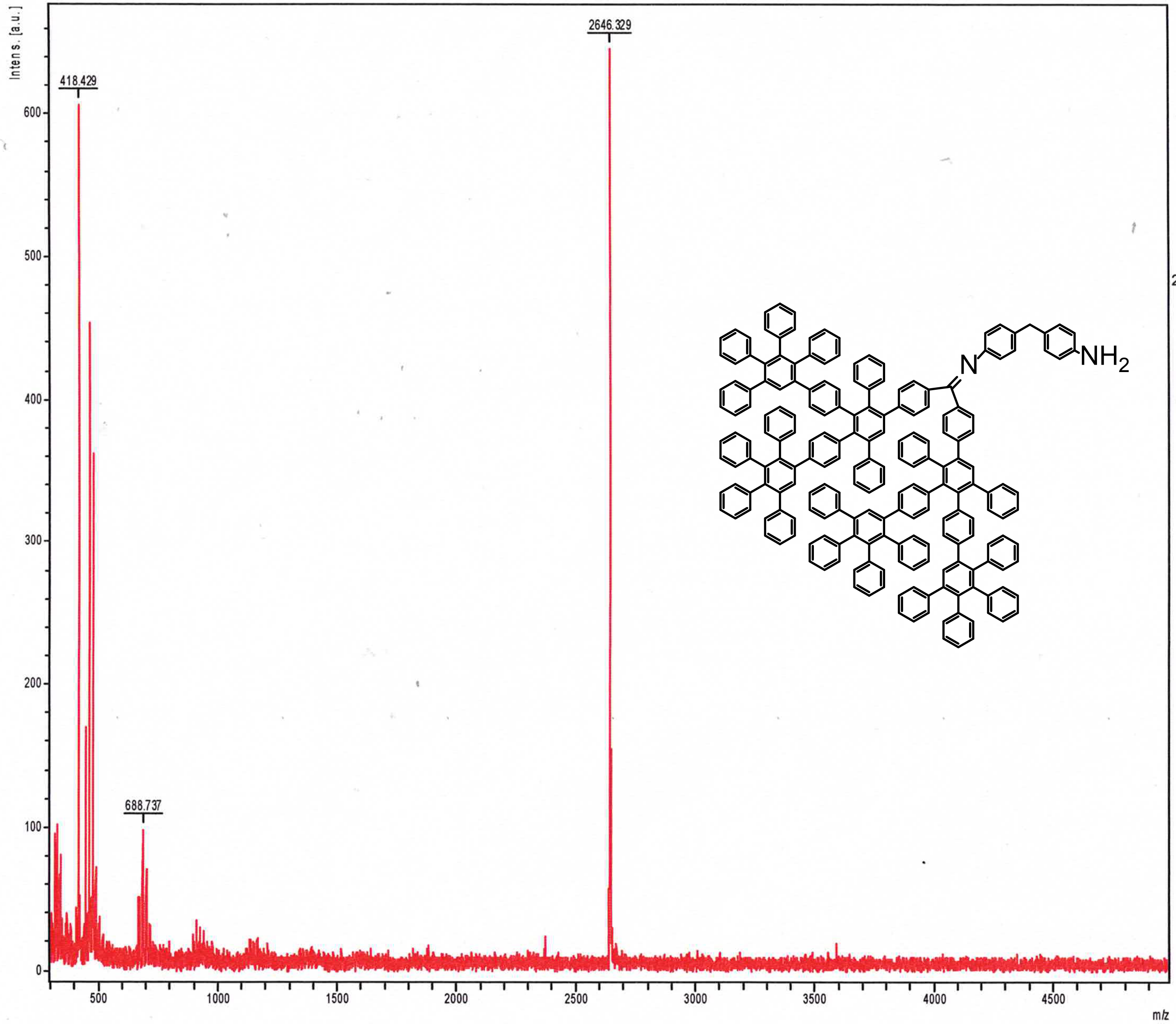

m/z	S/N	Quality Fac.	Res.	Intens.	Area
322.957	5		132	96.0	115
330.044	5		597	102	69.6
339.101	3		776	68.0	45.6
345.237	4		633	81.0	70.8
418.429	39		606	606	709
449.226	11		184	170	339
464.210	30		317	454	828
477.266	24		184	362	923
491.270	4		115	73.0	197
491.270	4		115	73.0	197
673.702	4		272	52.0	142
688.737	8		364	99.0	253
702.799	5		287	71.0	267
2646.329	76		682	646	2637

10/3/2018 1:51:20 PM

Fig C61. MALDI-TOF-MS spectra of compound 22.

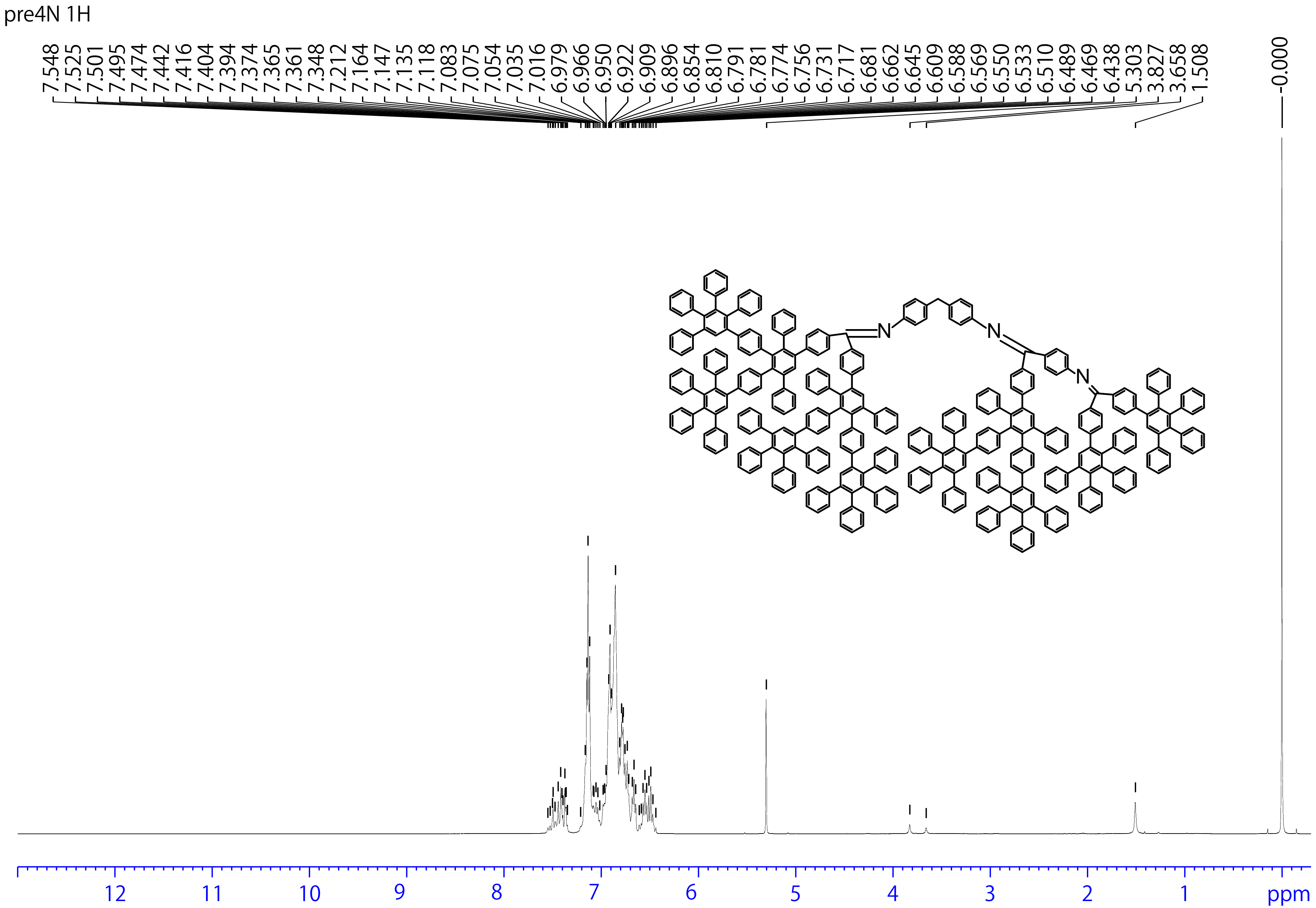

Fig C62. 1H-NMR spectra of compound 23.

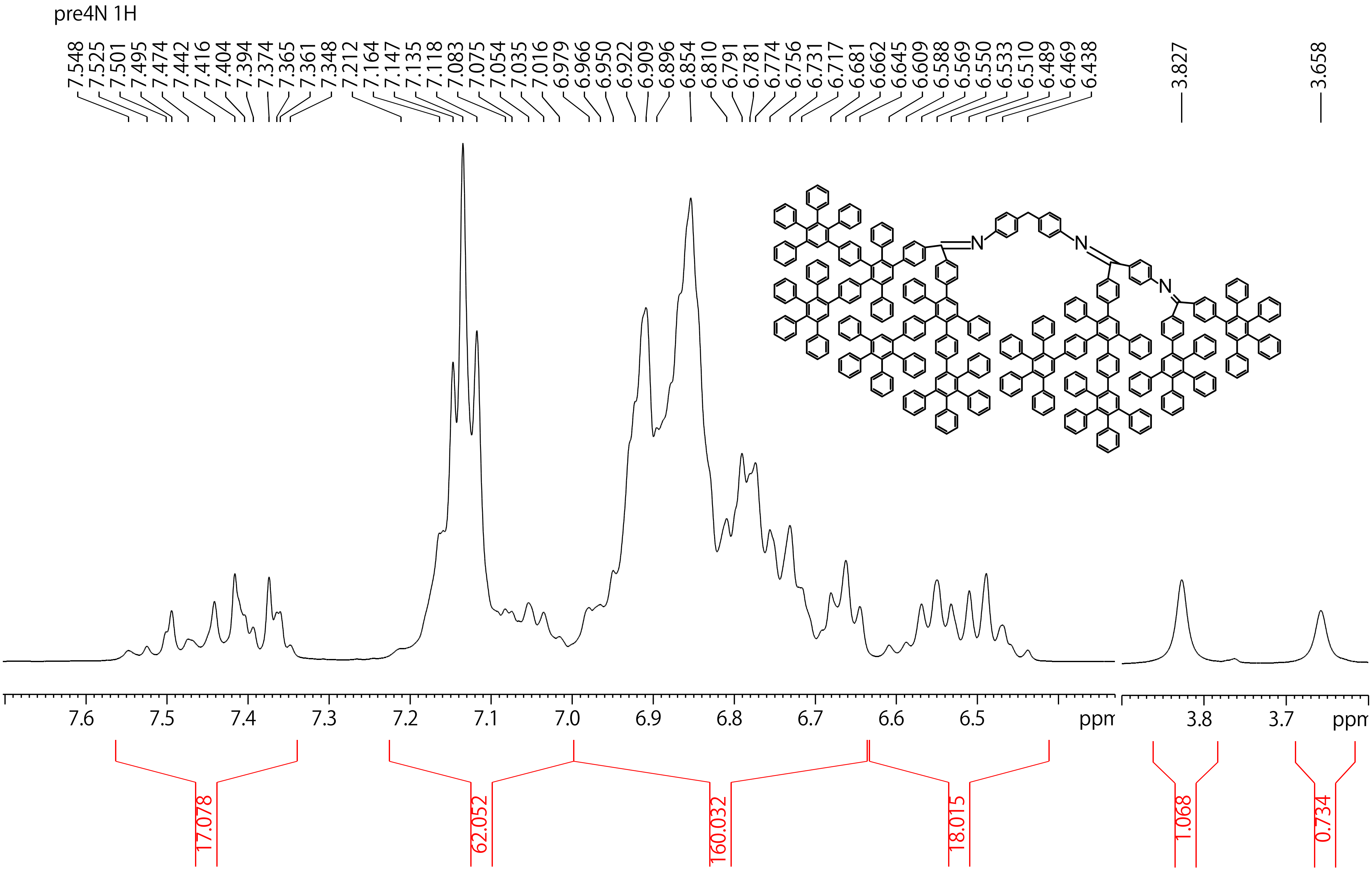

Fig C63. 1H-NMR spectra of compound 23.

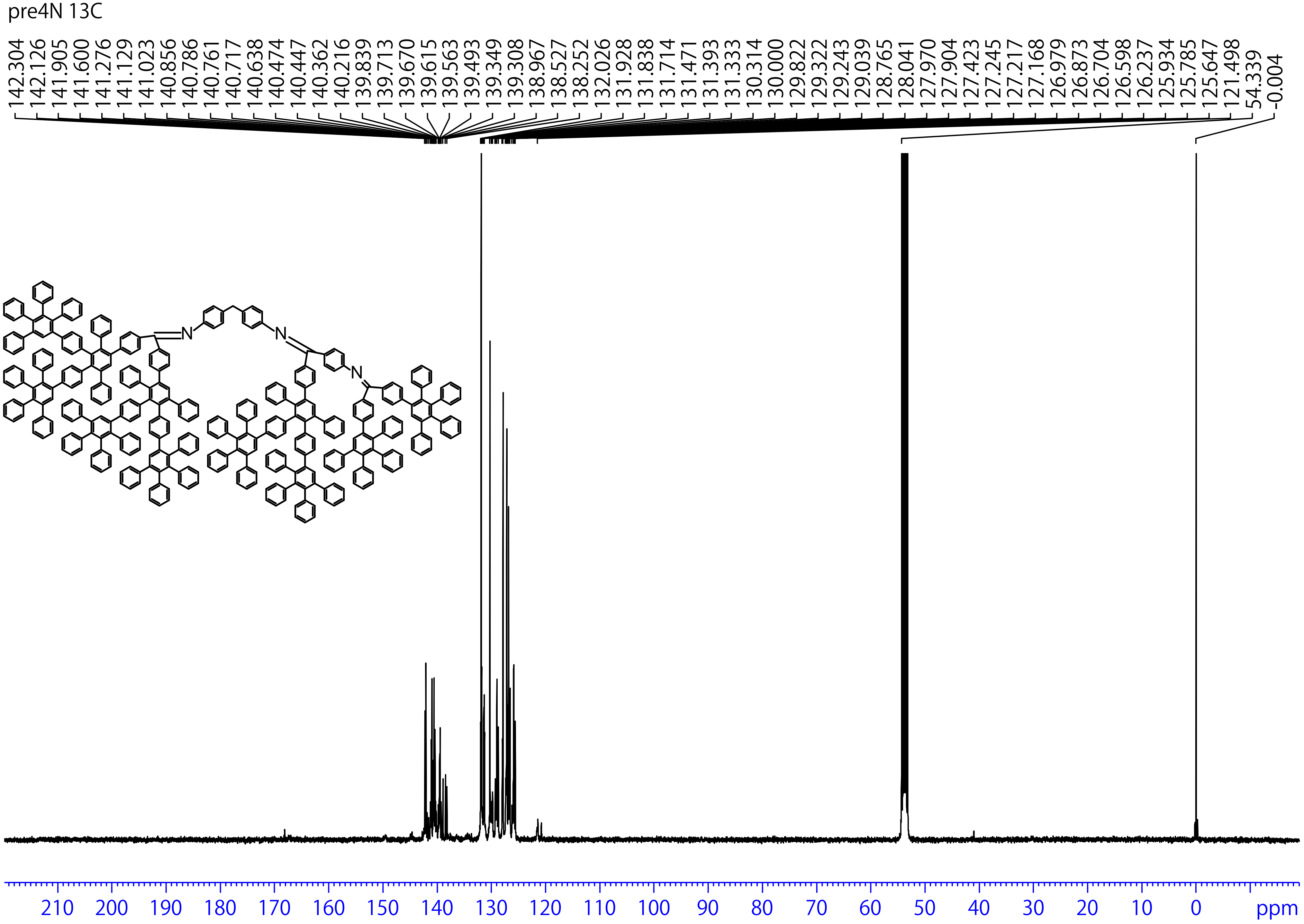

Fig C64. 13C-NMR spectra of compound 23.

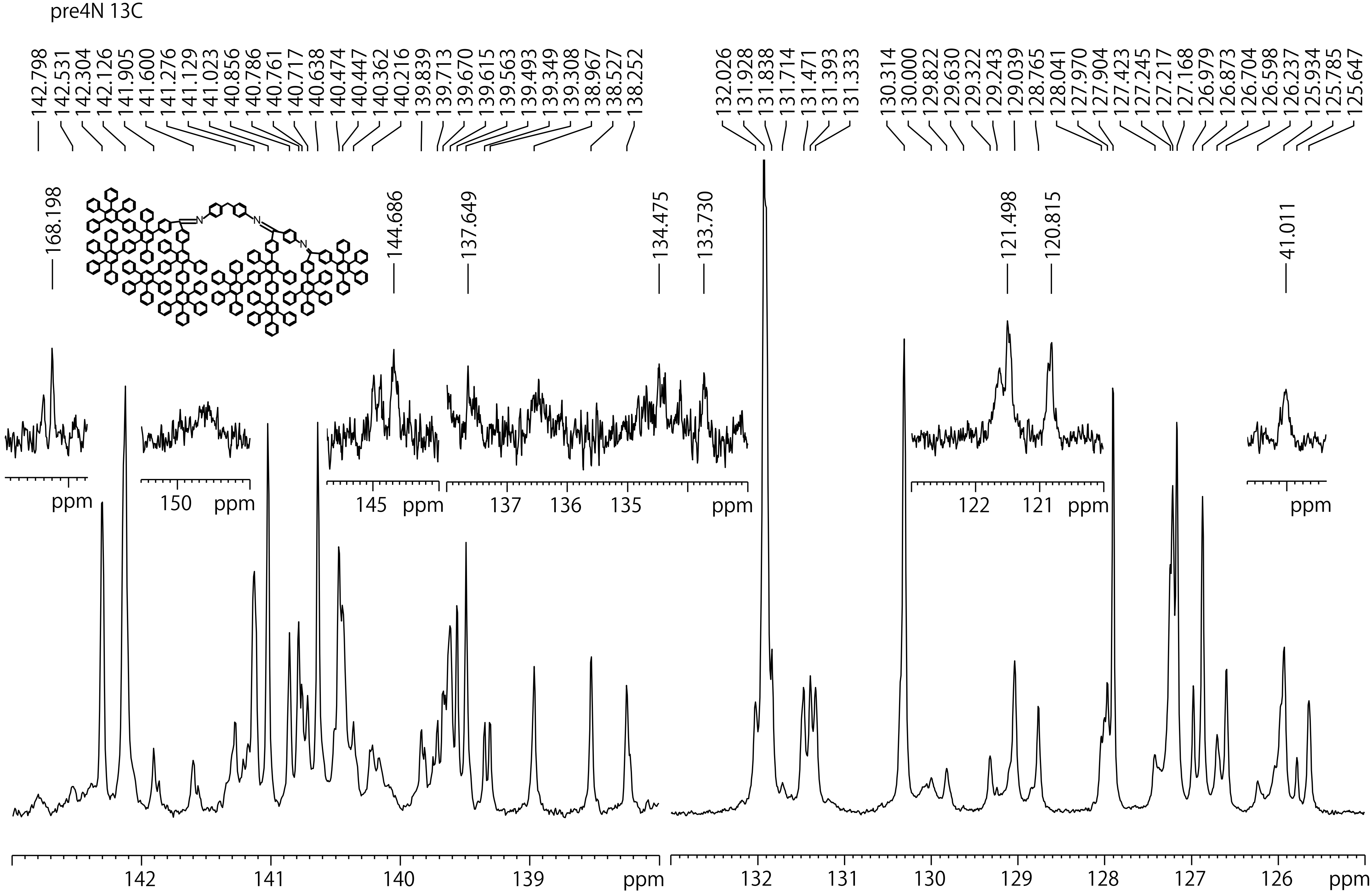

Fig C65. 13C-NMR spectra of compound 23.

[M+H] 4892.06

26

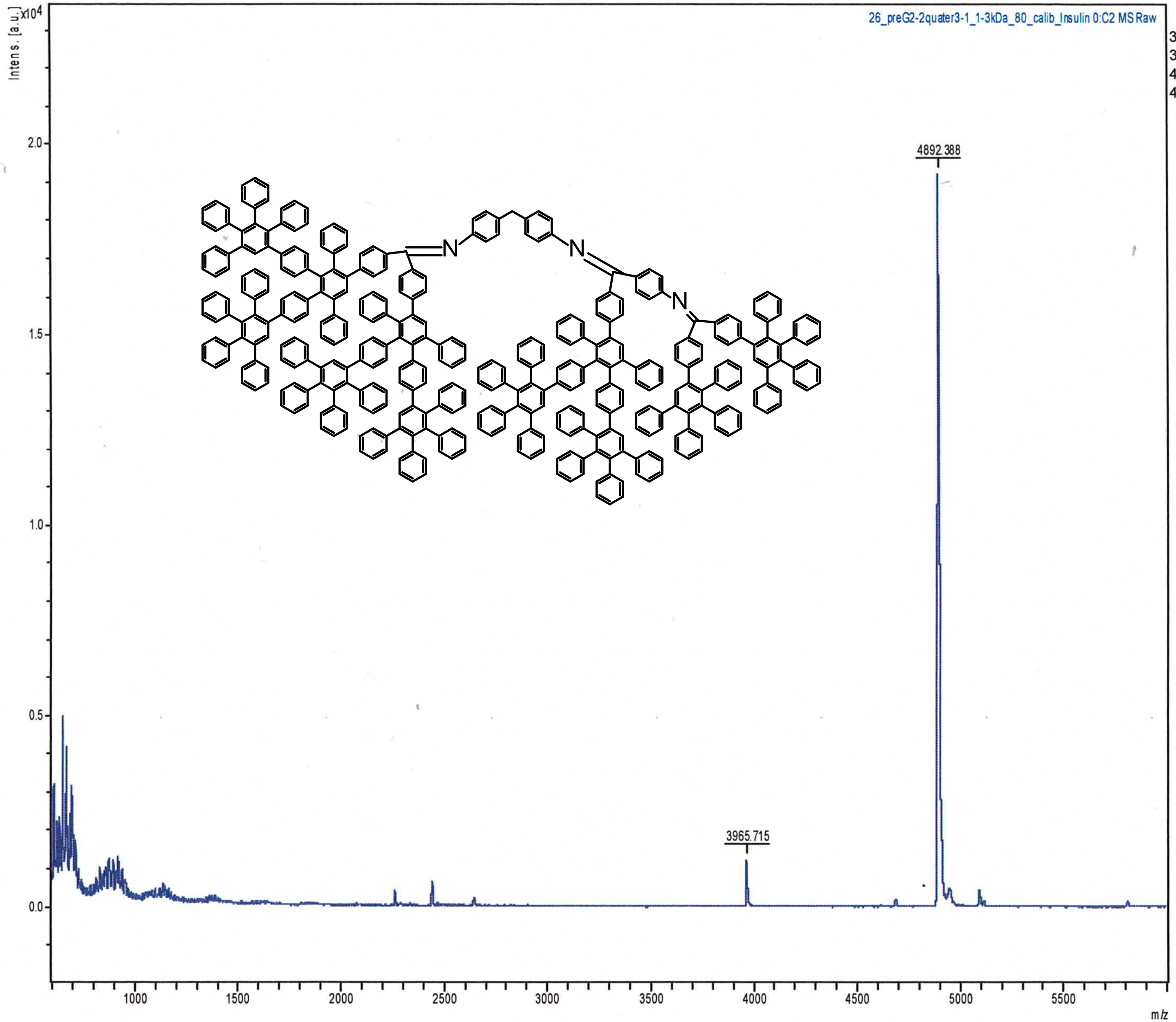

m/z	S/N	Quality Fac.	Res.	Intens.	Area
3965.715	98		572	1251	10355
3990.650	3		543	50.0	374
4892.388	1215		439	19206	258031
4948.419	31		264	497	13364

11/30/2018 5:36:30 PM

Fig C66. MALDI-TOF-MS spectra of compound 23.

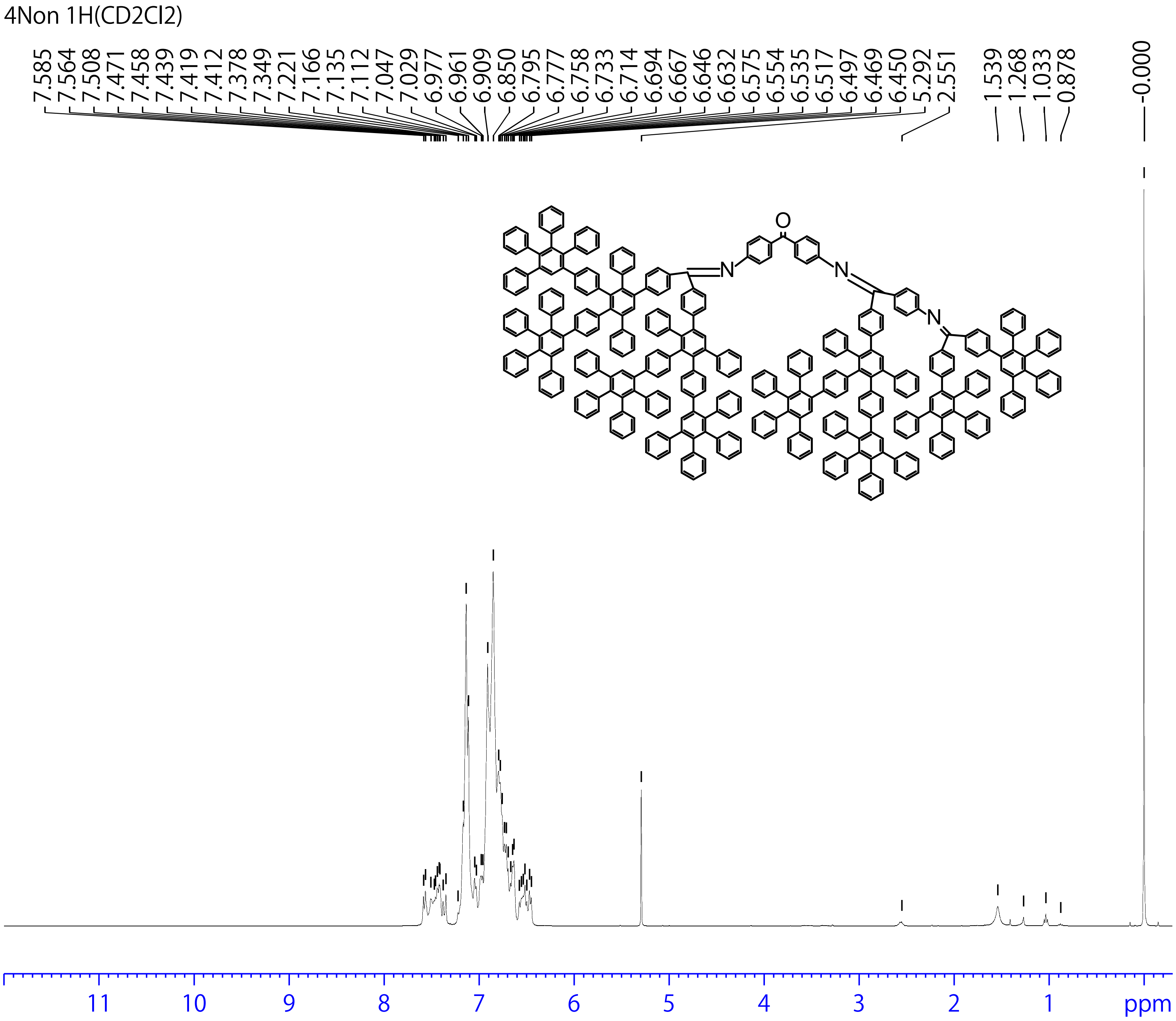

Current Data Parameters
NAME NMR
EXPNO 1181
PROCNO 1

F2 - Acquisition Parameters
Date_ 20180901
Time 0.48
INSTRUM spect
PROBHD 5 mm PABBO BB-
PULPROG zg30
TD 65536
SOLVENT CD2Cl2
NS 1024
DS 2
SWH 8223.685 Hz
FIDRES 0.125483 Hz
AQ 3.9845889 sec
RG 52.03
DW 60.800 usec
DE 6.50 usec
TE 299.0 K
D1 1.00000000 sec

======== CHANNEL f1 ========
NUC1 1H
P1 9.00 usec
PLW1 23.79999924 W
SFO1 400.1324710 MHz

F2 - Processing parameters
SI 65536
SF 400.1300266 MHz
WDW EM
SSB 0
LB 0.30 Hz
GB 0
PC 1.00

Fig C67. 1H-NMR spectra of compound 24.

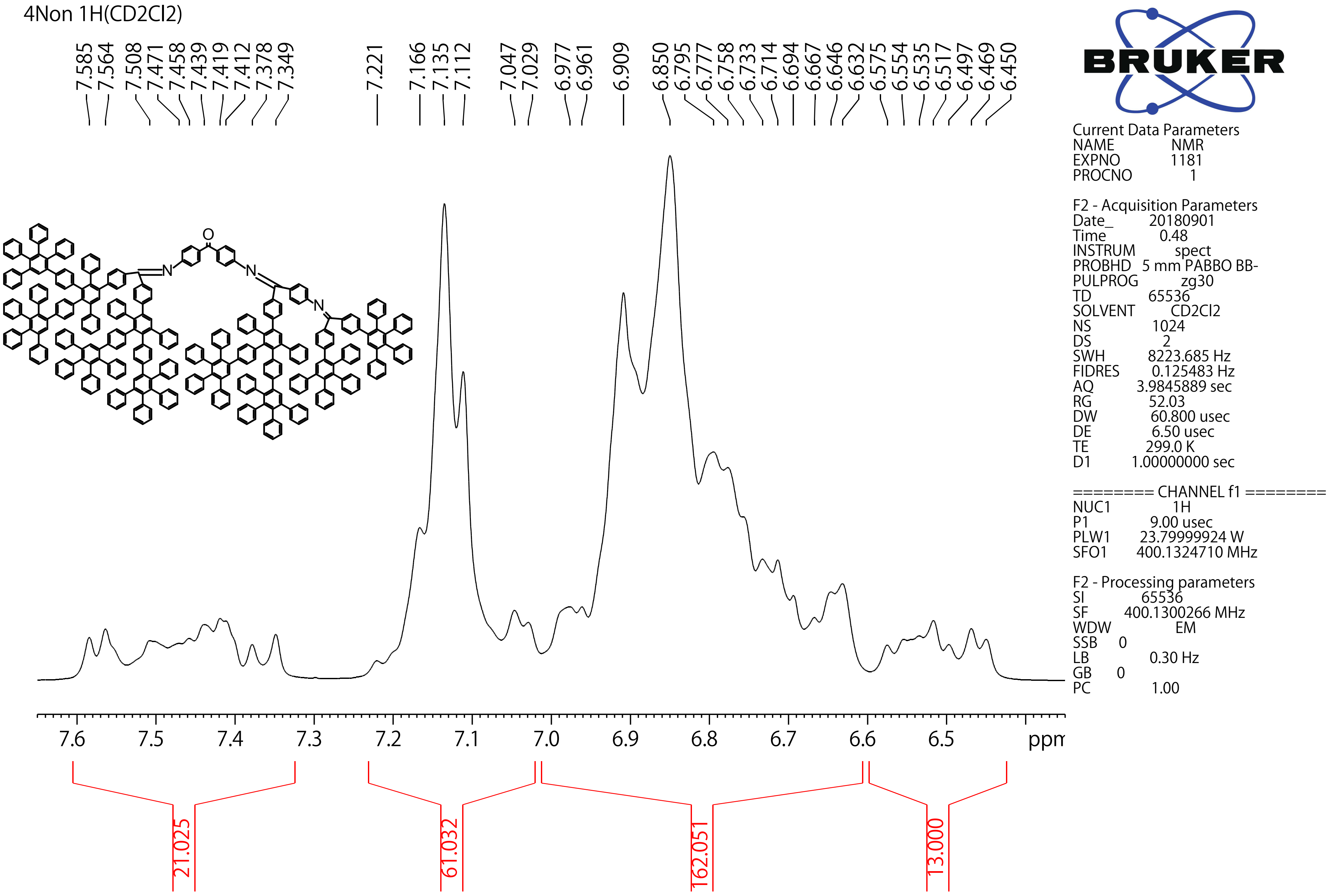

Fig C68. 1H-NMR spectra of compound 24.

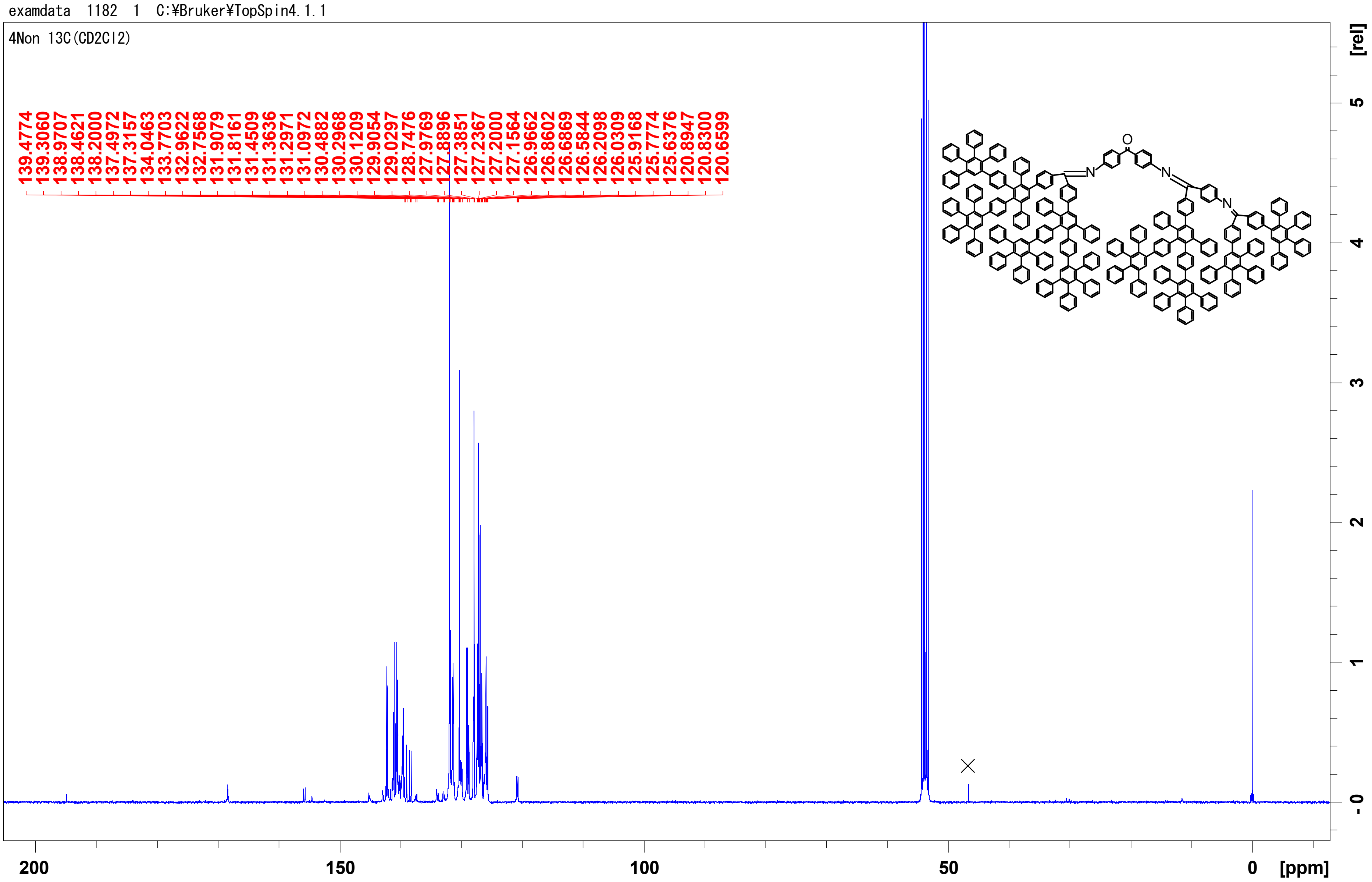

Fig C69. 13C-NMR spectra of compound 24.

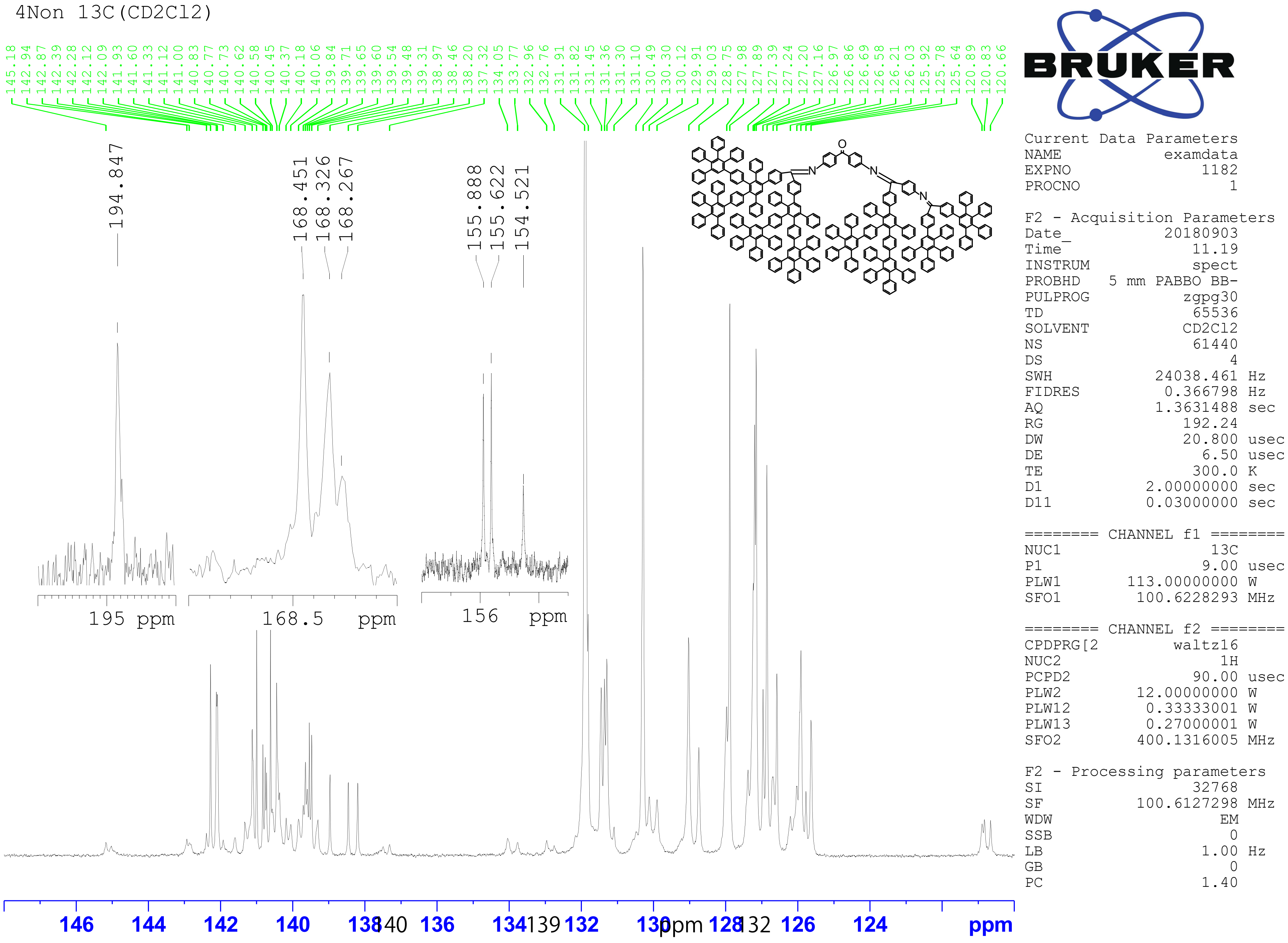

Fig C70. 13C-NMR spectra of compound 24.

[M+H] 4906.04

(27)

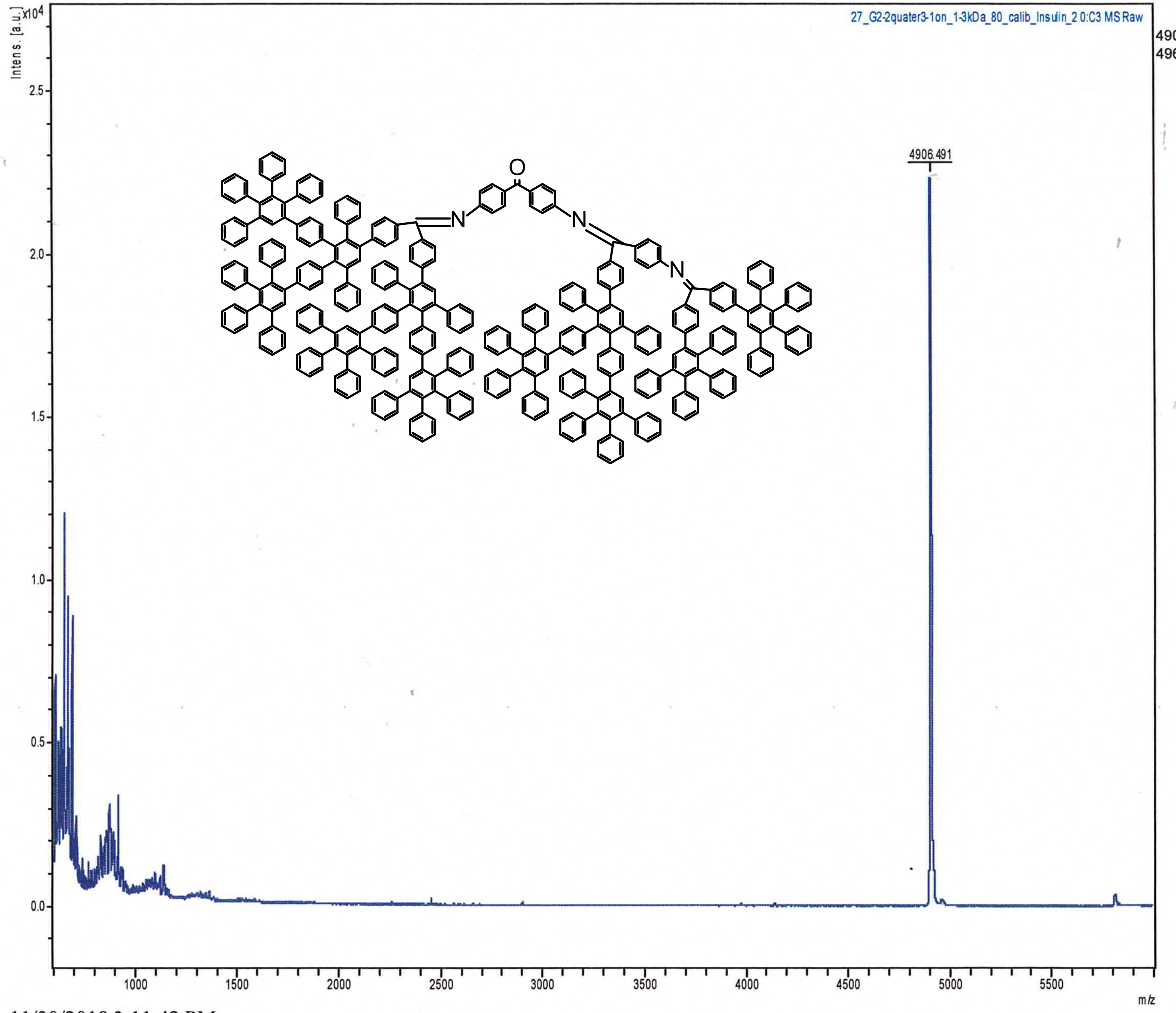

m/z	S/N	Quality Fac.	Res.	Intens.	Area
4906.491	1884		657	22326	200820
4962.823	16		312	194	4357

11/30/2018 3:11:42 PM

Fig C71. MALDI-TOF-MS spectra of compound 24.

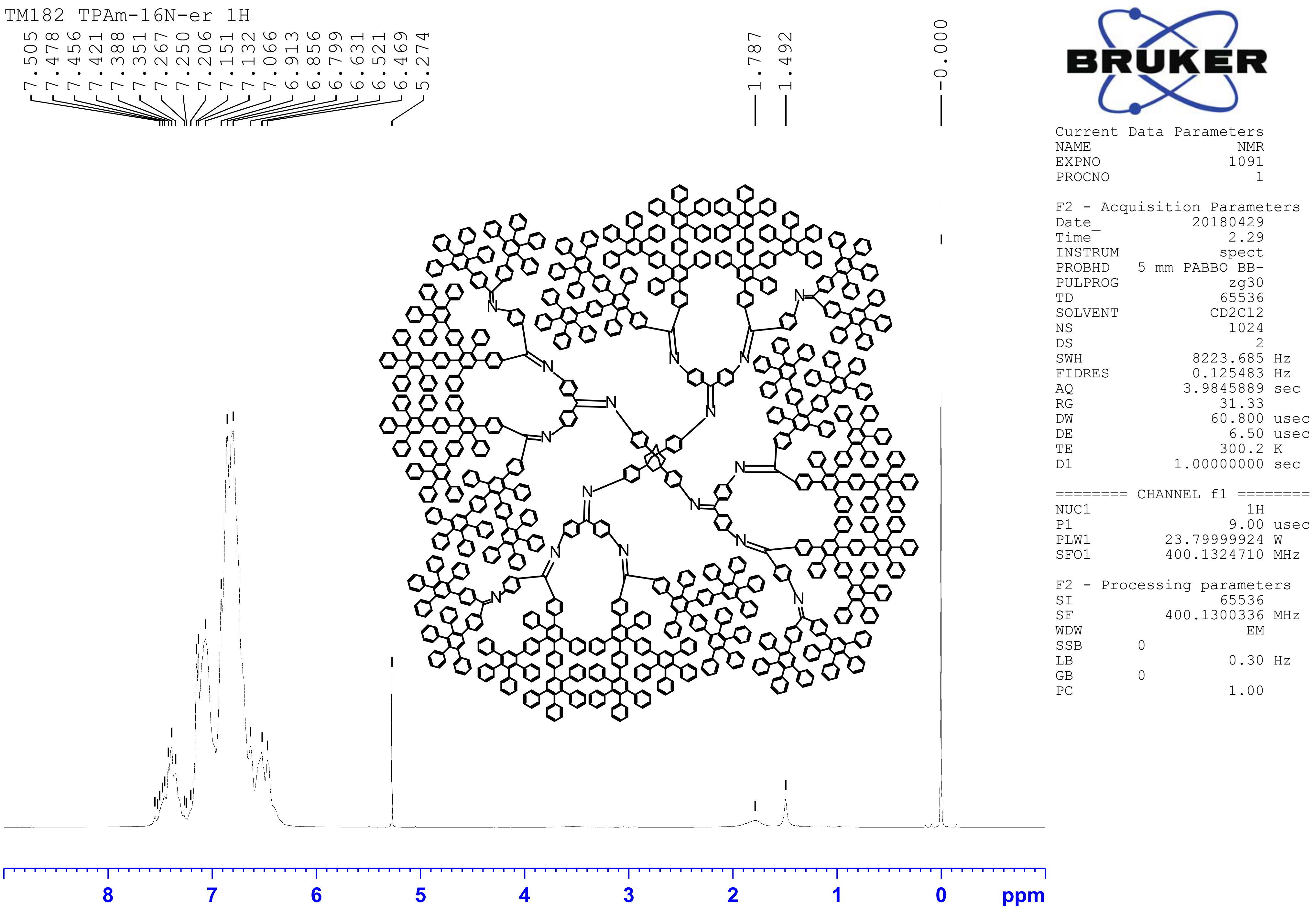

Fig C72. 1H-NMR spectra of PPDPA16.

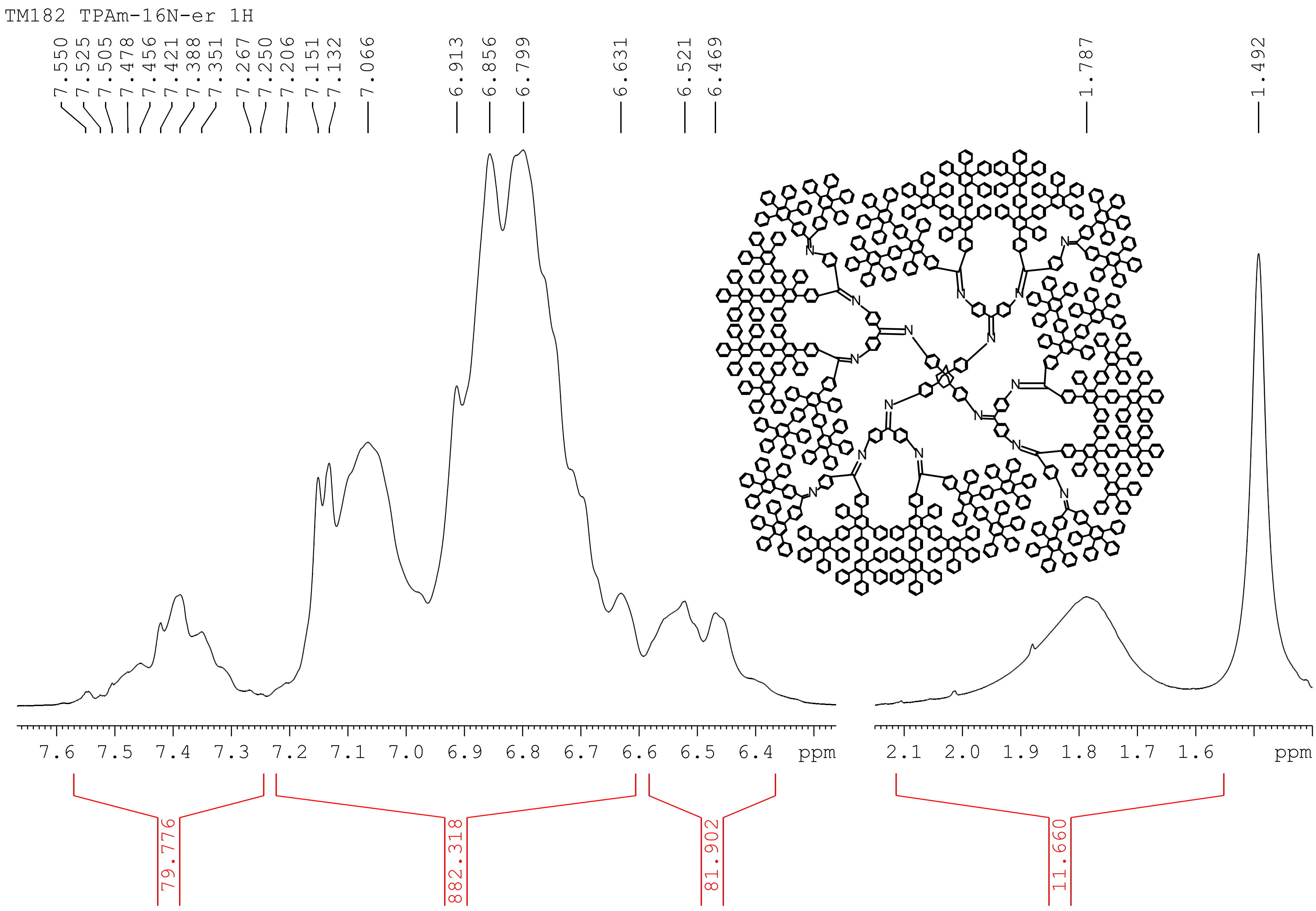

Fig C73. 1H-NMR spectra of PPDPA16.

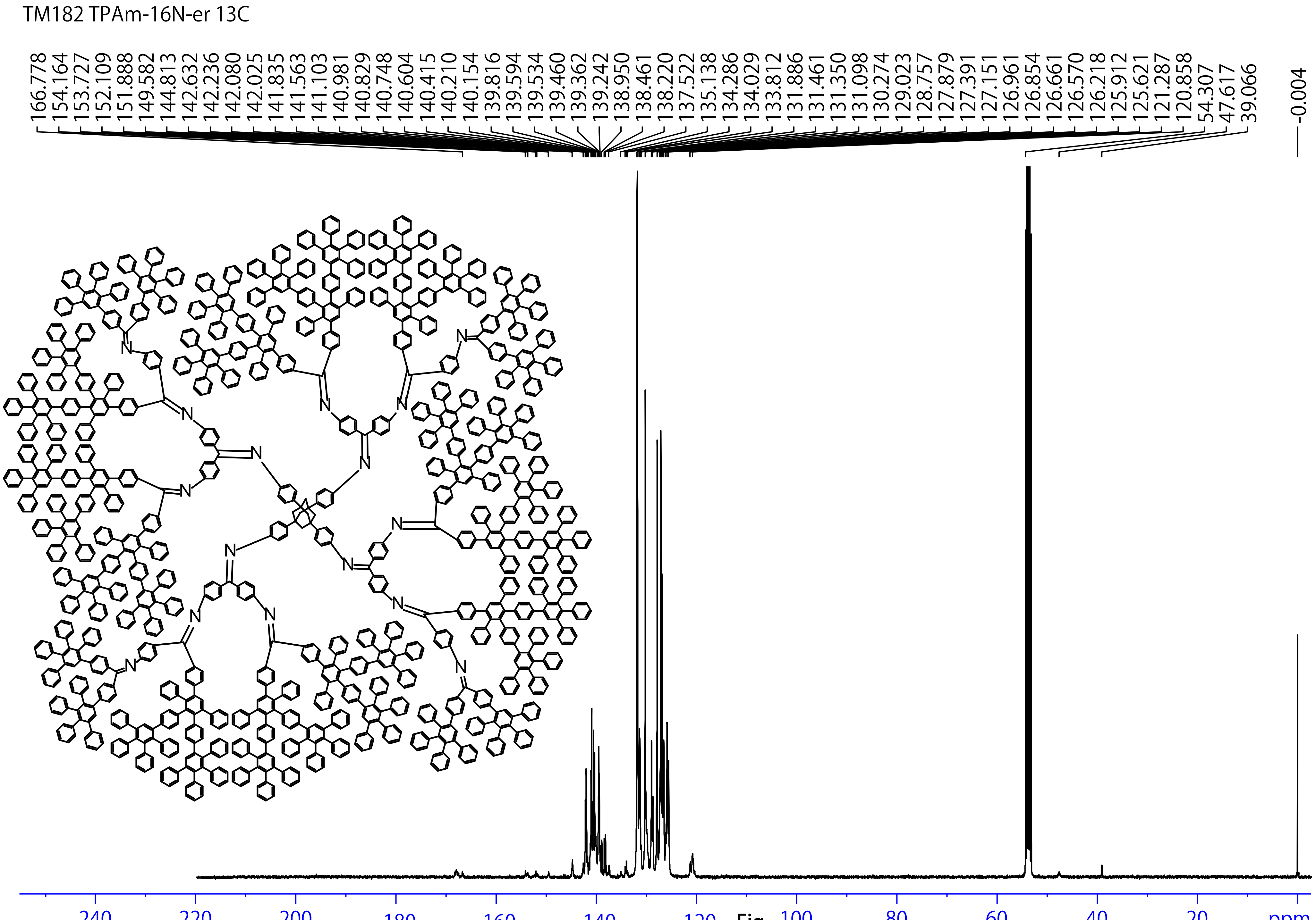

Fig C74. 13C-NMR spectra of PPDPA16.

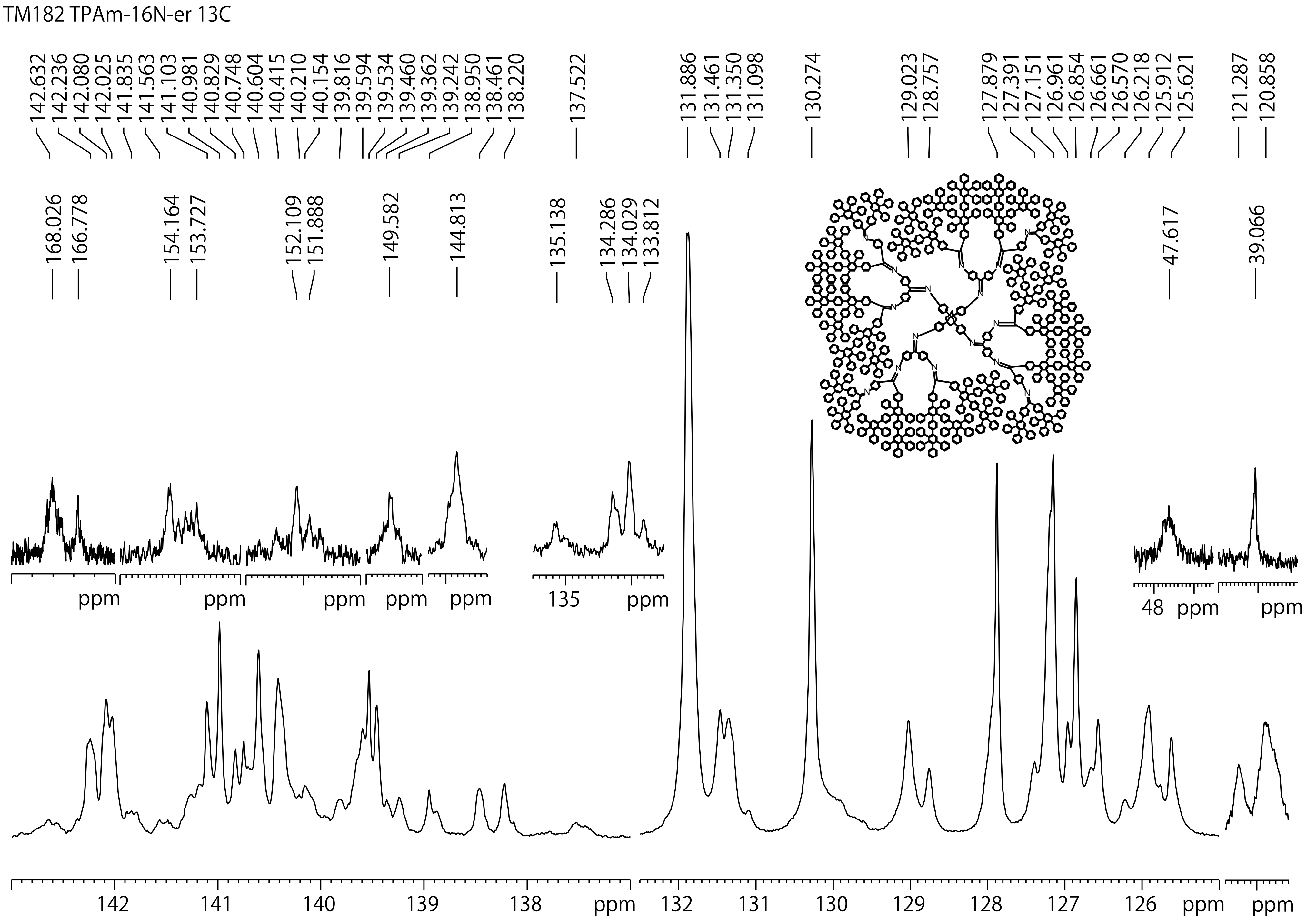

Fig C75. 13C-NMR spectra of PPDPA16.

20171117\ [illegible] TPAm-4(4N)_GPC2-5_07 [M+H] 20049.37

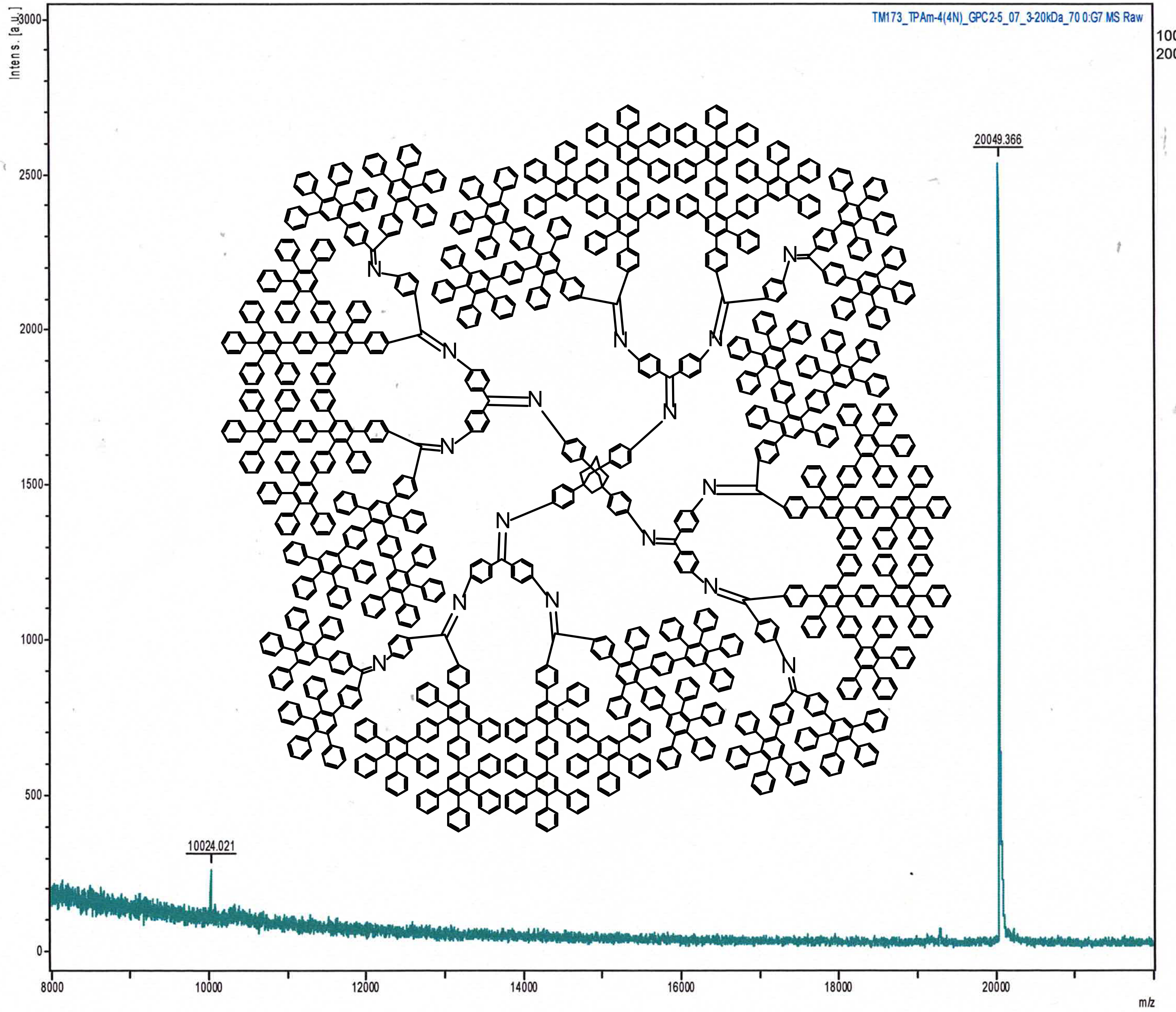

m/z	S/N	Quality Fac.	Res.	Intens.	Area
10024.021	6		1136	262	1627
20049.366	137		1345	2538	50645

10/4/2018 6:01:41 PM

Fig C76. MALDI-TOF-MS spectra of PPDPA16.
